# Chaotic Itinerancy in Collective Behaviour Emerging from Active Inference: A Multi-Agent Model of Trust and Empowerment Dynamics in Theatre Workshops

**DOI:** 10.3390/e28050491

**Published:** 2026-04-24

**Authors:** Shoko Miyano, Takashi Shiono

**Affiliations:** 1 College of Arts and Sciences, J. F. Oberlin University, Machida 194-0294, Japan; 2 Department of Economics, University of Tokyo, Tokyo 113-8654, Japan

**Keywords:** active inference, chaotic itinerancy, multi-agent systems, trust dynamics, free energy principle, theatre workshops

## Abstract

Chaotic itinerancy—irregular switching among metastable collective states—provides a dynamical substrate for flexible social coordination, yet its mechanistic origin in multi-agent systems remains unclear. We present a multi-agent Active Inference model in which chaotic itinerancy emerges from Expected Free Energy minimisation without outcome-level social priors. Agents select actions to minimise Expected Free Energy while updating preferences through a precision-gated learning mechanism modulated by interpersonal trust. Hill-function nonlinearity in state transitions creates bistable “affordance landscapes” that gate behavioural mode switching. Simulations with small number of agents on an Erdős–Rényi trust network reveal spontaneous alternation among multiple metastable behavioural clusters, heavy-tailed dwell-time distributions, and sign-changing finite-time Lyapunov exponents—three hallmarks of chaotic itinerancy. Crucially, replacing Hill-function dynamics with linear transitions reduces the chaotic-itinerancy detection rate from 80% to 20%, demonstrating that nonlinear affordance structure is necessary for generating metastable switching. We further show that agents with simplified internal models of the world sustain richer itinerant dynamics as a group than “perfect-foresight” agents, suggesting that bounded rationality may be functionally advantageous for maintaining behavioural flexibility. These results establish active inference as a principled framework for modelling chaotic itinerancy in social systems and offer a computational account of trust-mediated collective transitions observed in theatre workshops and group dynamics.

## 1. Introduction

Understanding how abrupt collective shifts emerge from individual decision-making constitutes a central problem in complex social systems research. Many social phenomena exhibit nonlinear transitions whereby gradual individual-level changes yield sudden macroscopic shifts, including cascades and coordination breakdowns [1,2].

The Free Energy Principle (FEP) describes biological agents as minimizing variational free energy [3]. Within this framework, Active Inference casts action selection as Expected Free Energy (EFE) minimisation, integrating goal-directed behaviour and epistemic exploration [4].

Extending Active Inference to social systems raises a familiar concern. Some models introduce explicitly social or normative priors that directly favor particular collective outcomes (“social priors”), which can risk circularity if the target regularities are effectively encoded in prior preferences [5,6]. We therefore ask whether chaotic itinerancy—structured switching among multiple metastable collective modes—can arise under EFE minimisation when agents have minimally social preferences but structured generative models.

In theatre workshops and experimental actor training practices, the relevant shift is not limited to changes in average participation but involves a qualitative transformation in how participants take relational risks and experience agency. Theatre theory and experimental practice have described such transformations in terms of the removal of habitual defenses (Grotowski) and entry into a precarious “empty space” in which action is no longer fully guided by established roles or expectations (Brook) [7,8]. Accordingly, we target chaotic itinerancy at the collective level—where groups alternate among multiple metastable modes before settling—and an individual-level learning mechanism that operationalizes “threshold crossing” through adaptive preferences.

Methodological stance: avoiding outcome-level social priors.

Active Inference social models sometimes place normative structure at the outcome or policy level. For example, Constant et al. formalise social conformity using *deontic value* and *deontic cues*, where observations can directly endow policies with normative salience (i.e., an observation-conditioned prior over “what one should do”) [5]. Relatedly, models of cooperative communication may posit an *adaptive prior belief* that agents’ mental states are aligned, and cast communicative acts as evidence gathering for that prior [6].

In this paper, we adopt a constraint that makes the explanatory target explicit. *We avoid* outcome-level priors that directly prescribe collective patterns (e.g., explicit conformity utilities or preferred participation outcomes). *Instead*, we place structure in latent-state dynamics and learning conditions (nonlinear collective effects and trust-gated preference plasticity) while keeping preferences over broadly non-normative state variables (trust, empowerment, stamina).

### 1.1. Why Theatre Workshops as a Model System for Social Interaction

Theatre performances serve as experimentally controllable microcosms of social coordination. This perspective is grounded in theatre theory and laboratory-based experimental practices that treat the rehearsal space as a laboratory for investigating human interaction.

According to Brook’s concept of the “empty space,” theatre can arise from the minimal configuration of a performer and a spectator. In this most condensed form, theatre makes visible the essential dynamics of human interaction—the continuous chain of action and reaction—by isolating them from the distractions of everyday social contexts [8].

Extending this experimental lineage, Grotowski’s “Laboratory Theatre” explicitly conceptualized performance as research into the actor–spectator relationship, emphasizing a “via negativa” approach: not the accumulation of expressive techniques, but the systematic removal of habitual psychological and physical defenses that constrain action. By stripping away social masks and conditioned responses, performance becomes a site for exposing underlying impulses and relational dynamics [7]. Subsequent computational work has modelled such dyadic interactions using generalised synchrony frameworks, demonstrating how coupled agents can achieve flexible coordination through synchronisation of chaos [9].

Boal further extended this trajectory by dissolving the boundary between actor and audience, framing theatre as a space in which participants can actively explore alternative modes of interaction [10].

Taken together, these theatre theories and experimental practices converge on a shared view of performance as an experimentally constrained setting for probing the fundamental dynamics of social interaction, where relational patterns can be isolated, transformed, and explored. Crucially, this experimental orientation shifts attention away from representational display toward the conditions under which interaction itself is generated.

Building on these perspectives, this paper focuses on theatre workshops rather than traditional theatrical performances. While conventional theatre foregrounds the interaction between performers and spectators, the workshops considered here deliberately minimise the element of being observed.

To clarify what is meant by a theatre workshop in this context, we briefly outline a typical structure below. Theatre workshops vary widely in form, and many are designed not for trained actors but for participants with little or no prior experience in theatre. A typical workshop for such participants often begins with introductory activities aimed at building interpersonal relationships, followed by stretching and movement-based exercises, and then incorporates a series of expressive activities using improvisational acting that foreground mutual responsiveness. This shift redirects participants’ attention away from presentation and evaluation and toward the ongoing dynamics of action and response among those involved. Collectively, these features highlight how theatre workshops deliberately isolate and intensify coordination under shared uncertainty, providing a structured setting in which collective phenomena can be examined.

In this sense, the theatre workshop functions as a structured experimental environment, allowing collective patterns of interaction to emerge through embodied engagement rather than scripted representation. These considerations motivate our use of theatre workshops as a concrete experimental system for formal modelling and serve as constraints on model design.

In particular, we operationalise “crossing a threshold” as an internally learned change in preference parameters: empowerment overshoots, when experienced under sufficient inferred trust, are consolidated through precision-weighted hierarchical Bayesian learning and persist after the perturbation ends. This provides a minimal account of how a group can shift from inhibited participation to a stable mode of expressive agency without prescribing the outcome.

### 1.2. Why Chaotic Itinerancy Is Essential in Modeling Theatre Workshops

Theatre workshops rarely converge to a single stable collective mode. Instead, groups often alternate among qualitatively different regimes—inhibited, exploratory, playful, confrontational—with irregular transitions that depend on interaction history. This pattern is well described by *chaotic itinerancy* (CI): trajectories that dwell near multiple quasi-stable “attractor ruins” and switch among them in a structured but unpredictable manner [11,12,13]. Notably, a connection between Active Inference and chaos control was established early in the FEP literature: prediction-error suppression under strong priors can itself generate and stabilise chaotic trajectories [14].

CI provides an interpretable vocabulary for small-group processes. Rather than assuming that theatre workshops must converge to either “success” or “failure,” CI emphasizes the transient, exploratory nature of collective dynamics: multiple metastable modes coexist, and facilitation may shape which modes are visited and in what sequence. This perspective aligns with theatre theory and experimental practice that value the process of exploration over fixed outcomes [7,8].

Contributions.

This paper makes two primary contributions:

- We demonstrate Emergent Chaotic Itinerancy in a multi-agent POMDP under Active Inference. This result successfully reproduces the dynamic process of theatre workshops—where groups alternate between periods of high energy and quiescence—providing a novel computational approach to theatre workshop analysis that has not been seen in previous studies.
- We introduce Precision-Gated Preference Learning as a natural implementation of preference inference within Active Inference, enabling the agent’s preference model to be discontinuously updated by large surprise events via a hierarchical Bayesian mechanism.

To realise these contributions, the model incorporates the following distinctive features:

- Hill Function Nonlinearity: We embed cooperative threshold effects via the Hill function in state transition dynamics, which enables chaotic itinerancy to emerge. Ablation experiments confirm that removing this nonlinearity (reducing to a linear AR(1) process) eliminates chaotic itinerancy entirely, demonstrating that the Hill function is the essential mechanism generating complex collective dynamics.
- Interpersonal Trust Network: We extend mean-field dynamics to a sparse Erdős–Rényi graph where dyadic trust evolves based on action synchrony and local state variables are spatially averaged, enabling heterogeneous social perception.
- Robust Parameter Regime: Systematic scans confirm that the qualitative behaviour is robust across a wide range of parameters.

We study a stylized setting inspired by theatre workshops, where participants decide whether to express themselves creatively under partial observability and resource constraints. The same formal structure can be applied to other social systems in which a shared latent context (here: trust) interacts with individual agency and fatigue. The remainder of the paper specifies the POMDP model and EFE-based policy, reports numerical evidence for chaotic itinerancy, and discusses implications and empirical extensions.

## 2. Related Work on Collective Phase Transitions in Small-Group Systems

### 2.1. Sociophysics and Ising-Type Perspectives on Collective Transitions

A large body of work in sociophysics and statistical-physics-inspired social modelling has demonstrated that sharp collective transitions, multistability, and hysteresis can arise from simple local interaction rules, including Ising-like binary-choice dynamics and threshold/cascade models [1,2]. These models provide an important baseline vocabulary for understanding phase-transition-like phenomena in social systems. However, they are typically formulated either as population-level stochastic dynamics with externally specified update rules (e.g., Glauber-type flips), or as utility-like response functions, rather than as agents performing principled inference and planning under a generative model (i.e., explicit modelling of agency).

### 2.2. Small-Group Dynamical Models with Metastable Collective States

Closer to the scale of workshops and therapeutic groups, quantitative small-group models have explicitly investigated the emergence and alternation of metastable collective states. Notably, Lauro Grotto et al. proposed an analytic dynamical-systems model of interacting agents (with simplified emotional/cognitive variables) and studied when metastable group-level states emerge, how these states differ structurally, and how system size affects the dynamics [15]. This line of work is especially relevant because it targets the “small-N” regime (comparable to workshop groups) and treats the group as a setting in which qualitatively distinct collective modes can persist and switch. In a related vein, Kelso and collaborators have established a rigorous dynamical-systems framework for coordination in small groups, demonstrating that metastability—the coexistence of integrative and segregative tendencies—is a generic feature of coupled oscillator systems even at small *N* [16,17,18].

### 2.3. Psychotherapy and Therapeutic Change as Nonlinear Dynamics and Critical Transitions

Independent of theatre, psychotherapy research has developed a mature complex-systems tradition in which therapeutic change is treated as nonlinear, sometimes discontinuous, and potentially characterized by critical instabilities and phase-transition-like shifts. A representative mathematical approach is the dynamical model of psychotherapy proposed by Liebovitch et al. [19]. Empirically and conceptually, the synergetic/complex-systems program associated with Schiepek and collaborators emphasizes self-organisation, critical fluctuations, and abrupt transitions in therapy process time series [20,21,22]. These studies provide precedent for treating “intervention + interaction history” as capable of moving a social–therapeutic system across qualitative regimes, i.e., path dependence. Complementary empirical work by Tschacher and collaborators has documented nonverbal synchrony between therapist and client as a measurable correlate of therapeutic alliance and has linked interpersonal coordination dynamics to treatment outcomes [23,24,25].

While these psychotherapy models are not specifically about theatre workshops, their methodological stance is directly aligned with the present aim: explaining multistability and history dependence as emergent properties of coupled human processes.

### 2.4. Computational and Agent-Based Perspectives on Improvisation and Performance

Research on improvisational performance has also employed computational and agent-based perspectives to formalise how collective structure can arise from moment-to-moment interaction. For example, empirical and computational work in the creativity-and-cognition community has analyzed cognition and interaction in theatrical improvisation as a domain of situated, co-constructed action [26]. Such approaches motivate the use of performance and workshop settings as experimentally controllable microcosms of social coordination, while also highlighting the relative scarcity of computable models that simultaneously capture (i) latent-field-like variables (e.g., trust/safety), (ii) resource constraints (e.g., fatigue/stamina), and (iii) irreversible learning effects at the preference level.

### 2.5. Theatre, Predictive Processing, and Active Inference: Scope of Existing Work

Work connecting theatre and performance to predictive processing and the Free Energy Principle has begun to articulate how engagement, affordances, and spectatorship can be interpreted through prediction-based frameworks. For example, Murphy discusses theatrical experience in terms of predictive engagement and imaginative play within an affordance landscape [27]. These contributions provide useful conceptual bridges, but they do not typically instantiate a multi-agent generative model that can be simulated and evaluated against dynamical signatures (e.g., chaotic itinerancy) in workshop-like group interaction.

In a nearby methodological direction, Active Inference has been used to formalise social structure in terms of shared scripts and action sequences. Albarracin et al. propose a variational formalisation of scripts within Active Inference [28], offering a natural point of contact with role- and script-like regularities in performance settings. However, within the scope of our literature search, we did not identify peer-reviewed work that formulates theatre workshops (or closely related drama-therapeutic group dynamics) as an explicit computational state-space/POMDP model under Active Inference and then systematically evaluates emergent collective dynamics via simulation.

### 2.6. Positioning of the Present Work

Taken together, prior work establishes (a) that collective phase-transition-like behaviour is common in abstract social models and (b) that small-group and psychotherapy contexts can exhibit metastable regimes and discontinuous shifts. It also suggests (c) that improvisational performance is amenable to computational formalisation. The present study contributes a complementary perspective by formulating workshop dynamics as a multi-agent POMDP under Active Inference: chaotic itinerancy—structured switching among multiple metastable collective modes—arises under EFE minimisation with structured state transitions and trust-gated learning, providing a principled account of how groups alternate among transient modes without prescribing collective outcomes.

## 3. Theoretical Framework: Active Inference and Expected Free Energy

### 3.1. Free Energy Principle: Basic Structure

The Free Energy Principle (FEP) describes the behaviour of biological agents as “surprise minimisation” [3]. Da Costa et al. [4] formalized this principle as a normative theory of action selection, demonstrating that Active Inference can be rigorously derived under physically plausible assumptions.

Their core insight is that under the precise agent assumption—macroscopic biological agents respond deterministically to their environment—agent behaviour can be described as Expected Free Energy (EFE) minimisation.

**Definition** **1**  (Precise Agent [4])**.** *A precise agent is one for which the external state s determines both the observation o and the action a:*

(1) $$\begin{array}{cc} P(o|s,e,h_{\leq t}) & =\delta_{f(s)}\left(o\right) \end{array}$$

(2) $$\begin{array}{cc} P(e|s,o,h_{\leq t}) & =\delta_{g(s)}\left(e\right) \end{array}$$

*where δ denotes the Dirac delta function and $h_{\leq t}$ is the agent’s history.*

### 3.2. Expected Free Energy: Definition and Decomposition

According to Da Costa et al. [4], an Active Inference agent is completely characterized by two models:

- Predictive model P(s,o∣a): Joint distribution of external states *s* and observations *o* given action *a*.
- Preference model P(s,o): Distribution over preferred external states and observations.

Action selection is governed by the Expected Free Energy:

(3) $$-\log P\left(a|h\right)=E_{P(s,o|a,h)}\left[\log P(s|a,h)-\log P(s,o|h)\right]$$

The EFE admits two equivalent decompositions:

Risk–Ambiguity Decomposition:

(4) $$-\log P\left(a|h\right)=\underset{\mathrm{Risk}}{\underset{︸}{D_{\mathrm{KL}}\left[P\left(s|a,h\right)∥P\left(s|h\right)\right]}}+\underset{\mathrm{Ambiguity}}{\underset{︸}{E_{P(s|a,h)}\left[H\left[P\left(o|s,h\right)\right]\right]}}$$

- Risk: KL divergence between predicted and preferred external states. Minimizing this reverse KL divergence induces mode-matching behaviour, providing the mathematical foundation for risk-averse decision making.
- Ambiguity: Expected entropy of future observations. High ambiguity indicates uncertainty about what will occur. Ambiguity minimisation drives exploration toward states where observations clearly reveal external states (the streetlight effect).

Extrinsic–Intrinsic Value Decomposition:

(5) $$-\log P\left(a|h\right)\geq -\underset{\mathrm{Extrinsic}\;\mathrm{Value}}{\underset{︸}{E_{P(o|a,h)}\left[\log P\left(o|h\right)\right]}}-\underset{\mathrm{Intrinsic}\;\mathrm{Value}}{\underset{︸}{E_{P(o|a,h)}\left[D_{\mathrm{KL}}\left[P\left(s|o,a,h\right)∥P\left(s|a,h\right)\right]\right]}}$$

- Extrinsic Value: Log-likelihood of preferred observations. It corresponds to reward or utility maximisation, the foundation of reinforcement learning and Bayesian decision theory.
- Intrinsic Value: Information gain from action (mutual information). Provides the mathematical basis for curiosity-driven exploration and active learning, corresponding to Bayesian experimental design.

### 3.3. Three Advantages of Active Inference

Da Costa et al. [4] identify three key advantages of Active Inference:

(a) Principled resolution of exploration–exploitation dilemma: Information gain (intrinsic value) is naturally incorporated into action selection, without requiring separate exploration bonuses.
(b) Explainable action simulation under generative world models: Actions are explained as consequences of beliefs about the world, enabling interpretable agent behaviour.
(c) Universality: Any RL algorithm can be re-described as Active Inference, suggesting AI as a unifying framework for sequential decision making.

### 3.4. Position of This Study

This study applies the theoretical framework to multi-agent collective behaviour, specifically demonstrating that nonlinearity in the predictive model and coupling between state variables can generate chaotic itinerancy—structured switching among multiple metastable collective modes.

One modelling approach for realizing complex collective behaviour is to introduce explicit social priors (social prior distributions) directly into action selection. Here, by incorporating Hill function cooperative effects and inter-variable coupling into the state transition dynamics themselves, we show that chaotic itinerancy emerges from pure EFE minimisation, with trajectories dwelling near multiple quasi-stable “attractor ruins” and switching irregularly among them.

### 3.5. Hill Function Nonlinearity

To model cooperative effects that create phase transitions, we employ the Hill function [29]:

(6) $$H_{n}\left(x;K\right)=\frac{x^{n}}{x^{n}+K^{n}}$$

where *x* is the input variable (e.g., expression rate ${\bar{e}}_{t}$), *n* is the Hill coefficient (cooperativity index), and *K* is the half-saturation constant (critical threshold).

**Definition** **2** (Hill Function)**.**  *The Hill function* $H_{n}\left(x;K\right)$ *has the following properties:*

- $H_{n}\left(0;K\right)=0$,    $H_{n}\left(1;K\right)=\frac{1}{1+K^{n}}$
- $H_{n}\left(K;K\right)=0.5$ *(half-maximum at* x=K*)*
- *Slope at* x=K: ${\left.\frac{dH_{n}}{dx}\right|}_{x=K}=\frac{n}{4K}$
- *Converges to a step function as* n→∞
- n≥4 *is a necessary condition for bistability (a minimal form of multistability) in this model*

Originally developed to model cooperative binding in biochemistry, the Hill function has properties ideal for modelling collective effects:

- Sigmoidal response with threshold behaviour at x=K;
- Sharp transitions for n≥4, enabling bistability (and, more generally, multistable regimes);
- Continuous approximation of threshold dynamics.

Affordance interpretation: Crucially, the Hill function specifies *environmental constraints on action feasibility*—an affordance structure—rather than prescribing preferred outcomes. It encodes the physical and psychosocial conditions under which sustained collective expression becomes viable: when group expression exceeds threshold *K*, the environment *affords* reduced effort costs and enhanced trust gains. This is analogous to how terrain affords walking or obstacles constrain movement—the structure shapes what actions are sustainable, not what actions are desirable. Thus, embedding Hill nonlinearity in state transitions differs fundamentally from introducing outcome-level social priors that directly encode conformity or coordination as preferred observations.

## 4. Model Specification

In what follows, we simulate the behaviour of agents who behave—in relation to each other—in accord with Active Inference. In other words, each agent is equipped with a generative model that allows them to seek evidence for that model by responding to epistemic affordances (i.e., picking policies that maximise expected information gain while complying with the constraints implicit in prior preferences). Crucially, the generative model for each agent is assembled carefully to include natural constraints based upon a theatre workshop model. There are many aspects of this model that we can install by specifying suitable states of agents, some of which are directly observable and some of which have to be inferred. This observation and inference rests upon constraints on the interactions among agents, which are themselves modelled with a network of interactions with local connectivity.

**Figure 1:**
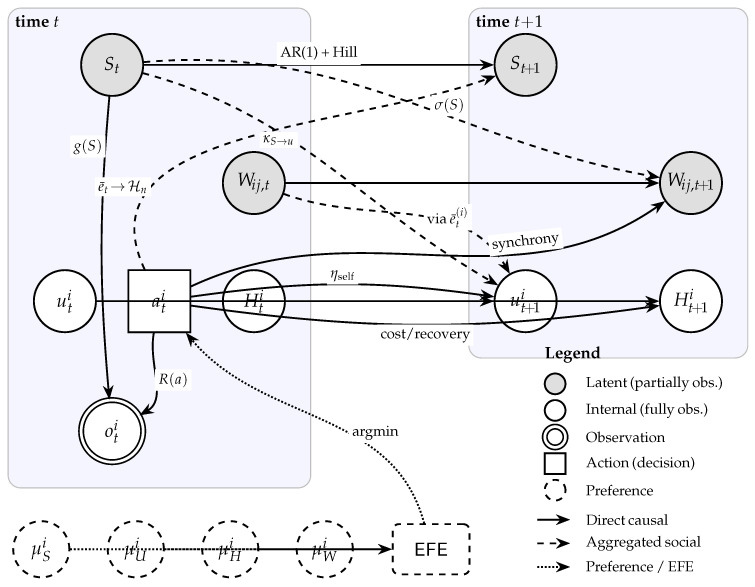
Probabilistic graphical model of the generative model for agent *i*. Shaded nodes are partially observable (latent); white nodes are fully observable or directly controlled. Solid arrows indicate direct causal dependencies in the generative process; dashed arrows indicate aggregated social coupling. Action selection minimizes Expected Free Energy (EFE) computed from this model.

A distinctive feature of this model is that it incorporates Hill function nonlinearity and inter-variable coupling into the predictive model itself, so that agents possess this dynamics as an internal model and reflect it in action selection through the Risk term of EFE. Figure 1 provides a probabilistic graphical model summarizing the generative model for a single agent, showing the causal dependencies among all state variables, observations, actions, and preferences. All simulations were implemented in Python 3.11 using standard numerical libraries.

### 4.1. POMDP Structure

The model is a Partially Observable Markov Decision Process (POMDP) with the following structure:

State Space Classification

For agent *i*, states are classified into three types:

- External states (partially observable): $S_{t}$ (collective trust) and ${\left\{W_{ij,t}\right\}}_{j\in N(i)}$ (interpersonal trust). $S_{t}$ is a “field state” shared by all agents, while $W_{ij,t}$ represents dyadic relational strength. Both are latent and must be inferred from observations.
- Internal states (fully observable): $\left(u_{t}^{i},H_{t}^{i}\right)$—empowerment $u_{t}^{i}$ and stamina $H_{t}^{i}$ are the agent’s own states, which the agent can fully know.

POMDP Components (Table 1)

Observability of State Variables

- $S_{t}$ (collective trust) and $W_{ij,t}$ (interpersonal trust) are latent variables representing the “atmosphere” and “relationship quality,” respectively. These cannot be directly measured and must be inferred from social signals (others’ expression and chat and exercise responses).
- $u_{t}^{i}$ (empowerment) is a subjective feeling of “how empowered one is,” which is fully graspable through self-introspection (We use “empowerment” in its psychological sense—a felt sense of competence, self-determination, and capacity to act effectively [30,31]—rather than in the information-theoretic sense of channel capacity between actions and future states [32]. While both concepts share the intuition that agents seek states enabling effective action, the information-theoretic definition quantifies potential control in bits, whereas our usage captures the subjective experience of self-efficacy that influences action selection through preferences).
- $H_{t}^{i}$ (stamina) is one’s own bodily state, which is fully self-aware.

### 4.2. State Variables

This subsection introduces the set of latent and internal variables required to express the workshop dynamics as a tractable multi-agent POMDP on a network. We distinguish between a shared, partially observable field state $S_{t}$ (collective trust), dyadic relational states $W_{ij,t}$ (interpersonal trust), and agent-specific internal states $\left(u_{t}^{i},H_{t}^{i}\right)$ (empowerment and stamina). Inter-agent coupling is structured by a sparse network, where agents perceive a local trust-weighted average of others’ behaviour. This design choice makes explicit what agents must infer (collective and interpersonal trust) versus what they can access directly (their own empowerment and stamina), which is central for the epistemic term in Expected Free Energy.

Indices

- *i*: Agent (participant) index (i=1,…,N)
- *t*: Discrete time step (t=0,1,2,…,T)
- τ: Relative time within planning horizon $\left(\tau =1,\ldots ,N_{\mathrm{horizon}}\right)$

State Variables

- $S_{t}\in R$: Collective trust (Gaussian latent variable);
- $u_{t}^{i}\in R$: Agent *i*’s empowerment (Gaussian latent variable);
- $H_{t}^{i}\in \left[0,H_{\max}\right]$: Agent *i*’s stamina;
- $W_{ij,t}\in R$: Interpersonal trust between agents *i* and *j* (network edge weight).

Network Structure

Agents are embedded in a sparse undirected network G=(V,E) with adjacency matrix $A_{ij}\in \left\{0,1\right\}$, where $A_{ij}=A_{ji}$. The network is generated as an Erdős–Rényi random graph with connection probability $p=k_{\mathrm{avg}}/\left(N-1\right)$, where $k_{\mathrm{avg}}$ is the target average degree (default: $k_{\mathrm{avg}}=4$). Each edge (i,j)∈E carries a dynamic interpersonal trust weight $W_{ij,t}\in R$ that evolves based on action synchrony (see Section 4.3). The neighborhood of agent *i* is denoted $N\left(i\right)=\left\{j:A_{ij}=1\right\}$.

This network structure serves two purposes:

1. Avoiding complete mean-field approximation: Rather than assuming all agents observe the same global expression rate ${\bar{e}}_{t}$, each agent perceives a *local* expression rate computed over its neighborhood.
2. Maintaining computational tractability: The sparse network topology ($k_{\mathrm{avg}}\ll N$) and local averaging keep inference tractable while introducing heterogeneous social perception.

Gaussian Latent Variable Design

A key design principle is that the primary state variables $S_{t}$ and $u_{t}^{i}$ are modelled as Gaussian latent variables without artificial clipping to [0,1]. This ensures theoretical consistency with the Gaussian assumptions underlying belief updating (Extended Kalman Filter) and information gain computation. Physical effects that require bounded values are obtained through sigmoid transformations:

(7) $$\mathrm{Bounded}\;\mathrm{effect}=\sigma \left(x\right)=\frac{1}{1+e^{-x}}$$

Interpretation of Unbounded States:

- Negative *S* (distrust): Values S<0 represent collective distrust or psychological unsafety. The sigmoid transformation ensures that negative trust does not produce paradoxical amplification effects.
- Negative *u* (disempowerment): Values u<0 represent a sense of powerlessness or learned helplessness.
- Large positive values: Values significantly above 1 represent strong trust or high empowerment, with diminishing marginal physical effects due to sigmoid saturation.

This design choice keeps the model’s state dynamics and belief updating aligned with the Gaussian assumptions used in inference (Extended Kalman Filter) and in the information gain computation. The information gain expression is exact under linear–Gaussian assumptions (see Section 4.8 for the full expression). Accordingly, we keep $S_{t}$ and $u_{t}^{i}$ unconstrained and apply sigmoid transformations only when mapping latent variables to bounded interaction terms, maintaining internal consistency of the computations.

Action Variable

- $a_{t}^{i}\in \left\{0,1,2\right\}$: Agent *i*’s action (0: Rest, 1: Chat and Exercise, 2: Express)
- $e_{t}^{i}\in \left\{0,1\right\}$: Expression indicator, $e_{t}^{i}=1\left[a_{t}^{i}=2\right]$

The three-valued action space distinguishes between:

- Rest (a=0): Passive observation with stamina recovery.
- Chat and Exercise (a=1): Low-cost social interaction that provides *information gain* about interpersonal trust $W_{ij}$ (denoted ${\mathrm{IG}}_{W}$; formally defined in Section 4.8, Equation (54)) and also updates $W_{ij}$ through action synchrony.
- Express (a=2): High-commitment creative expression that consumes stamina, builds collective trust *S* via the Hill-function mechanism, and updates interpersonal trust $W_{ij}$ through action synchrony—but does *not* provide information gain ${\mathrm{IG}}_{W}$ (see Supplementary Material (Section S-X) for sensitivity analysis of this design choice).

Collective Statistics

The model supports two modes of collective coupling:

(a) Global statistics (used for collective trust *S* dynamics):

(8) $$\begin{array}{ccc} {\bar{e}}_{t} & =\frac{1}{N}\sum_{j=1}^{N}e_{t}^{j} & (\mathrm{overall}\;\mathrm{expression}\;\mathrm{rate}) \end{array}$$

(9) $$\begin{array}{ccc} {\bar{c}}_{t} & =\frac{1}{N}\sum_{j=1}^{N}1\left[a_{t}^{j}=1\right] & (\mathrm{overall}\;\mathrm{chat}\;\mathrm{rate}) \end{array}$$

(b) Local statistics (used for empowerment and stamina dynamics):

(10) $${\bar{e}}_{t}^{\left(i\right)}=\frac{\sum_{j\in N(i)}\sigma \left(W_{ij,t}\right)\cdot e_{t}^{j}}{\sum_{j\in N(i)}\sigma \left(W_{ij,t}\right)+\varepsilon }$$

where N(i) is agent *i*’s neighborhood, σ(·) is the sigmoid function, and ε>0 is a small constant for numerical stability.

The local expression rate ${\bar{e}}_{t}^{\left(i\right)}$ is a trust-weighted average over the agent’s neighborhood: neighbors with higher interpersonal trust $W_{ij}$ contribute more to the perceived social context. This replaces the mean-field statistic ${\bar{e}}_{t}^{-i}$ used in earlier mean-field formulations of this model (i.e., the fully-connected, unweighted average over all other agents), enabling heterogeneous social perception while maintaining computational tractability.

Information Asymmetry: Global vs. Local Coupling

The model employs an intentional asymmetry in information scope that reflects the physical structure of theatre workshop settings:

- Global coupling for collective trust *S*: Expressive acts (Express) are publicly visible performances that all participants can observe simultaneously—akin to stage performances visible to the entire room. Accordingly, the collective trust *S* is updated based on the *global* expression rate ${\bar{e}}_{t}$ (Equation (11)).
- Local coupling for empowerment $u^{i}$ and stamina $H^{i}$: In contrast, the psychological impact of others’ expression on one’s own sense of empowerment and fatigue depends on *whom one is attending to*—typically nearby participants with whom one has established interpersonal trust. Hence, empowerment (Equation (12)) and stamina dynamics use the *local* trust-weighted expression rate ${\bar{e}}_{t}^{\left(i\right)}$.
- Local coupling for interpersonal trust $W_{ij}$: Dyadic trust evolves based on pairwise action synchrony between connected agents (Equation (15)).

This two-layer structure—global media (publicly visible expression affecting shared trust climate) combined with local communication (interpersonal relationships affecting individual empowerment)—generalises beyond theatre workshops. Analogous patterns appear in organisational settings (company-wide announcements vs. team-level interactions), social media (viral content vs. direct messaging), and community dynamics (public gatherings vs. neighborhood conversations).

Belief Variables (Perception)

In Active Inference, agents minimise variational free energy to approximate the posterior distribution over hidden states given observations [3,4]. Since collective trust $S_{t}$ and interpersonal trust $W_{ij,t}$ are not directly observable, agent *i* maintains a probabilistic belief Q(s) over them. We employ a Gaussian approximation for these beliefs, parameterized by their sufficient statistics (mean and variance). This belief state constitutes the agent’s “perception” of the social environment and serves as the basis for calculating the epistemic value (information gain) of future actions.

- $m_{S}^{i},v_{S}^{i}\in R\times R_{>0}$: Mean and variance of belief about collective trust *S*
- $m_{W_{ij}}^{i},v_{W_{ij}}^{i}\in R\times R_{>0}$: Mean and variance of belief about interpersonal trust $W_{ij}$

Preference Variables (Goal Directedness)

Active Inference unifies perception and action under the single imperative of minimizing free energy. Goal-directed behaviour arises from the agent’s *prior preferences* over states, denoted as P(s). Action selection minimizes Expected Free Energy (EFE), which includes a “Risk” term defined as the KL divergence between the predicted state distribution and the preferred distribution P(s) [4]. In this model, agents possess preferred setpoints for trust, empowerment, and stamina. Crucially, unlike fixed reward functions in standard RL, these preference parameters (specifically $\mu_{U}^{i}$) can themselves be updated through learning (see Section 4.7).

- $\mu_{S}^{i}\in R$: Preferred collective trust level;
- $\mu_{U}^{i}\in R$: Preferred empowerment level (adaptively learned);
- $\mu_{H}^{i}\in \left[0,H_{\max}\right]$: Preferred stamina level.

### 4.3. State Transition Dynamics

We specify a discrete-time generative process in which (i) collective trust evolves as a leaky (AR(1)) process with a nonlinear, threshold-like social amplification term, (ii) empowerment accumulates through self- and other-driven gains modulated by trust, and (iii) stamina implements a simple resource constraint with recovery and cooperative cost reduction.

Collective Trust:

(11) $$S_{t+1}=a_{S}S_{t}+b_{S}+c_{S}^{\mathrm{Hill}}\cdot H_{n}\left({\bar{e}}_{t};K\right)+c_{S}^{\mathrm{chat}}\cdot {\bar{c}}_{t}+\omega_{t}$$

where $\omega_{t}∼N\left(0,\sigma_{S}^{2}\right)$ is process noise, ${\bar{e}}_{t}$ is the global expression rate (Equation (10)), and ${\bar{c}}_{t}$ is the global chat rate.

Here, $a_{S}S_{t}+b_{S}$ captures gradual decay toward a baseline, the Hill term $c_{S}^{\mathrm{Hill}}H_{n}\left({\bar{e}}_{t};K\right)$ implements a sharp increase in trust once expression becomes sufficiently common (critical threshold *K*), and the linear term $c_{S}^{\mathrm{chat}}{\bar{c}}_{t}$ provides a modest contribution from Chat and Exercise activity.

Parameters:

- $a_{S}$: Decay coefficient ($0<a_{S}<1$);
- $b_{S}$: Baseline constant;
- $c_{S}^{\mathrm{Hill}}$: Hill function effect strength (Express);
- $c_{S}^{\mathrm{chat}}$: Linear effect strength (Chat and Exercise).

The Hill function term captures the nonlinear amplification of trust when group expression exceeds the critical threshold *K*. Conceptually, this corresponds to the idea that a psychologically safe climate can emerge nonlinearly once sufficient interpersonal risk-taking (here, expressions) becomes common [33,34].

Empowerment (with Trust–Empowerment Coupling):

(12) $$\begin{array}{cc} u_{t+1}^{i} & =\alpha_{U}u_{t}^{i}+\eta_{\mathrm{self}}e_{t}^{i}+\eta_{\mathrm{other}}{\bar{e}}_{t}^{\left(i\right)}\left(1+\gamma_{\mathrm{coop}}\cdot \sigma \left(u_{t}^{i}\right)\right)\\ \; & \;+\underset{\mathrm{Trust}–\mathrm{Empowerment}\;\mathrm{Coupling}}{\underset{︸}{\kappa_{S\to u}\cdot \sigma \left(S_{t}\right)\cdot {\bar{e}}_{t}^{\left(i\right)}}}+\xi_{t}^{i} \end{array}$$

where $\xi_{t}^{i}∼N\left(0,\sigma_{U}^{2}\right)$ is process noise, σ(·) is the sigmoid function (Equation (7)), and ${\bar{e}}_{t}^{\left(i\right)}$ is the local expression rate (Equation (10)).

The cooperative factor uses sigmoid-transformed empowerment $\sigma (u_{t}^{i})$ to prevent runaway positive feedback. The trust–empowerment coupling term makes vicarious gains systematically larger when inferred trust is high. Both sigmoid transformations ensure bounded contributions regardless of the magnitude of the underlying Gaussian latent variables.

Parameters:

- $\alpha_{U}$: Decay coefficient ($0<\alpha_{U}<1$);
- $\eta_{\mathrm{self}}$: Empowerment increase from self-expression;
- $\eta_{\mathrm{other}}$: Empowerment increase from others’ expression;
- $\gamma_{\mathrm{coop}}$: Cooperative amplification strength;
- $\kappa_{S\to u}$: Trust–empowerment coupling coefficient.

Theoretical Significance of Trust–Empowerment Coupling:

The underlined term $\kappa_{S\to u}\cdot \sigma \left(S_{t}\right)\cdot {\bar{e}}_{t}^{\left(i\right)}$ represents that when the field trust *S* is high, the positive effect of neighbors’ expression on one’s own empowerment is amplified.

Treatment of Negative Trust: The trust variable $S_{t}$ is modelled as a Gaussian latent variable (Equation (11)) and can take any real value. Physical effects that depend on trust use sigmoid-transformed values, ensuring bounded contributions:

(13) $$S_{\mathrm{eff}}=\sigma \left(S_{t}\right)=\frac{1}{1+e^{-S_{t}}}$$

This design has several advantages:

1. Theoretical consistency: The Kalman filter belief updating and information gain formulas remain exact for the underlying Gaussian process.
2. Asymmetric interpretation: Negative trust (distrust) produces near-zero effective coupling, while positive trust produces positive coupling. This captures the psychological observation that distrust does not symmetrically reverse trust effects—rather, it attenuates or nullifies them.
3. Smooth dynamics: The sigmoid transformation is differentiable everywhere, avoiding discontinuities that could destabilise the dynamics.

This coupling term models several interrelated psychological phenomena. In trusted environments, others’ expressions more readily empower the observer through empathic resonance—witnessing a breakthrough can feel as if it were one’s own—and through imitation and contagion of expressive content [33,34]. Social learning is facilitated: others’ successful experiences transfer more easily to one’s own self-efficacy in safe contexts [31,35], and supportive environments promote internalisation and autonomous motivation [36]. The resulting interaction between *S* and *u* creates positive feedback that stabilizes multistability (here: bistability).

Stamina (with Cooperative Cost Reduction):

(14) $$H_{t+1}^{i}=\left\{\begin{array}{cc} \min(H_{t}^{i}+H_{\mathrm{rec}},H_{\max}) & \mathrm{if}\;a_{t}^{i}=0\;\left(\mathrm{rest}\right)\\ H_{t}^{i}-c_{\mathrm{chat}} & \mathrm{if}\;a_{t}^{i}=1\;(\mathrm{chat}\;\mathrm{and}\;\mathrm{exercise})\\ \max\left(H_{t}^{i}-c_{\exp}\cdot \left(1-c_{H}^{\mathrm{Hill}}\cdot H_{n_{H}}\left({\bar{e}}_{t}^{\left(i\right)};K_{H}\right)\right),0\right) & \mathrm{if}\;a_{t}^{i}=2\;\left(\mathrm{express}\right) \end{array}\right.$$

This piecewise update implements recovery during rest, mild depletion during chat and exercise, and significant depletion during expression, with costs reduced when neighbors are also expressing (cooperative cost reduction via local expression rate ${\bar{e}}_{t}^{\left(i\right)}$). The min/max operators keep stamina within $\left[0,H_{\max}\right]$, representing a hard resource constraint.

Parameters:

- $H_{\max}$: Maximum stamina;
- $H_{\mathrm{rec}}$: Stamina recovery during rest;
- $c_{\mathrm{chat}}$: Chat and Exercise cost (small);
- $c_{\exp}$: Basic expression cost;
- $c_{H}^{\mathrm{Hill}}$: Cooperative cost reduction coefficient;
- $n_{H}$: Hill coefficient (stamina);
- $K_{H}$: Half-saturation constant (stamina).

The factor $\left(1-c_{H}^{\mathrm{Hill}}\cdot H_{n_{H}}\left({\bar{e}}_{t}^{\left(i\right)};K_{H}\right)\right)$ implements cooperative cost reduction: expression costs less when neighbors are also expressing. This positive feedback mechanism is key to enabling multistability. This stylized assumption is consistent with classic findings that the presence of others can systematically modulate effort and performance (social facilitation) [37], and with psychophysical accounts that treat effort as a subjective, reportable quantity (ratings of perceived exertion) [38].

Sociological Justification for the Hill Function

While the Hill function is originally derived from biochemistry (cooperative oxygen binding to hemoglobin), we employ it here as a phenomenological ansatz for threshold-dependent social reinforcement. This aligns with sociological theories of “critical mass” and “complex contagion” [39], which posit that adoption of costly or risky behaviours (like expressive performance) requires social reinforcement from multiple sources exceeding a specific threshold, unlike simple information spreading. The Hill coefficient *n* controls the steepness of this threshold, effectively modelling how strictly the group enforces this critical mass requirement.

From an ecological perspective, the Hill function formalizes an affordance structure: the collective environment either affords or constrains sustained expression depending on whether participation exceeds the critical threshold. This framing clarifies that we are specifying *what the environment makes possible*—a physical and psychosocial constraint—rather than *what agents should prefer*. The collective patterns that emerge are not built into preferences but arise from agents navigating this affordance landscape under EFE minimisation.

Physiological Motivation for Hill-Type Stamina Dynamics

We use a Hill-type nonlinearity as a compact way to represent cooperative, threshold-like changes in endurance costs. The Hill function is classically associated with cooperative oxygen binding and the resulting sigmoidal saturation curve [29], and endurance physiology highlights oxygen transport as a key determinant of fatigue resistance [40]. By analogy, when group expression exceeds a critical threshold, a “collective oxygen supply” effect emerges: mutual entrainment and synchronisation among participants—well documented in sports science and rhythmic coordination [37]—reduces the subjective effort required for sustained expression. Empirically, perceived effort and fatigue exhibit nonlinear dependence on physiological and contextual factors [38]. In this model the Hill term is not a literal haemoglobin model; it is an abstract mechanism by which supportive collective conditions can reduce the effective cost of expression once participation exceeds a critical level.

Interpersonal Trust Dynamics

In addition to the collective trust $S_{t}$, agents maintain dyadic interpersonal trust weights $W_{ij,t}$ on network edges. These weights evolve based on action synchrony—whether paired agents take coordinated actions:

(15) $$W_{ij,t+1}=a_{W}W_{ij,t}+b_{W}+c_{W}\cdot \phi_{\mathrm{base}}\left(a_{t}^{i},a_{t}^{j}\right)\cdot \sigma \left(S_{t}\right)+\nu_{ij,t}$$

where $\nu_{ij,t}∼N\left(0,\sigma_{W}^{2}\right)$ is process noise and $\phi_{\mathrm{base}}\left(a^{i},a^{j}\right)$ is the synchrony effect function (Table 2):

Interpretation:

- Express–Express synchrony (ϕ=+1.0): Joint creative expression strongly builds interpersonal trust.
- Chat and Exercise synchrony (ϕ=+0.5): Mutual conversation and exercise moderately builds trust.
- Asynchrony (ϕ<0): Mismatched actions (one expresses while the other rests) erode trust.

The trust modulation $\sigma (S_{t})$ ensures that interpersonal trust builds more readily in high collective trust environments. Since $W_{ij}=W_{ji}$ (undirected network), both agents experience the same trust update.

Key distinction: All actions (Rest, Chat and Exercise, Express) affect the *true dynamics* of $W_{ij}$ through the synchrony matrix $\phi_{\mathrm{base}}$. However, only Chat and Exercise provides *information gain* about $W_{ij}$ (see Equation (54)). This separation means that Express can build interpersonal trust through joint creative activity, but agents cannot directly observe this trust-building; they can only learn about interpersonal trust through Chat and Exercise.

Network Parameters (Table 3):

For belief updating over $W_{ij}$, agents use variational message passing (VMP) with Jaakkola bounds to handle the sigmoid nonlinearity in Equation (10). Details are provided in Supplementary Material (Section S-XIII).

### 4.4. Predictive and Preference Models

Active Inference agents are completely characterized by two models:

(1) Predictive Model P(s,o∣a)

Prediction of how external states and observations evolve under action a∈{0,1,2}. In this model:

(16) $$\begin{array}{cc} P(S_{t+1}|S_{t},{\bar{e}}_{t},{\bar{c}}_{t})= & N(a_{S}S_{t}+b_{S}\\ \; & +c_{S}^{\mathrm{Hill}}H_{n}\left({\bar{e}}_{t};K\right)+c_{S}^{\mathrm{chat}}{\bar{c}}_{t},\;\sigma_{S}^{2}) \end{array}$$

(17) $$\begin{array}{cc} P(o_{t}^{i}|S_{t},a_{t}^{i}) & =N\left(g\left(S_{t}\right),\;R\left(a_{t}^{i}\right)\right) \end{array}$$

where g(·) is the observation function (mapping to others’ expression rate).

(2) Preference Model P(s,o)

Distribution over preferred external states and observations. This model has preferences for all three state variables:

(18) $$\begin{array}{cc} \; & P_{\mathrm{pref}}\left(S,u^{i},H^{i}\right)\propto \\ \; & \;\exp\;\left(-\frac{1}{2}\left[w_{S}{\left(S-\mu_{S}^{i}\right)}^{2}+w_{U}{\left(u^{i}-\mu_{U}^{i}\right)}^{2}\right.\right.\\ \; & \;\left.\left.+w_{H}{\left(H^{i}-\mu_{H}^{i}\right)}^{2}\right]\right) \end{array}$$

where:

- Preference for *S*: Preferred trust $\mu_{S}^{i}$ (belief-based since partially observable)
- Preference for $u^{i}$: Preferred empowerment $\mu_{U}^{i}$ (fully observable)
- Preference for $H^{i}$: Preferred stamina $\mu_{H}^{i}$ (fully observable)

### 4.5. Observation Model

Agents observe others’ expression rate with noise:

(19) $$o_{t}^{i}={\bar{e}}_{t}^{\left(i\right)}+\epsilon_{t}^{i},\;\epsilon_{t}^{i}∼N\left(0,R\left(a_{t}^{i}\right)\right)$$

where ${\bar{e}}_{t}^{\left(i\right)}$ is the local expression rate (Equation (10)) and the observation noise depends on action:

(20) $$R\left(a\right)=\left\{\begin{array}{cc} R_{\mathrm{rest}} & a=0\\ R_{\mathrm{chat}} & a=1\\ R_{\mathrm{express}} & a=2 \end{array}\right.\;\left(R_{\mathrm{express}}<R_{\mathrm{chat}}<R_{\mathrm{rest}}\right)$$

This active sensing property means that more engaged actions (Express > Chat and Exercise > Rest) improve observation precision, linking action to information gain. Express provides the highest precision for observing collective trust *S*, while Chat and Exercise provides moderate precision but uniquely enables information gain about interpersonal trust $W_{ij}$.

### 4.6. Belief Update via Extended Kalman Filter

In this work, we implement the minimisation of variational free energy using the functional form of an extended Kalman filter. For the partially observable trust *S*, agents maintain Gaussian beliefs $Q\left(S_{t}\right)=N\left(m_{S}^{i},v_{S}^{i}\right)$. Upon receiving observation $o_{t}^{i}$, beliefs are updated via extended Kalman filter:

Prediction Step:

(21) $$\begin{array}{cc} m_{S}^{i,\mathrm{prior}} & =a_{S}m_{S}^{i}+b_{S}+c_{S}^{\mathrm{Hill}}\cdot H_{n}\left(\hat{e};K\right) \end{array}$$

(22) $$\begin{array}{cc} v_{S}^{i,\mathrm{prior}} & =a_{S}^{2}v_{S}^{i}+\sigma_{S}^{2} \end{array}$$

Update Step:

(23) $$\begin{array}{cc} K_{t}^{i} & =\frac{Hv_{S}^{i,\mathrm{prior}}}{H^{2}v_{S}^{i,\mathrm{prior}}+R\left(a_{t}^{i}\right)} \end{array}$$

(24) $$\begin{array}{cc} m_{S}^{i,\mathrm{post}} & =m_{S}^{i,\mathrm{prior}}+K_{t}^{i}\cdot \left(o_{t}^{i}-Hm_{S}^{i,\mathrm{prior}}\right) \end{array}$$

(25) $$\begin{array}{cc} v_{S}^{i,\mathrm{post}} & =\left(1-K_{t}^{i}H\right)v_{S}^{i,\mathrm{prior}} \end{array}$$

where ${H\equiv \partial g/\partial S|}_{S=m_{S}^{i}}$ is the observation Jacobian (i.e., the linearized gradient of the observation function g(·) evaluated at the current belief mean; not to be confused with stamina $H_{t}^{i}$). In the current implementation H=1 because the observation function is approximately linear (see Equation (53)).

### 4.7. Precision-Gated Preference Learning

A distinctive feature of our model is that preferences can update dynamically based on experience. This implements and extends Preferential Inference [4] to include adaptive preference parameter learning.

#### 4.7.1. Theoretical Foundation: Hierarchical Bayesian Model

We formulate preference learning as variational inference over a hierarchical generative model. Rather than treating the preference parameter $\mu_{U}^{i}$ (the empowerment setpoint) as updated by ad hoc threshold rules, we hierarchically embed it as a latent hyperparameter subject to precision-weighted Bayesian updating.

Hierarchical Generative Model:

(26) $$\begin{array}{cc} \; & \mathrm{Level}\;1:\;\mu_{U,t+1}^{i}=\mu_{U,t}^{i}+\zeta_{t}^{i},\;\zeta_{t}^{i}∼N\left(0,\sigma_{\mu }^{2}\right) \end{array}$$

(27) $$\begin{array}{cc} \; & \mathrm{Level}\;2:\;{\tilde{u}}_{t}^{i}|\mu_{U}^{i}∼N\left(\mu_{U}^{i},\sigma_{\tilde{u}}^{2}\left(S_{t}\right)\right) \end{array}$$

where ${\tilde{u}}_{t}^{i}$ is a “learning signal” derived from experienced empowerment, and $\sigma_{\tilde{u}}^{2}\left(S_{t}\right)$ is a trust-dependent observation variance (inverse precision).

#### 4.7.2. Precision Modulation

The key mechanism is precision modulation: the precision (inverse variance) of the learning signal depends on current trust level $S_{t}$.

(28) $$\Pi_{\tilde{u}}\left(S_{t}\right)=\sigma_{\tilde{u}}^{-2}\left(S_{t}\right)=\Pi_{\min}+\left(\Pi_{\max}-\Pi_{\min}\right)\cdot \sigma \left(\lambda (S_{t}-\theta_{S})\right)$$

where σ(·) is the logistic function, and λ controls the sharpness of the transition.

Interpretation: When trust is high ($S_{t}>\theta_{S}$), the precision $\Pi_{\tilde{u}}$ approaches $\Pi_{\max}$, meaning observed empowerment is treated as a reliable signal for updating preferences. When trust is low, precision approaches $\Pi_{\min}$, and the same observation has minimal influence on preference learning. This implements the psychological intuition that high empowerment experiences in untrusted environments are likely to be attributed to external factors (“just happened to work out”) rather than internalized as genuine capability.

Theatre workshop interpretation: A participant who spontaneously leads a scene during an early, awkward session may dismiss the experience as a fluke. The same experience in a cohesive, supportive group is more likely to be internalized as “I can do this”—updating the agent’s preference setpoint upward.

#### 4.7.3. Variational Update (Kalman Form)

Given the hierarchical model, the variational (or Kalman) update for the preference mean is:

(29) $$\mu_{U}^{i,\mathrm{post}}=\mu_{U}^{i,\mathrm{prior}}+K_{t}^{i}\cdot E\left[z_{t}^{i}\right]\cdot \left({\tilde{u}}_{t}^{i}-\mu_{U}^{i,\mathrm{prior}}\right)$$

where the Kalman gain $K_{t}^{i}$ is determined by the precision ratio:

(30) $$K_{t}^{i}=\frac{v_{\mu }\cdot \Pi_{\tilde{u}}\left(S_{t}\right)}{v_{\mu }\cdot \Pi_{\tilde{u}}\left(S_{t}\right)+1}$$

and $v_{\mu }$ is the prior variance of the preference hyperparameter.

#### 4.7.4. Empowerment Overshoots Latent Variable

To implement the empirical observation that comfort zones “expand but do not easily contract,” we introduce a latent empowerment overshoots indicator $z_{t}^{i}\in \left\{0,1\right\}$:

(31) $$E\left[z_{t}^{i}\right]=\sigma \left(\lambda_{z}\left(u_{t}^{i}-\mu_{U}^{i}-\theta_{\mathrm{gap}}\right)\right)$$

When $z_{t}^{i}=1$, the empowerment experience is treated as a “mastery” signal that updates preferences. When $z_{t}^{i}=0$, the experience is attributed to external factors and does not update preferences.

Theatre workshop interpretation: The empowerment overshoots correspond to Grotowski’s “removal of habitual defenses” [7]—a moment when the participant breaks through their usual shell and acts beyond their prior comfort zone ($u>\mu_{U}+\theta_{\mathrm{gap}}$). Only such threshold-crossing experiences expand the preference setpoint. Incremental improvements within the current comfort zone do not trigger this update, reflecting the experiential distinction between routine practice and transformative breakthrough.

Unidirectional expansion: By setting $E[z_{t}^{i}]=0$ when $u_{t}^{i}<\mu_{U}^{i}$, we ensure that preferences only update upward, never downward. This implements the “ratchet effect” whereby comfort zones expand irreversibly.

Combined Update: The effective update weight is $K_{t}^{i}\cdot E\left[z_{t}^{i}\right]$, combining:

- Precision gating ($K_{t}^{i}$): How much to trust the observation (trust-dependent)
- Mastery gating ($E[z_{t}^{i}]$):
  Whether the experience qualifies as genuine growth (gap-dependent) (Table 4).

#### 4.7.5. Relationship to Prior Work

This formulation connects to several strands of Active Inference literature:

- Preferential Inference: Da Costa et al. [4] define preferential inference as approximating $P(s,o|h_{\leq t})$ with $Q(s,o|h_{\leq t})$, where the preference model depends on history. Our extension treats preference *parameters* (not just the conditional distribution) as subject to inference.
- Precision as confidence: The interpretation of precision as “confidence” or “reliability” is standard in Active Inference [41]. Here, we apply this principle to preference learning rather than just observation.
- Empirical priors: Friston’s formulation of empirical priors [3] allows priors to depend on random variables learned from experience. Our preference hyperparameter $\mu_{U}$ functions as such an empirical prior.

### 4.8. EFE Computation and Action Selection

#### 4.8.1. State Variable Prediction

Prediction of each state variable τ steps ahead. Note that the agent’s predictive model is a simplification of the true environmental dynamics. In particular, the trust–empowerment coupling term $\kappa_{S\to u}\cdot \sigma \left(S_{t}\right)\cdot {\bar{e}}_{t}^{\left(i\right)}$ that appears in the environmental state transitions (Equation (12)) is omitted from the agent’s internal predictive model. This design choice reflects three considerations:

1. Partial observability of trust: Since $S_{t}$ is only partially observable, the agent cannot condition predictions on its true value. Substituting the belief mean $m_{S}^{i}$ would add uncertainty and bookkeeping to multi-step predictions.
2. Complexity of social interaction: Predicting co-evolving others’ behaviour and collective trust is cognitively demanding. Omitting this interaction term is consistent with bounded rationality: agents deploy a simpler internal model that respects realistic limits on prospective social prediction [42].
3. Conservative prediction: Without the trust-mediated bonus, predicted empowerment gains are conservative; any such gains realized in the environment appear as positive surprise.

This *model mismatch* is consistent with bounded rationality [42] and does not prevent effective action selection.

(a) Collective Trust *S* (Partially Observable → Expressed as Belief)

(32) $$Q\left(S_{t+\tau }|a,h_{\leq t}^{i}\right)=N\left(m_{t+\tau }^{a},v_{t+\tau }^{a}\right)$$

where mean and variance are computed recursively:

(33) $$\begin{array}{cc} m_{t+\tau }^{a} & =a_{S}m_{t+\tau -1}^{a}+b_{S}+c_{S}^{\mathrm{Hill}}H_{n}\left(\hat{e};K\right) \end{array}$$

(34) $$\begin{array}{cc} v_{t+\tau }^{a} & =a_{S}^{2}v_{t+\tau -1}^{a}+\sigma_{S}^{2} \end{array}$$

where $\hat{e}=1\left[a=2\right]$ is the expression indicator for action *a*.

(b) Empowerment *u* (Fully Observable → Point Estimate)

The agent’s predictive model for empowerment omits the trust–empowerment coupling:

(35) $$\begin{array}{cc} {\hat{u}}_{t+\tau }^{i,a} & =\alpha_{U}{\hat{u}}_{t+\tau -1}^{i,a}+\eta_{\mathrm{self}}\cdot 1\left[a=2\right]\\ \; & \;+\eta_{\mathrm{other}}\cdot {\hat{\bar{e}}}^{-i}\cdot \left(1+\gamma_{\mathrm{coop}}\cdot \sigma \left({\hat{u}}_{t+\tau -1}^{i,a}\right)\right) \end{array}$$

where 1[a=2] indicates that only Express actions contribute self-expression gains.

(c) Stamina *H* (Fully Observable → Point Estimate)

(36) $${\hat{H}}_{t+\tau }^{i,a}=\left\{\begin{array}{cc} \min\left({\hat{H}}_{t+\tau -1}^{i,a}+H_{\mathrm{rec}},H_{\max}\right)\; & \mathrm{if}\;a=0\;\left(\mathrm{Rest}\right)\\ {\hat{H}}_{t+\tau -1}^{i,a}-c_{\mathrm{chat}}\; & \mathrm{if}\;a=1\;(\mathrm{Chat}\;\mathrm{and}\;\mathrm{Exercise})\\ \max\left({\hat{H}}_{t+\tau -1}^{i,a}-c_{\exp}\cdot \left(1-c_{H}^{\mathrm{Hill}}\cdot H_{n_{H}}\left({\hat{\bar{e}}}^{-i};K_{H}\right)\right),0\right)\; & \mathrm{if}\;a=2\;\left(\mathrm{Express}\right) \end{array}\right.$$

#### 4.8.2. Risk Computation (All Four State Variables)

Risk is the KL divergence between predicted states and preferred states. Assuming independence of each state variable:

(37) $$\begin{array}{cc} {\mathrm{Risk}}_{t+\tau }\left(a\right) & ={\mathrm{Risk}}_{t+\tau }^{S}\left(a\right)+{\mathrm{Risk}}_{t+\tau }^{u}\left(a\right)\\ \; & \;+{\mathrm{Risk}}_{t+\tau }^{H}\left(a\right)+{\mathrm{Risk}}_{t+\tau }^{W}\left(a\right) \end{array}$$

(a) Risk for Trust (KL divergence between Gaussian distributions) We quantify pragmatic deviation from preferences by the Kullback–Leibler divergence between the agent’s predicted belief over $S_{t+\tau }$ and its preferred distribution. Let

(38) $$q\left(S_{t+\tau }\right)=N\left(m_{t+\tau }^{a},v_{t+\tau }^{a}\right),\;p\left(S_{t+\tau }\right)=N\left(\mu_{S}^{i},\sigma_{\mathrm{pref},S}^{2}\right).$$

Then ${\mathrm{Risk}}_{t+\tau }^{S}\left(a\right)=D_{\mathrm{KL}}\left[q\;∥\;p\right]$ admits a closed form. Starting from $D_{\mathrm{KL}}\left[q\;∥\;p\right]=E_{q}\left[\log q-\log p\right]$ and using the standard Gaussian identities for quadratic expectations,

(39) $$E_{q}\left[{\left(S-\mu_{S}^{i}\right)}^{2}\right]={\left(m_{t+\tau }^{a}-\mu_{S}^{i}\right)}^{2}+v_{t+\tau }^{a},$$

we obtain the expression below.

(40) $$\begin{array}{cc} {\mathrm{Risk}}_{t+\tau }^{S}\left(a\right) & =D_{\mathrm{KL}}\;\left[N\left(m_{t+\tau }^{a},v_{t+\tau }^{a}\right)\;∥\;N\left(\mu_{S}^{i},\sigma_{\mathrm{pref},S}^{2}\right)\right]\\ \; & =\frac{1}{2}\left[\frac{v_{t+\tau }^{a}}{\sigma_{\mathrm{pref},S}^{2}}+\frac{{\left(m_{t+\tau }^{a}-\mu_{S}^{i}\right)}^{2}}{\sigma_{\mathrm{pref},S}^{2}}-1-\log\frac{v_{t+\tau }^{a}}{\sigma_{\mathrm{pref},S}^{2}}\right] \end{array}$$

*Implementation note:* For computational efficiency, the implementation uses a quadratic approximation that omits the constant and logarithmic terms:

(41) $${\mathrm{Risk}}_{t+\tau }^{S}\left(a\right)\approx w_{S}\left[{\left(m_{t+\tau }^{a}-\mu_{S}^{i}\right)}^{2}+v_{t+\tau }^{a}\right]$$

where $w_{S}$ is a weighting coefficient. This approximation is motivated by retaining only the terms that vary with action-dependent predictions: the mean-deviation penalty ${\left(m_{t+\tau }^{a}-\mu_{S}^{i}\right)}^{2}$ and the uncertainty penalty $v_{t+\tau }^{a}$. The remaining terms (−1 and the logarithmic ratio) are either constant under fixed preference variance or can introduce numerical instability when $v_{t+\tau }^{a}$ becomes very small.

(b) Risk for Empowerment

Empowerment *u* is treated as (effectively) fully observable to the agent, so the pragmatic term does not require a belief distribution with substantial uncertainty. Formally, one can recover the squared-deviation form as a small-variance limit of a KL divergence. For example, represent the predicted state by a narrow Gaussian $q\left(u_{t+\tau }\right)=N\left({\hat{u}}_{t+\tau }^{i,a},\epsilon \right)$ with ϵ→0, and the preference by $p\left(u_{t+\tau }\right)=N\left(\mu_{U}^{i},\sigma_{\mathrm{pref},U}^{2}\right)$. Then

(42) $$D_{\mathrm{KL}}\left[q\;∥\;p\right]=\frac{1}{2}\left[\frac{{\left({\hat{u}}_{t+\tau }^{i,a}-\mu_{U}^{i}\right)}^{2}+\epsilon }{\sigma_{\mathrm{pref},U}^{2}}-1-\log\frac{\epsilon }{\sigma_{\mathrm{pref},U}^{2}}\right],$$

so, up to constants and a rescaling, Risk reduces to a squared deviation from the preferred empowerment level. Accordingly, we use:

(43) $${\mathrm{Risk}}_{t+\tau }^{u}\left(a\right)=\frac{w_{U}}{2}{\left({\hat{u}}_{t+\tau }^{i,a}-\mu_{U}^{i}\right)}^{2}$$

*Implementation note:* The implementation includes a predictive variance term to account for future uncertainty in *u*:

(44) $${\mathrm{Risk}}_{t+\tau }^{u}\left(a\right)\approx w_{U}\left[{\left({\hat{u}}_{t+\tau }^{i,a}-\mu_{U}^{i}\right)}^{2}+v_{u,t+\tau }^{\mathrm{prior}}\right]$$

where $v_{u,t+\tau }^{\mathrm{prior}}$ accumulates process noise over the prediction horizon.

(c) Risk for Stamina

(45) $${\mathrm{Risk}}_{t+\tau }^{H}\left(a\right)=\frac{w_{H}}{2}{\left({\hat{H}}_{t+\tau }^{i,a}-\mu_{H}^{i}\right)}^{2}$$

This form corresponds to the “fully observed” or “zero-variance” limit: if one were to represent stamina with a narrow Gaussian belief $N({\hat{H}}_{t+\tau }^{i,a},\epsilon )$ and take ϵ→0, the KL-based risk reduces (up to constants) to a squared deviation from the preferred level $\mu_{H}^{i}$.

*Implementation note:* Similarly to empowerment, the implementation can include a predictive variance term for *H* to penalise uncertainty accumulation over the horizon:

(46) $${\mathrm{Risk}}_{t+\tau }^{H}\left(a\right)\approx w_{H}\left[{\left({\hat{H}}_{t+\tau }^{i,a}-\mu_{H}^{i}\right)}^{2}+v_{H,t+\tau }^{\mathrm{prior}}\right],$$

where $v_{H,t+\tau }^{\mathrm{prior}}$ summarizes accumulated process noise in the stamina prediction.

(d) Risk for Interpersonal Trust (aggregated over neighborhood)

The Risk for interpersonal trust is computed using the aggregated interpersonal trust ${\bar{W}}^{\left(i\right)}$ and its uncertainty ${\bar{v}}_{W}^{\left(i\right)}$:

(47) $$\begin{array}{cc} {\bar{W}}_{t}^{\left(i\right)} & =\frac{1}{\left|N(i)\right|}\sum_{j\in N(i)}m_{W_{ij},t} \end{array}$$

(48) $$\begin{array}{cc} {\bar{v}}_{W,t}^{\left(i\right)} & =\frac{1}{{\left|N\left(i\right)\right|}^{2}}\sum_{j\in N(i)}v_{W_{ij},t} \end{array}$$

where $m_{W_{ij}}$ and $v_{W_{ij}}$ are the mean and variance of agent *i*’s belief about $W_{ij}$.

The variance expression follows from a standard propagation-of-uncertainty argument. If the dyadic beliefs are treated as conditionally independent Gaussians $W_{ij}∼N\left(m_{W_{ij},t},v_{W_{ij},t}\right)$ given $h_{\leq t}^{i}$, then their average

(49) $${\bar{W}}_{t}^{\left(i\right)}=\frac{1}{\left|N(i)\right|}\sum_{j\in N(i)}W_{ij,t}$$

is also Gaussian with

(50) $$\begin{array}{cc} \mathrm{Var}({\bar{W}}_{t}^{\left(i\right)})\; & =\frac{1}{{\left|N\left(i\right)\right|}^{2}}\sum_{j\in N(i)}\mathrm{Var}\left(W_{ij,t}\right)\\ \; & =\frac{1}{{\left|N\left(i\right)\right|}^{2}}\sum_{j\in N(i)}v_{W_{ij},t}. \end{array}$$

which yields ${\bar{v}}_{W,t}^{\left(i\right)}$.

The Risk penalizes deviation from a neutral interpersonal trust preference $\mu_{W}$:

(51) $${\mathrm{Risk}}_{t+\tau }^{W}\left(a\right)=w_{W}\left[{\left({\bar{W}}_{t+\tau }^{\left(i\right)}-\mu_{W}\right)}^{2}+{\bar{v}}_{W,t+\tau }^{\left(i\right)}\right]$$

This quadratic form can be viewed as the same approximation used for collective trust (Equation (41)), applied to the aggregated latent variable ${\bar{W}}^{\left(i\right)}$. Intuitively, it encourages neighborhood-averaged trust to stay near a “neutral” target while discouraging policies that leave large uncertainty in dyadic relations.

Parameters:

- $w_{W}=0.5$: Interpersonal trust weight in EFE
- $\mu_{W}=0.5$: Preferred interpersonal trust (neutral)

#### 4.8.3. Information Gain Computation (Computed for Latent *S* and *W*)

In our formulation, the information-gain (epistemic) term is computed for partially observable latent variables: the collective trust *S* and the interpersonal trust $W_{ij}$. Because (u,H) are directly observed at each time step in the model, they yield *zero observation-based* information gain under the assumed observation model. Importantly, this does *not* mean that epistemic value is irrelevant for predicting future internal dynamics: since the transitions of *u* and *H* depend (directly or indirectly) on *S* and *W*, reducing uncertainty about these latent variables also reduces uncertainty about *futureu* and *H* trajectories through the predictive model.

Under the linear Gaussian model, information gain for *S* can be computed exactly:

(52) $$I_{t+\tau }^{S}\left(a\right)=I\left(S_{t+\tau };o_{t+\tau }^{i}|a\right)=\frac{1}{2}\log\left(1+\frac{H^{2}v_{t+\tau -1}^{a}}{R(a)}\right)$$

where *H* is the observation matrix (linearized gradient of the observation function).

*Implementation note:* The implementation assumes a direct observation model with H=1, yielding:

(53) $$I_{t+\tau }^{S}\left(a\right)\approx \frac{1}{2}\log\left(1+\frac{v_{t+\tau -1}^{a}}{R(a)}\right)$$

This simplification is appropriate when the observation function is approximately linear near the current belief mean.

Important Design: Since R(a=2)<R(a=0) (expressing reduces observation noise), expression action increases information gain for *S*.

Information Gain for Interpersonal Trust *W* (Chat and Exercise only)

A key design feature of the network model is that information gain about interpersonal trust $W_{ij}$ is obtained exclusively through Chat and Exercise actions. This separation implements the distinction between:

- Curiosity-driven social exploration (Chat and Exercise): Low-commitment interaction that reveals information about interpersonal relationships.
- Expression-driven coordination (Express): High-commitment action focused on building collective trust and empowerment.

The information gain for interpersonal trust is:

(54) $$I_{t+\tau }^{W}\left(a\right)=\left\{\begin{array}{cc} \frac{1}{2}\log\left(1+\frac{{\bar{v}}_{W,t+\tau }^{\left(i\right)}\;\left|N\left(i\right)\right|}{\sigma_{W}^{2}}\right) & a=1\\ 0 & \mathrm{otherwise} \end{array}\right.$$

where ${\bar{v}}_{W,t+\tau }^{\left(i\right)}$ is the average variance of beliefs about neighbors’ trust. Note that the term is scaled by |N(i)|, reflecting that Chat and Exercise provides information about *all* neighbors simultaneously. While this implies that agents with higher degrees (more connections) can potentially gain more total information, this bias is consistent with the social reality that well-connected individuals have more to learn from social interaction. In our simulations using Erdős-Rényi graphs, the degree distribution is relatively homogeneous, minimizing the impact of this potential bias.

Interpretation: Chat and Exercise provides observations that reduce uncertainty about neighbors’ trustworthiness (${\mathrm{IG}}_{W}>0$), while Rest and Express do not provide such information (${\mathrm{IG}}_{W}=0$). However, *both Chat and Exercise and Express update the true value of $W_{ij}$* through action synchrony (Equation (15)): Express–Express synchrony strongly builds interpersonal trust (ϕ=+1.0), while Chat and Exercise synchrony moderately builds trust (ϕ=+0.5).

This creates a three-way tradeoff in action selection:

- Rest: Stamina recovery, minimal information gain, weak synchrony effect on $W_{ij}$.
- Chat and Exercise: Moderate stamina cost, ${\mathrm{IG}}_{W}>0$ (curiosity-driven exploration), moderate synchrony effect on $W_{ij}$.
- Express: High stamina cost, ${\mathrm{IG}}_{S}>0$ (collective trust observation), empowerment gain, *strong* synchrony effect on $W_{ij}$ but ${\mathrm{IG}}_{W}=0$.

Theatre workshop interpretation: Joint improvisation (Express) builds solidarity through shared creative activity—interpersonal trust $W_{ij}$ genuinely increases—but participants are absorbed in the performance itself, not attending to how others perceive them. Only through conversation and group exercises (Chat and Exercise) can an agent recognize, “Ah, that person trusts me.” This asymmetry between *building* trust and *knowing* about trust captures a realistic feature of experiential learning.

#### 4.8.4. Differing Roles of Fully and Partially Observable Variables

This structure implies (Table 5):

- For *S*: Both information gain (via Express) and risk contribute.
- For $W_{ij}$: Information gain (via Chat and Exercise) and risk contribute; Chat and Exercise enables curiosity-driven social exploration.
- For u,H: Risk contributes directly; epistemic drive enters *indirectly* via reduced uncertainty in *S* and *W* which improves multi-step prediction of (u,H).

#### 4.8.5. *N*-Step Expected Free Energy

The *N*-step EFE is defined as:

(55) $$\begin{array}{cc} G^{\left(N\right)}\left(a\right)\; & =\sum_{\tau =1}^{N}\left[\underset{\mathrm{Risk}\;(4\;\mathrm{vars})}{\underset{︸}{{\mathrm{Risk}}_{t+\tau }^{S}+{\mathrm{Risk}}_{t+\tau }^{u}+{\mathrm{Risk}}_{t+\tau }^{H}+{\mathrm{Risk}}_{t+\tau }^{W}}}\right]\\ \; & \;-\sum_{\tau =1}^{N}\underset{\mathrm{Info}\;\mathrm{Gain}}{\underset{︸}{\left(I_{t+\tau }^{S}+I_{t+\tau }^{W}\right)}} \end{array}$$

*Implementation note:* When computing the *N*-step EFE, the variance at step τ depends on expected observations at earlier steps. The exact update requires a Kalman update at each step; for computational efficiency, the implementation uses an exponential approximation:

(56) $$v_{t+\tau }^{\mathrm{post}}\approx v_{t+\tau }^{\mathrm{prior}}\cdot \exp\left(-2\cdot I_{t+\tau }\left(a\right)\right)$$

This approximation exploits the relationship between information gain and variance reduction in Gaussian models.

#### 4.8.6. Action Selection Rule

Action selection is based purely on EFE with softmax policy over the three-valued action space:

(57) $$P\left(a_{t}^{i}=a\right)=\frac{\exp(-\beta_{\mathrm{action}}\cdot G^{\left(N\right)}\left(a\right))}{\sum_{a^{\prime }\in \left\{0,1,2\right\}}\exp\left(-\beta_{\mathrm{action}}\cdot G^{\left(N\right)}\left(a^{\prime }\right)\right)}$$

This is softmax action selection, where $\beta_{\mathrm{action}}$ is the inverse temperature parameter controlling the exploration–exploitation tradeoff. Higher $\beta_{\mathrm{action}}$ leads to more deterministic selection of the action with lowest EFE.

### 4.9. Propagation of Nonlinear Effects to Risk

Although the nonlinearities in this model are implemented in the state transitions (Hill-type collective effects and trust-gated learning), their behavioural consequence is expressed through the Risk component of EFE because Risk scores the mismatch between predicted trajectories and preferred setpoints. In particular, the empowerment Risk (Equation for ${\mathrm{Risk}}^{u}$) depends on the predicted future empowerment ${\hat{u}}_{t+\tau }^{i,a}$ under each candidate action. When collective trust *S* is high in the environment, the true dynamics provide a stronger vicarious gain channel through the trust–empowerment coupling term in Equation (12). As a result, policies that include Express (directly or indirectly by inducing others’ expression) tend to yield higher realized empowerment than in low-trust regimes. Even though the agent’s internal predictive model is intentionally simplified (Section 4.8), this regime dependence still feeds back into planning via the accumulated prediction error and the subsequent updates of belief and preference parameters.

Comfort-zone expansion then changes the geometry of the same Risk term. Preference learning updates the empowerment setpoint $\mu_{U}^{i}$ upward when a high empowerment experience is encountered under sufficient inferred trust (Section 4.7). Because ${\mathrm{Risk}}_{t+\tau }^{u}\left(a\right)$ penalizes deviations of ${\hat{u}}_{t+\tau }^{i,a}$ from $\mu_{U}^{i}$, an increase in $\mu_{U}^{i}$ immediately reweights the EFE landscape: trajectories that would previously be “too high” in *u* become less risky, making sustained high-expression/high-empowerment regimes more self-consistent under EFE minimisation. Conversely, in low-trust phases the precision-gating mechanism suppresses updates of $\mu_{U}^{i}$, so the agent retains a lower setpoint and high-*u* trajectories remain costly.

The interaction of these two mechanisms strengthens a key signature of chaotic itinerancy: prolonged residence near multiple quasi-stable (metastable) regimes and irregular transitions among them. High-trust episodes make it easier for expression to generate large empowerment excursions; if such excursions are consolidated into a higher $\mu_{U}^{i}$, future expressive policies become less penalized by Risk and therefore more likely to be selected, stabilizing a high-activity mode. If the system remains in a low-trust region long enough, the same consolidation does not occur, and the low-activity mode remains comparatively stable. Thus, nonlinear transition structure and trust-gated preference plasticity jointly shape the Risk term so that the group can dwell near quasi-stable regimes for extended periods and then transition to other regimes as conditions shift, consistent with the defining “residence-and-switching” pattern of CI.

## 5. Numerical Results

This section presents the primary numerical evidence for our model. We prioritise the chaotic itinerancy (CI) test as the central dynamical signature: trajectories that dwell near multiple quasi-stable “attractor ruins” and switch irregularly among them. CI provides the most complete characterisation of the model’s complex dynamics, capturing the structured variability observed in real workshop settings.

As supplementary verification, we also report:

- Multiple-equilibria analysis: Demonstrating that multiple metastable regimes coexist, though not necessarily as strongly separated bistable attractors.
- Intervention response check: Confirming that perturbations produce distinct trajectories, a natural consequence of itinerant dynamics among multiple regimes.

The sensitivity analysis reveals the following key findings:

- Hill coefficient *n*: Bistability requires n≥4, and CI is robust across all bistable values (70–85%). The default n=4 achieves 85% CI pass rate while remaining within the biologically plausible range for cooperativity indices.
- Half-saturation constant *K*: Both bistability and CI depend sensitively on *K*. The bistable window spans K∈[0.27,0.70], with CI pass rates of 70–90% in the range K∈[0.20,0.60]. The default K=0.40 achieves robust CI (80%) at the center of the bistable region.
- Precision modulation λ: CI is remarkably robust across the entire tested range (λ∈[2,16]), with pass rates of 80–90%. This indicates that the precision-gated learning mechanism is not sensitive to the specific sharpness of the trust threshold.
- Coupling strength $\kappa_{S\to u}$: This parameter is the primary driver of both bistability and CI. CI occurrence increases monotonically with coupling strength, from 70% at κ=0.2 to 100% at κ=0.8. The default κ=0.6 balances robust CI with moderate multistability.

In summary, the model’s chaotic itinerancy is robust across a wide range of parameter values, with the trust–empowerment coupling strength acting as the primary control parameter.

### 5.1. Primary Test: Chaotic Itinerancy—Metastable Switching Among Attractor Ruins

The primary dynamical signature we target is *chaotic itinerancy* (CI): trajectories that dwell near multiple quasi-stable “attractor ruins” and switch irregularly among them [11,12,13,43]. Unlike simple bistability, CI emphasizes *structured variability*: the system neither converges to a single fixed point nor oscillates periodically. Instead, trajectories exhibit irregular residence times near multiple quasi-stable regimes, with transitions sensitive to both noise and intervention. This framework provides an interpretable vocabulary for small-group processes where collective modes (e.g., inhibited, playful, exploratory, confrontational) transiently stabilise and then reorganize.

Default parameter verification.

At the default parameters (N=6, n=4, $\sigma_{S}=0.03$, seed=1, T=400), the model passes all three primary CI indicators grounded in the theoretical literature:

1. Multiple attractor ruins [44]: 8 distinct metastable clusters detected via HDBSCAN.
2. Heavy-tailed residence times [45]: Skewness = 14.04 >1.5, indicating structured dwelling near quasi-stable regimes.
3. Local splitting exponent signature [46,47,48]: Mean ≈0 (0.025), substantial variance (σ=0.128>0.05), and frequent sign changes (27.3% >20%), consistent with alternating local stability and instability.

These three indicators directly operationalise the defining characteristics of chaotic itinerancy established in the theoretical physics literature.

To interpret the FTLE panel used in our diagnostic plots, note that a finite-time Lyapunov exponent (FTLE) is a local measure of how quickly nearby trajectories separate over a finite time window. In practice we estimate an FTLE proxy from the simulated trajectory embedded in a low-dimensional feature space (the same representation used for clustering into attractor ruins), and we compute a finite-window local divergence rate. Positive values indicate local expansion (instability; sensitivity to perturbations), whereas negative values indicate local contraction (stability; dwelling near an attractor ruin). Chaotic itinerancy is characterized by near-marginal stability: the FTLE fluctuates around zero with substantial variance and frequent sign changes, reflecting alternation between quasi-stable residence and transiently unstable transition epochs.

**Figure 2:**
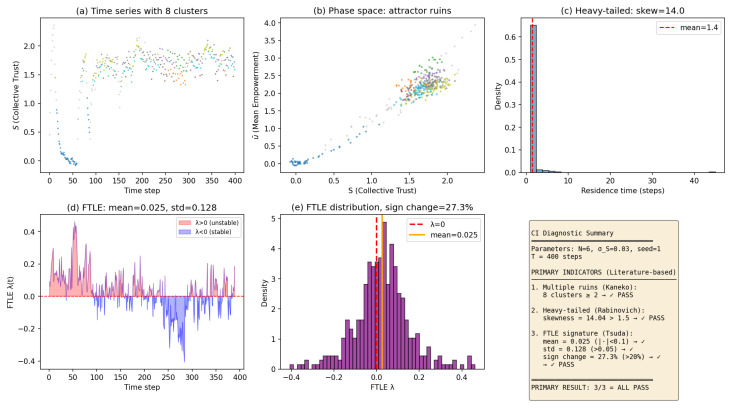
Chaotic itinerancy diagnostic panel under default parameters (N=6, n=4, $\sigma_{S}=0.03$, seed = 1). The six-panel display shows: (**a**) time series of collective trust *S* and expression rate $\bar{e}$; (**b**) phase space trajectory with color indicating time progression; (**c**) residence time distribution showing heavy-tailed structure; (**d**) FTLE time series with sign changes indicating alternating stability; (**e**) cluster membership over time; (bottom-right) action distribution across agents.

Figure 2 presents the comprehensive CI diagnostic panel for the default parameter configuration.

**Figure 3:**
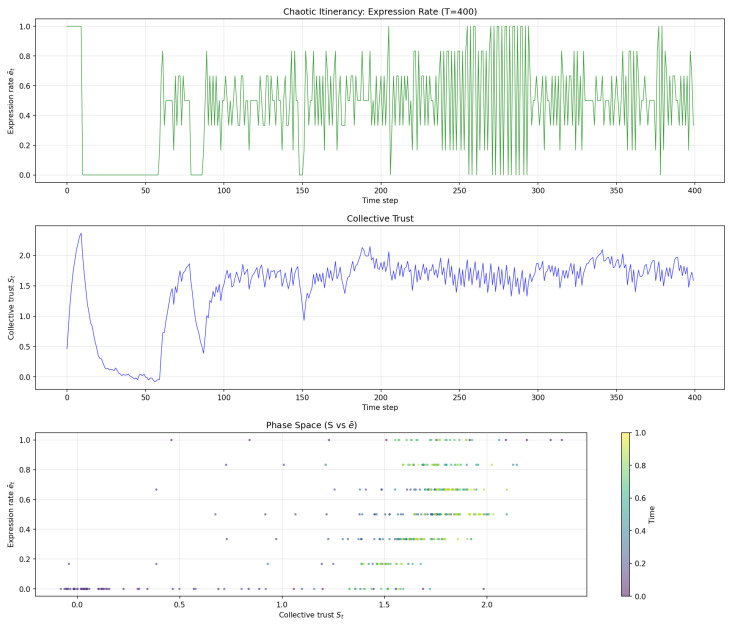
Chaotic itinerancy dynamics over 400 time steps. (**Top**) Expression rate ${\bar{e}}_{t}$ exhibiting irregular oscillations between high and low states. (**Middle**) Collective trust $S_{t}$ showing correlated fluctuations. (**Bottom**) Phase space plot (*S* vs $\bar{e}$) with color indicating time, revealing the trajectory’s visits to multiple quasi-stable regions.

Figure 3 shows the long-term chaotic itinerancy dynamics, demonstrating the irregular switching between high and low expression states.

Parameter scan (overview).

To assess robustness across parameter space, we conducted a parameter scan varying three control parameters. We ran T=400 time steps with N=20 agents and performed three trials per configuration. The scanned grid comprised $\beta_{\mathrm{action}}\in \left\{1,2,3,5,8\right\}$, n∈{4,6,8,10}, and trust-state noise $\sigma_{S}\in \left\{0.02,0.05,0.08,0.12\right\}$ (80 configurations total).

Each trial was evaluated using five indicators: the three primary CI indicators above, plus two supplementary operational indicators (transition-state fraction and action variability) that help identify promising parameter regions. The full indicator definitions and thresholds are reported in Supplementary Material (Section S-IV).

Results.

Across the 80 scanned configurations, 63 (78.8%) were classified as CI regions. Marginally, CI was most prevalent at intermediate trust noise ($\sigma_{S}=0.05$: 95% CI; other values: 70–80%), and increased with higher action precision ($\beta_{\mathrm{action}}=8$: 87.5% CI). Across Hill exponents, CI fractions were high for n=4 and n=10 (both 85%), and lower at n=6 (70%), suggesting that the degree of nonlinearity shapes whether trajectories become trapped or continue to switch among metastable regimes.

Table 6 reports the marginal CI fractions and mean indicator scores, averaged over the other scan dimensions.

These findings provide an additional robustness check beyond two-attractor multistability. They suggest that, for a broad parameter set, the same generative mechanisms that yield multistability and learning-induced hysteresis can also generate structured metastable switching compatible with CI-style dynamics, rather than requiring fine-tuned deterministic chaos.

**Figure 4:**
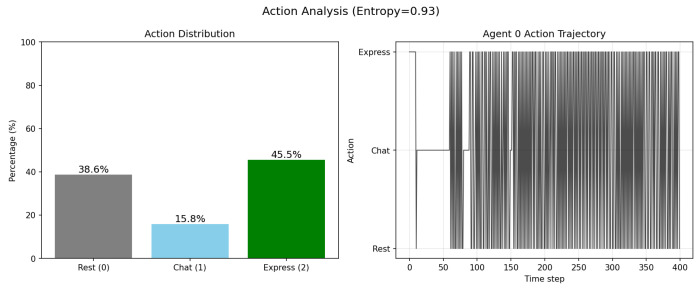
Action distribution across agents during CI dynamics. The distribution shows the relative frequency of Rest (0), Chat and Exercise (1), and Express (2) actions, revealing the collective behavioural patterns that emerge from individual Active Inference processes.

Figure 4 shows the distribution of actions across the agent population, illustrating the heterogeneity in behavioural choices.

### 5.2. Supplementary Checks: Multiple Equilibria, Intervention Response, and Phase Transition

Two supplementary checks and a phase-transition analysis further characterise the model’s dynamical landscape; full results, tables, and figures are provided in Supplementary Material (Section S-V).

Multiple-equilibria analysis.

Basin-of-attraction mapping across 16 initial conditions on a $\left(S_{0},u_{0}\right)$ grid reveals two well-separated attractors: a high-expression regime (${\bar{e}}^{*}\approx 0.51$, 75% of initial conditions) and a low-expression regime (${\bar{e}}^{*}\approx 0.00$), with separation degree $\Delta_{\mathrm{sep}}=1.65$. This confirms that the model supports multiple coexisting metastable regimes—the “attractor ruins” among which CI trajectories wander.

- Intervention response.

Pulse interventions at t=50 (high: S→0.9 vs. low: S→0.1) from identical initial conditions produce distinguishable trajectory ensembles ($|\Delta {\bar{e}}_{\mathrm{avg}}|=0.10$, 5-seed average), demonstrating that facilitator interventions can redirect collective dynamics. The mechanism is learning-induced: interventions leave lasting traces in agents’ preference parameters ($\mu_{U}$) via precision-gated updates, rather than merely shifting state trajectories.

Phase transition characteristics.

Varying the half-saturation constant *K* reveals a bistable window K∈[0.27,0.70] flanked by monostable regimes (high-activity for K<0.27; low-activity for K>0.70), with maximum attractor separation at K=0.557.

### 5.3. Chat and Exercise to Express Induction

A key design feature of the present model is that Chat and Exercise provides information gain about interpersonal trust (${\mathrm{IG}}_{W}>0$) while Express does not, creating an incentive for agents to first engage in curiosity-driven exploration before transitioning to coordination-driven participation. Empirical analysis of action transitions confirms this prediction: Chat and Exercise → Express transitions occur (164 instances across 10 seeds), while Express → Chat and Exercise transitions do *not* occur (0 instances), revealing a unidirectional induction pattern. Furthermore, periods of high Chat and Exercise activity are followed by modest increases in collective expression rate ($\mathrm{Corr}({\bar{c}}_{t},\Delta {\bar{e}}_{t+1})=+0.027$). To further validate this causal relationship, we applied Transfer Entropy analysis [49], which quantifies directed information flow between time series. The results show TE(ChatandEx.→Express)=0.111 bits versus TE(Express→ChatandEx.)=0.043 bits (ratio =2.57, both p<0.001), confirming significantly stronger information flow from Chat and Exercise to Express than vice versa. This supports the interpretation that Chat and Exercise serves as a “gateway” action enabling subsequent Express behaviour, consistent with theatre workshop dynamics where participants first gauge group receptivity through low-stakes conversation and exercises before committing to expressive improvisation. Full details of the transition and Transfer Entropy analyses are provided in Supplementary Material (Section S-XI). A sensitivity analysis of the Express information gain assumption (${\mathrm{IG}}_{W}\left(\mathrm{Express}\right)=0$) is provided in Supplementary Material (Section S-X).

### 5.4. Comparison with Simplified Model

To demonstrate the necessity of the key mechanisms in our model, we conducted a systematic chaotic itinerancy (CI) analysis comparing three model conditions:

1. Full Model (Nonlinear): Default parameters with Hill function active ($c_{S,\mathrm{hill}}=0.5$, $K_{\mathrm{hill}}=0.4$).
2. Linear Model: Hill function coefficient set to zero ($c_{S,\mathrm{hill}}=0$), removing the nonlinear expression–trust coupling.
3. No Coupling: Half-saturation constant set to zero (K=0), which saturates the Hill function ($H_{n}\approx 1$ for all $\bar{e}>0$). This removes the cooperative threshold: any nonzero expression triggers the full trust amplification, eliminating the expression-rate-dependent nonlinearity that generates distinct collective regimes.

Table 7 summarizes the CI detection results across 10 independent simulation runs with N=6 agents over T=400 time steps:

The results clearly demonstrate:

1. Nonlinear Hill function enhances CI: The full model achieves 80% CI pass rate compared to only 20% for the linear model—a fourfold improvement. The Hill function’s sigmoidal response creates the threshold-crossing dynamics essential for attractor ruin formation.
2. Cooperative threshold is essential: The no-coupling condition (K=0) shows 0% CI pass rate with only 1/3 primary indicators consistently detected. When the Hill function is saturated (removing the critical-mass threshold), the trust amplification becomes expression-rate-independent, destroying the bistable attractor landscape and collapsing the system to simple fixed-point dynamics.
3. Cluster structure differs qualitatively: At seed = 1, the full model detected 10 attractor ruins with 23% noise ratio (healthy transition states), while the linear model detected only two clusters with 56% noise ratio (excessive transitions), and the no-coupling condition showed two clusters with only 4% noise ratio (near-fixed-point behaviour).

These findings establish that the nonlinear Hill function coupling is not merely a parameter choice but a necessary structural feature for generating the rich attractor landscape that supports chaotic itinerancy. The simplified linear model cannot produce the itinerant dynamics that characterise theatre workshop processes.

The linear model ($c_{S,\mathrm{hill}}=0$) effectively reduces the trust dynamics to a first-order autoregressive process with additive social input—structurally analogous to mean-field Ising models with Glauber dynamics, where action probabilities depend linearly on the social field [50]. The dramatic reduction in CI pass rate from 80% to 20% under linearisation quantifies the cost of removing the affordance structure that distinguishes our approach from classical social interaction models.

### 5.5. Parameter Sensitivity Analysis

To assess the robustness of our results to specific parameter choices, we conducted systematic sensitivity analyses for four key parameters: the Hill coefficient *n*, the half-saturation constant *K*, the precision modulation sharpness λ, and the trust–empowerment coupling strength $\kappa_{S\to u}$. Each parameter was tested for both bistability (multiple equilibria) and chaotic itinerancy (CI) across multiple seeds. Table 8 summarizes the main findings; full details are provided in Supplementary Material (Section S-VI).

Key findings: (i) *Hill coefficient n*: Bistability requires n≥4; CI robust at 70–85% for bistable values (default *n* = 4: 85%). (ii) *Half-saturation K*: Bistable window K∈[0.27,0.70]; CI 70–90% in [0.20,0.60] (default 0.40: 80%). (iii) *Precision λ*: CI robust (80–90%) across [2,16]. (iv) *Coupling $\kappa_{S\to u}$*: Primary driver; CI increases monotonically 70% → 100% (default 0.6: 80%).

In summary, the model’s chaotic itinerancy is robust across a wide range of parameter values, with the trust–empowerment coupling strength acting as the primary control parameter. The Hill coefficient exhibits a critical threshold effect, while the precision modulation mechanism shows remarkable insensitivity to specific parameter choices.

## 6. Discussion

The numerical results establish chaotic itinerancy as the primary dynamical signature of our model: trajectories dwell near multiple quasi-stable collective modes and switch irregularly among them. This section discusses the theoretical implications of this finding, its relation to existing frameworks, and practical consequences for workshop facilitation.

### 6.1. On Social Priors and the Minimality of Preference Specification

Several Active Inference models of social behaviour introduce explicitly social or normative structure through prior preferences over outcomes. For example, Constant et al. formalise deontic value/cues, whereby observations can endow policies with normative salience [5], and related work on cooperative communication posits an adaptive prior belief of mental-state alignment [6]. More generally, Active Inference often represents goals/values as prior distributions over preferred observations [41,51].

While these approaches provide an alternative to utility functions, they raise a concern about how much of the social phenomenon is effectively specified in outcome-level preferences. When conformity or coordination is encoded directly in preference distributions, explanations risk circularity: the target pattern is assumed at the level of priors.

The present multi-agent EFE model adopts a different strategy. We do not attempt to remove priors from Active Inference, but we restrict *where* social structure is introduced: collective effects enter via state-transition structure and learning conditions, while preferences are restricted to broadly non-normative state variables (trust, empowerment, stamina) that shape feasibility and learning rather than directly prescribing a collective outcome.

Structural constraints vs. outcome-level priors: One might object that embedding Hill-type nonlinearity in state transitions constitutes a form of “structural prior knowledge” about social dynamics. We acknowledge this, but emphasise a crucial distinction. The Hill function specifies *affordances*—environmental conditions that make certain actions more or less feasible—rather than *preferences* over collective outcomes. Concretely, the Hill term encodes that (i) sustained expression is physiologically costly without group support, and (ii) trust builds more readily when collective participation exceeds a threshold. These are constraints on *what is possible* given the physical and psychosocial environment, analogous to how gravity constrains movement without prescribing where one should go. In contrast, outcome-level social priors would directly encode “high collective expression is preferred” or “conformity is good” in the preference distribution. Our model contains no such specification: agents prefer states of high trust, empowerment, and stamina for individual reasons, and collective patterns emerge from the interplay of these preferences with the affordance structure.

Moreover, empowerment preference updating is conditional on inferred trust. This yields *conditional preference plasticity* (a second-order dependence of learning on context) that can regulate exploration and learning without encoding a specific social goal at the outcome level. In a limited sense, the model involves a weak structural form of “social prior” insofar as preferences are defined over socially relevant latent variables; this differs from outcome-level priors that prescribe collective behaviour (e.g., conformity) as a preferred observation.

Accordingly, the collective phenomena in the simulations arise from individual inference, resource constraints, and coupling through shared latent variables and trust-gated learning, rather than from direct outcome-level social preference specifications.

Within the Active Inference literature, there is growing interest in belief sharing and multi-agent dynamics. Work that is closely related to the current treatment includes the explicit modelling of belief sharing in epistemic communities [52,53]. A simpler approach considers collective behaviour under mere Active Inference: i.e., ignoring the epistemic aspects of dyadic and group interactions to simulate swarming or collective behaviour [54]. There is also a body of work on federated inference and the emergence of communication as a free energy minimising process [53]. Finally, there is recent work looking at the relationship between generative models entertained by the members of a group and the implicit generative model underlying the behaviour of the group per se [55]. Our focus is on the collective dynamics and in particular the itinerancy and metastability that characterises extended group behaviours. These are nicely characterised by the insights afforded by the theatre workshop model.

### 6.2. Emergent Chaotic Itinerancy Without Social Priors

Our simulations exhibit chaotic itinerancy under EFE minimisation without adding explicit social conformity utilities. Positive feedback loops are encoded in the *generative model* (state-transition dynamics) rather than as direct outcome-level preferences.

This result resonates with an early demonstration in the FEP literature: Friston et al. showed that an active-inference agent endowed with strong priors for Lorenz-attractor dynamics can generate chaotic trajectories through prediction-error suppression alone [14]. Our model extends this principle to a multi-agent social setting, where collective chaotic itinerancy emerges from individual EFE minimisation without requiring explicit chaos-generating mechanisms in the policy.

Crucially, chaotic itinerancy differs from simple bistability. Bistable systems exhibit two well-separated attractors with deterministic convergence to one or the other. In contrast, CI involves *quasi-stable* regimes (“attractor ruins”) that trajectories visit transiently before transitioning to other regimes. This structured variability—neither convergence to a single point nor random noise—captures the exploratory nature of real theatre workshop dynamics.

The three mechanisms play distinct roles:

1. Cooperative cost reduction creates immediate incentives to express when others do.
2. Trust–empowerment coupling amplifies empowerment gains in trusting environments.
3. Comfort zone expansion generates hysteresis by making preference changes irreversible.

### 6.3. Mechanisms of Collective Dynamics

The rich dynamical behaviors observed in the model—specifically chaotic itinerancy and the Chat and Exercise to Express induction—emerge from the interplay of three core mechanisms.

1. Trust–Empowerment Coupling (Global Amplification).

The coupling term $\kappa_{S\to u}$ creates a positive feedback loop between collective trust *S* and individual empowerment *u*. In high-trust regimes, the actions of others are perceived as more empowering, which in turn encourages further expression. This mechanism stabilizes the high-activity “attractor ruin,” allowing the group to sustain periods of intense collaboration. Conversely, in low-trust states, this amplification is absent, trapping the group in quiescence until a fluctuation or intervention occurs.

2. Precision-Gated Preference Learning (Adaptive Hysteresis).

Instead of a fixed “comfort zone,” agents dynamically update their preference parameters ($\mu_{U}$) based on experience. Crucially, this learning is gated by trust: only when *S* is high do agents treat their empowerment gains as reliable signals for updating their self-model (increasing $\mu_{U}$). This creates a “ratchet effect” where successful collective episodes leave a lasting trace in the agents’ internal priors, preventing a simple return to the initial state and generating the path dependence observed in the intervention tests.

This mechanism aligns with psychological research on self-efficacy [35], which emphasizes that success experiences are most impactful in supportive environments. From a practical standpoint, this suggests that interventions should prioritise building trust before encouraging action. A temporary “push” toward high activity is ineffective unless the environment supports the internalisation of positive experiences.

3. Network-Mediated Induction (Local-to-Global Propagation).

A key innovation in the present model is the role of the social network and interpersonal trust $W_{ij}$. The “Chat and Exercise” action serves as a low-risk mechanism to build $W_{ij}$ without requiring immediate global exposure. As local dyads build trust, the effective cost of “Express” decreases (via the synchrony term), eventually triggering a local cluster of expression. This local activity then feeds into the global trust *S*, potentially igniting a system-wide phase transition. This two-stage process—Chat and Exercise building local $W_{ij}$, leading to Express building global *S*—explains the unidirectional “Chat and Exercise → Express” induction observed in the results.

### 6.4. Functional Role of Model Mismatch

The agents’ internal model intentionally omits the complex trust–empowerment coupling term. This “bounded rationality” design forces agents to treat the amplified empowerment from social interactions as positive *surprise* (prediction error) rather than a predicted outcome. This continuous stream of surprise drives exploration and prevents the system from settling into a static equilibrium, thereby sustaining the itinerant dynamics essential for creative collaboration. This mechanism complements the chaos-control perspective of [14]: whereas strong, accurate priors can *stabilize* chaotic trajectories via error suppression, our incomplete-model agents experience persistent prediction errors that *sustain* exploratory dynamics.

We investigated this question by comparing simulations under two conditions:

1. Baseline (Incomplete Model): Agents predict empowerment without the trust coupling term (current implementation);
2. Complete Model: Agents include the full trust–empowerment coupling $\kappa_{S\to u}$ in their predictions.

Behavioural results.

Surprisingly, the complete-model agents exhibit zero collective expression, collapsing entirely to the quiescent low-activity state (expression rate $\bar{e}=0.00$, zero state transitions). Rather than exploiting their knowledge of the coupling to reach and maintain the high-activity attractor, omniscient agents become perfectly conservative. In contrast, the baseline (incomplete model) agents show robust chaotic itinerancy with an average of 55.2±27.9 state transitions and expression rate $\bar{e}\approx 0.36$ per 400-step trajectory (Table 9).

CI diagnosis reveals spurious clustering.

Applying the three primary CI indicators (Section 5.1) to the complete-model trajectories yields an instructive pattern: indicators 1 (attractor ruins) and 2 (heavy-tailed residence times) pass, while indicator 3 (FTLE signature) fails due to insufficient variance (σ=0.027<0.05). However, these “passing” indicators are spurious: they detect micro-fluctuations in the agents’ *belief space* (the internal variables $\mu_{S}^{i}$, $\mu_{U}^{i}$ that continue to update even without behavioural expression) rather than meaningful behavioural dynamics. The complete-model agents remain behaviourally frozen at zero expression throughout the entire simulation; the clustering algorithm merely partitions trivial belief-space wandering that has no behavioural consequence. This underscores the importance of interpreting CI indicators in conjunction with behavioural metrics: a trajectory with zero expression and zero state transitions cannot exhibit meaningful chaotic itinerancy regardless of belief-space clustering patterns.

Interpretation.

This finding suggests that model mismatch is not merely a simplifying assumption but a functional design feature that promotes healthy collective dynamics:

- Surprise as exploration driver: Prediction errors generate surprise signals that prevent premature convergence;
- Avoiding coordination failure: Complete knowledge of interdependencies can lead to “free-riding” expectations that precipitate collective inaction;
- Bounded rationality benefits: Consistent with game-theoretic results showing that bounded rationality can improve collective outcomes [56].

This result aligns with ecological rationality perspectives arguing that cognitively simple heuristics can outperform optimal strategies in complex environments [57]. The agents’ “ignorance” of the trust coupling generates the variability and exploration necessary for discovering and transitioning between collective modes—the very signature of chaotic itinerancy.

### 6.5. The Empowerment Expansion as Self-Recognition: Removal of Habitual Defenses

The latent empowerment expansion variable $z_{t}^{i}$ (Equation (31)) deserves deeper theoretical interpretation. Rather than functioning as a mere computational switch, $z_{t}^{i}$ represents the agent’s inference about whether it has crossed a threshold—a form of *self-recognition* regarding one’s own transformative state. In the framework of the Free Energy Principle, this can be understood as higher-order model selection: the agent infers “I am now in a growth mode” versus “this experience was circumstantial.”

Connection to Theatre Studies: Breaking Habitual Defenses

This computational mechanism formalizes insights from theatre theory. Grotowski’s concept of the “via negativa”—the removal of habitual psychological and physical defenses—describes precisely the kind of threshold-crossing that $z_{t}^{i}$ captures [7]. The agent does not add new techniques but rather *removes* the protective shell that prevents authentic expression. When $u_{t}^{i}$ exceeds $\mu_{U}^{i}+\theta_{\mathrm{gap}}$, the agent recognizes that it has acted beyond its prior defensive boundary.

Accepting a New Self

The unidirectional nature of preference updates (the “ratchet effect”) reflects a deeper psychological reality: once one has genuinely experienced expanded capability and recognized it as authentic ($z_{t}^{i}=1$), the prior, more limited self-model becomes difficult to maintain. This is not mere behavioural change but a shift in *what the agent believes it can be*—an update to its generative model of self.

From the Active Inference perspective, $z_{t}^{i}$ can be interpreted as evidence for a higher-level hypothesis: “I am someone who can act and express in this way.” The precision-gated learning mechanism ensures that this evidence is only accepted when contextual conditions (trust) support reliable inference. In this sense, the model captures the interplay between environmental safety, threshold-crossing action, and identity transformation that practitioners of theatre have long recognized.

### 6.6. Quantitative Assessment of the Dual-Gate Mechanism

A natural question is whether the empowerment expansion gate $z_{t}^{i}$ is *essential* for the ratchet effect, or whether trust-weighted precision alone suffices. We conducted a systematic comparison of three conditions (see Supplementary Material (Section S-VII) for full details):

1. Double-Gate: The current implementation, where preference updates require both high precision (trust-dependent) and mastery detection ($z_{t}^{i}=1$);
2. Single-Gate: Precision weighting only, with mastery gate bypassed (always $E[z_{t}^{i}]=1$);
3. No-Gate: Both gates bypassed (always high precision, always updating).

The quantitative results (Table 10) reveal that the mastery gate provides incremental improvement rather than being strictly necessary for irreversibility. The ratchet ratio (upward/downward update events) was 4.5 for Double-Gate, 3.8 for Single-Gate, and 3.3 for No-Gate. All conditions exhibited net positive preference expansion (approximately 0.7 units), with similar maximum retracements (0.14–0.15 units).

These results support the interpretation that the primary source of irreversibility is the unidirectional constraint (upward_only=True), which prevents downward preference updates regardless of gate configuration. The mastery gate $z_{t}^{i}$ provides an additional filtering mechanism that:

- Increases the ratchet ratio by approximately 17% (from 3.8 to 4.5) compared to precision-only updates;
- Filters learning signals to internalise only genuine threshold-crossing experiences;
- Provides biological and psychological plausibility, as discussed in Section 6.5.

The dual-gate design is therefore best understood as a *quality-enhancing* mechanism rather than an absolute requirement for irreversibility. It ensures that the learning signal is concentrated on pedagogically meaningful events—moments of genuine breakthrough—rather than being diluted by routine fluctuations. This is consistent with educational research emphasizing that not all successes are equally formative; breakthrough moments that exceed prior self-expectations carry special developmental significance.

Beyond preference learning dynamics, the gating mechanism operates within a dynamically rich substrate. Our chaotic itinerancy analysis (Section 5.4) demonstrates that the nonlinear Hill function coupling is essential for generating CI: the full model achieves 80% CI pass rate compared to only 20% for a linearized version and 0% when expression–trust coupling is disabled entirely. This establishes that the dual-gate mechanism filters transitions within a complex attractor landscape that itself depends critically on the nonlinear feedback structure.

### 6.7. EFE Component Analysis During Transitions

A detailed analysis of EFE component contributions during transitions is provided in Supplementary Material (Section S-XV). The key finding is that trust-related risk (${\mathrm{Risk}}_{S}$) dominates at 62% of total Risk, while interpersonal trust information gain (${\mathrm{IG}}_{W}$)—obtained exclusively through Chat and Exercise actions—accounts for 44% of total information gain, validating the epistemic role of Chat and Exercise in the model design.

### 6.8. Relationship Between Chaotic Itinerancy and Mutual Entrainment

We also analyzed the relationship between chaotic itinerancy and mutual entrainment using a Kuramoto Order Parameter adapted for discrete action spaces; see Supplementary Material (Section S-XVI) for details. The analysis reveals irregular switching between synchronized and desynchronized collective states—8 transitions over 400 time steps—consistent with CI as a mechanism for synchronisation–desynchronisation alternation [58,59]. Notably, action-level variability coexists with highly convergent internal states (empowerment synchrony R≈0.99, belief synchrony R≈0.998), suggesting that agents develop shared beliefs while maintaining behavioural diversity.

### 6.9. Implications for Workshop Management

**Priority of trust building** Rather than immediately encouraging expression, it is important to first raise field trust. Comfort zone expansion does not occur unless trust exceeds the threshold.
**Timing of intervention** When trust is near the threshold, small interventions have large effects. Facilitators need to discern the “critical point.”
**Conditions for sustained change** For “growth” accompanied by preference change rather than temporary excitement, successful experiences under sufficient trust are necessary.

### 6.10. Novelty Beyond Ising-Type Models

Ising-type social models typically specify an energy function (Hamiltonian) *a priori*; multistability (including bistability) and hysteresis follow as relaxation toward energy minima. The kinetic Ising model and social extensions [50,60] exemplify this setup, with Glauber dynamics yielding logit-type transition rates from the Hamiltonian.

Our model differs in five ways, reflecting a richer structural complexity:

(i) Action selection emerges from inference, not from an imposed energy function.

Although the update resembles a Glauber-type logit form, we do *not* posit a “social Hamiltonian” or “conformity utility.” Action probabilities arise from Expected Free Energy (EFE) minimisation, combining risk (KL divergence from preferred outcomes) and epistemic value. The decomposition −logP(e∣h)=Risk+Ambiguity [4] provides a generative derivation rather than an ad hoc energy specification.

(ii) The latent field *S* is inferred, not externally imposed, and observation precision depends on action.

In standard Ising models, the external field is exogenous; here, collective trust *S* is a shared latent variable inferred from noisy observations via an Extended Kalman Filter. Crucially, observation noise R(e) is action dependent: expressing (e=1) yields more precise observations than remaining silent (e=0). This “active observation” (action-modulated epistemic access) is central to POMDP/Active Inference architectures and not part of equilibrium Ising formulations.

(iii) Stamina *H* endogenizes effective temperature and action costs, with collective-state-dependent modulation.

The fatigue/resource constraint produces a time-varying effective inverse temperature $\beta_{\mathrm{eff}}$, yielding intrinsic non-equilibrium dynamics. In addition, the Hill term $H_{n}\left(\bar{e};K\right)$ reduces expression costs when collective activity is high, i.e., the group state modulates individual action costs. This feedback goes beyond the additive pairwise interactions typical of Ising extensions.

(iv) Comfort zone expansion generates learning-based path dependence distinct from equilibrium hysteresis.

Ising hysteresis under field sweeps is state-level and reversible in principle; here, path dependence is introduced by an irreversible preference-jump mechanism:

(58) $$S>\theta_{S}\;\wedge \;\mathrm{experience}>\mu_{U}\;⟹\;\mu_{U}\leftarrow \mu_{U}+\Delta \mu$$

Even if the system returns to the same *state* $\left(S,\bar{e}\right)$, the agent’s internal preferences remain changed. Thus, history is encoded in parameter updates rather than in state trajectories, yielding a non-Markovian effect.

(v) Network topology and dyadic trust ($W_{ij}$) introduce local–global interplay.

Unlike mean-field Ising models where every agent interacts with the global average, our model incorporates a sparse social network where agents infer local interpersonal trust $W_{ij}$. These dyadic weights evolve based on interaction synchrony and are learned via Variational Message Passing, allowing agents to form distinct local relationships. This structure enables complex spatiotemporal patterns where local pockets of trust can emerge and propagate, creating a richer dynamical landscape than the uniform phase transitions of fully connected mean-field models. Furthermore, the specific epistemic role of Chat and Exercise in reducing uncertainty about $W_{ij}$ adds a strategic dimension to local relationship building that is absent in standard spin systems.

In summary, the model can reproduce multistable collective dynamics but differs in generative structure: probabilistic inference under partial observability with action-dependent precision, endogenous non-equilibrium effects from resource constraints, learning-based path dependence via irreversible preference updates, and rich local–global network interactions.

Connection to Statistical Physics.

Despite these structural differences, the model exhibits characteristics familiar from statistical mechanics:

- Phase transitions: Sharp changes in collective behaviour at critical parameter values.
- Hysteresis: The system’s state depends on its history.
- Critical slowing down: Near the transition point, fluctuations are amplified.

The Hill function plays a role analogous to interaction energy in spin systems, with the Hill coefficient *n* controlling the sharpness of the transition, analogous to inverse temperature. In brief, our approach places the explanatory burden on inference under partial observability and learning rules in the generative model (including action-dependent observation precision and trust-gated preference plasticity), rather than on an externally specified social Hamiltonian.

Table 11 summarizes the structural differences between the present model and representative approaches from sociophysics, small-group dynamics, and Active Inference social modelling.

### 6.11. Robustness of Chaotic Itinerancy Across Network Topologies

To assess whether chaotic itinerancy depends on the specific choice of Erdős–Rényi (ER) topology used in the main simulations, we repeated the baseline experiment (N=6, T=400, average degree $k_{\mathrm{avg}}=4$, 10 seeds per topology) on Watts–Strogatz (WS) small-world and Barabási–Albert (BA) scale-free networks. Table 12 summarises the results. CI emerges across all three topologies, confirming that Hill-function nonlinearity—not network structure—is the essential mechanism for metastable switching. ER networks show the highest CI pass rate (70%), consistent with their relatively homogeneous degree distribution. WS networks produce more frequent state transitions (145.1 vs. 90.4), likely because their high clustering coefficient creates local feedback loops that destabilise dwell states. BA networks exhibit the lowest CI rate (40%), suggesting that hub structure concentrates social influence and stabilises the collective dynamics, making transitions less frequent.

### 6.12. Effect of Agent Heterogeneity on Chaotic Itinerancy

The baseline model assumes homogeneous agents. To probe robustness to inter-individual variation, we introduced heterogeneity by drawing each agent’s parameter from a Gaussian distribution centred on the default value with coefficient of variation (CV) chosen at the largest value that still maintains CI at baseline levels. Table 13 reports results at these CI-maintenance thresholds. Moderate heterogeneity in initial preference ($\mu_{U,\mathrm{init}}$, CV = 0.08) or empowerment gain ($\eta_{\mathrm{self}}$, CV = 0.02) slightly *increases* the CI pass rate to 80%, likely because inter-agent variability seeds symmetry-breaking fluctuations that facilitate transitions. Decision-style heterogeneity ($\beta_{\mathrm{action}}$, CV = 0.20) leaves CI unchanged at 70%.

The sensitivity of CI to heterogeneity differs markedly across parameters (Table 14). $\beta_{\mathrm{action}}$ is the most robust: CI persists up to CV ≈ 0.20. $\mu_{U,\mathrm{init}}$ shows moderate sensitivity (threshold CV ≈ 0.08), while $\eta_{\mathrm{self}}$ is highly sensitive (threshold CV ≈ 0.02). The sensitivity of $\eta_{\mathrm{self}}$ is interpretable: this parameter governs how much psychological fulfillment an agent derives from self-expression, and when individuals differ substantially in this gain, the bistable attractor structure collapses because agents no longer cross the expression threshold in a coordinated manner. For workshop practice, this suggests that forming subgroups with similar psychological readiness for expression—rather than mixing widely different experience levels—may preserve the creative itinerant dynamics essential for workshop effectiveness.

### 6.13. Quantitative Comparison with Mean-Field Social Interaction Model

To sharpen the contrast with Ising-type models discussed qualitatively in Section 6.10, we implemented the Brock–Durlauf discrete-choice model with social interactions [50] as a quantitative baseline. We calibrated the Ising baseline to produce a similar mean expression rate, then compared CI diagnostics (Table 15). The Ising baseline achieves 0% CI pass rate, near-zero variance in $\bar{e}$ (no bistability), and high action entropy (near-random switching). This confirms that EFE minimisation with structured generative models—Hill-function nonlinearity, trust-gated learning, and action-dependent observation precision—is necessary for generating chaotic itinerancy; simple social conformity pressure, even when calibrated to match aggregate statistics, is insufficient.

### 6.14. Limitations and Future Directions

Several limitations of the present study also motivate concrete directions for future work.

#### 6.14.1. Network Structure and Remaining Extensions

Our model extends beyond the mean-field approximation by embedding agents in an Erdős–Rényi network with dynamic interpersonal trust weights $W_{ij}$. Each agent observes a *local* expression rate ${\bar{e}}^{\left(i\right)}$ computed as a trust-weighted average over its neighborhood (Equation (10)), rather than the global expression rate ${\bar{e}}_{t}$. This network structure addresses several limitations of pure mean-field models:

Advantages of the Network Extension

- Heterogeneous influence: Agents experience differentiated social influence based on their network position and interpersonal trust levels.
- Spatial correlation: Clustering and subgroup formation can emerge naturally from network topology.
- Appropriate for small groups: Sparse networks with $k_{\mathrm{avg}}=4$ are more realistic for typical workshop sizes of 8–20 participants.
- Curiosity-driven exploration: The Chat and Exercise action provides information gain about interpersonal trust, implementing a distinct behavioural mode from expression-driven coordination.

Remaining Directions

While the network extension addresses the core limitation of mean-field coupling, several research directions remain:

1. Topology effects: How do small-world, scale-free, or clustered network structures affect chaotic itinerancy? The current Erdős–Rényi topology could be replaced with more structured alternatives [2].
2. Dynamic networks: The current model uses fixed network topology with dynamic edge weights. Extending to time-varying network structure (edge creation/deletion) would capture evolving interaction patterns.
3. Role differentiation: Workshop facilitators could be modelled as hub nodes with enhanced connectivity or modified action preferences.

Beyond the Hill-function nonlinearity employed here, alternative forms of nonlinear state transition dynamics could be explored. Recent advances in chaotic map design, including higher-dimensional logistic maps [61] and sine-based reconstruction approaches [62], offer additional perspectives on generating and controlling complex nonlinear dynamics that may inform future extensions of the state transition model.

#### 6.14.2. Agent Heterogeneity and Further Action Richness

Our model extends the action space from binary (rest/express) to ternary (rest/chat and exercise/express), enabling distinct behavioural modes for curiosity-driven social exploration (Chat and Exercise) versus expression-driven coordination (Express). However, agents remain homogeneous in their parameters and preferences. Future extensions could introduce:

- Heterogeneous agents: Variation in baseline stamina, trust sensitivity, or preference parameters across agents.
- Graded actions: Continuous-valued expression intensity rather than discrete actions. The current three-valued action space reflects a deliberate design choice grounded in the first author’s practitioner experience: in theatre workshops, the decision to commit to self-expression constitutes a qualitative threshold crossing rather than a continuous intensity variation. Preparatory activities involving variations in expressive intensity, technique, and time allocation correspond to the Chat and Exercise stage in the present model. Nevertheless, extending to continuous action spaces via policy-gradient-based Active Inference methods [63,64] would enable finer-grained modelling of expressive behaviour.
- Role-specific preferences: Facilitators with different preference structures or action costs.

#### 6.14.3. Empirical Grounding

A promising next step is to operationalise the model’s notion of “expression” and map it to observable features extracted from real-time workshop recordings. In practice, expressive acts can be represented by time-stamped behavioural markers derived from video and audio streams, such as speaking turns, gesture amplitude, body orientation toward the group, movement initiation, prosodic intensity, interpersonal distance changes, and dyadic synchrony measures. These features can be aggregated into a continuous-valued proxy for expression intensity, thereby replacing the binary action variable with a measured or inferred action process.

Given such observations, the model can be extended into a state-space estimation problem in which latent variables—most notably the field-level trust state $S_{t}$ and participant-level empowerment $u_{t}^{i}$ (and potentially fatigue-related states beyond the present stamina proxy $H_{t}^{i}$)—are inferred from multimodal data. This can be approached using standard Bayesian filtering and smoothing techniques (e.g., extended/unscented Kalman filtering, particle filtering, or variational message passing) applied to a suitably parameterized observation model that links latent states to behavioural features. In this empirical extension, workshop facilitation becomes a partially observed control problem: interventions can be treated as exogenous inputs that transiently perturb action tendencies or observation precision, allowing one to quantify how timing and intensity of facilitation shift the system across critical thresholds.

Beyond retrospective analysis, a longer-term goal is to enable real-time, model-based support for workshop facilitation. If latent trust and empowerment states can be tracked online, the model may provide principled indicators of proximity to transition points (critical slowing down, rising variance, or increasing sensitivity to perturbations), thereby informing when minimal interventions are most effective. This connects to a general property of free energy minimising systems: critical slowing down emerges naturally near phase transitions as the curvature of the free energy landscape flattens, increasing the system’s sensitivity to perturbations and the time required to return to equilibrium [14]. Such an approach would move toward an adaptive facilitation loop, in which the workshop is managed not by enforcing predetermined outcomes but by monitoring and supporting the conditions under which constructive collective transitions—such as sustained engagement and comfort-zone expansion—can emerge.

Finally, developing empirical protocols will require careful attention to ethics, privacy, and interpretability. Any real-time inference system must respect participants’ consent and data governance, and should be designed to augment, rather than replace, human facilitation. Nevertheless, the present model suggests a principled pathway from theory to practice: by linking expressive behaviour to observable features and treating workshop dynamics as a coupled inference-and-control process, Active Inference-based models may contribute to richer, evidence-informed workshop design and facilitation.

## 7. Conclusions

This study has developed a multi-agent model of collective dynamics in theatre workshops, grounded in Active Inference and Expected Free Energy minimisation. The central finding is that chaotic itinerancy—structured switching among multiple metastable collective modes—emerges naturally from EFE minimisation with trust-gated preference learning, without prescribing collective outcomes as preferred observations.

The main contributions are organized into two categories: primary theoretical contributions and the methodological elements that support them.

Primary Contributions

1. Emergent Chaotic Itinerancy Without Outcome-Level Social Priors: We demonstrated that chaotic itinerancy can emerge from EFE minimisation when collective effects are encoded in the *predictive model* (state transition dynamics) rather than in explicit social preference terms. Trajectories dwell near multiple quasi-stable “attractor ruins” and switch irregularly among them, producing structured variability rather than convergence to a single fixed point. This maintains the principled derivation of behaviour from probabilistic inference while avoiding the circularity of directly prescribing collective outcomes through priors. Crucially, this result successfully reproduces the dynamic process of theatre workshops—where groups alternate between periods of high energy and quiescence—providing a novel computational approach to theatre analysis that has not been seen in previous studies. Supplementary checks confirmed intervention responsiveness and the presence of multiple coexisting metastable regimes.
2. Precision-Gated Preference Learning: Rather than employing ad hoc threshold rules for preference changes, we derived a principled variational mechanism in which preference parameters are hierarchically embedded as latent hyperstates subject to precision-weighted Bayesian updating. Trust modulates learning precision, providing a principled foundation for “comfort zone expansion” that generalizes previous threshold-based formulations.
  In the Theatre workshop interpretation, this hierarchical mechanism captures a clinically salient “threshold-crossing” process. Under sufficient inferred trust, positive empowerment overshoots are internalized as durable preference shifts, yielding a qualitative change in an agent’s expressive agency. This provides a minimal computational account of the transition from inhibited participation to sustained risk-taking self-disclosure without prescribing the collective outcome at the level of social priors.

Methodological and Technical Contributions

To realise and validate the above contributions, we developed the following technical framework:

1. Interpersonal Trust Network with Local Averaging: We extended the mean-field collective dynamics to an explicit network model, where agents are embedded in a sparse Erdős–Rényi graph with dynamic interpersonal trust weights $W_{ij}$. Local expression rates are computed as trust-weighted averages over each agent’s neighborhood, avoiding complete mean-field approximation while maintaining computational tractability through variational message passing with Jaakkola bounds. Information gain about interpersonal trust is obtained exclusively through Chat and Exercise actions, implementing curiosity-driven social exploration as a distinct behavioural mode.
2. Robust Parameter Regime for Chaotic Itinerancy: Systematic parameter scans demonstrate that chaotic itinerancy is robust across a wide range of Hill coefficients, coupling parameters, and noise levels. This establishes that the model’s qualitative behaviour is not critically dependent on specific parameter choices.
3. Reproducible Verification Protocols: We provided complete simulation protocols, parameter tables, and verification procedures in the Supplementary Materials, with chaotic-itinerancy diagnostics as the primary test supplemented by intervention response and multiple-equilibria checks.

The key theoretical insight is that structured variability in collective social systems can be understood through the principled lens of Active Inference. By embedding Hill-type nonlinearities, trust–empowerment coupling, and precision-gated learning within agents’ generative models, we obtain chaotic itinerancy as an emergent property of EFE minimisation—without recourse to outcome-level social priors that would directly encode the collective patterns to be explained.

From a practical standpoint, the model suggests that workshop facilitation shapes not which single “attractor” the group reaches, but rather the *itinerant route* by which the group explores the space of collective possibilities. Interventions can redirect trajectories among metastable modes, but the system’s inherent variability means that outcomes are shaped by ongoing interaction rather than determined by initial conditions alone.

The chaotic-itinerancy analysis—our primary numerical contribution—demonstrates that workshop-like dynamics naturally exhibit metastable switching among multiple collective modes. Across a broad parameter set, trajectories exhibit the hallmarks of CI: multiple attractor ruins, irregular switching, and structured variability consistent with the “attractor ruin” picture [11,12,13,43]. This supports an interpretation in which workshops alternate among transient modes (inhibited, exploratory, playful, confrontational) before settling, and in which facilitation may shape the sequence of visited modes rather than determining a single outcome.

Future directions include exploring alternative network topologies (small-world, scale-free), incorporating agent heterogeneity, enabling dynamic network structure, and developing empirTical protocols for tracking latent states from behavioural data in real workshop settings.

## Figures and Tables

**Table 1:** POMDP components of the workshop model.

Component	Symbol	Description
External states	$S_{t},\left\{W_{ij,t}\right\}$	Collective trust, Interpersonal trust (partially observable)
Internal states	$\left(u_{t}^{i},H_{t}^{i}\right)$	Empowerment, stamina (fully observable)
Observation	$o_{t}^{i}$	Perceived others’ expression rate (noisy)
Action	$a_{t}^{i}\in \left\{0,1,2\right\}$	Rest/Chat and Exercise/Express
History	$h_{\leq t}^{i}$	$\left(o_{\leq t}^{i},a_{\leq t}^{i},u_{\leq t}^{i},H_{\leq t}^{i}\right)$

**Table 2:** Synchrony effect function $\phi_{\mathrm{base}}\left(a^{i},a^{j}\right)$.

$\phi_{\mathrm{base}}$	Rest (0)	Chat and Ex. (1)	Express (2)
Rest (0)	−0.5	−0.5	−1.0
Chat and Ex. (1)	−0.5	+0.5	−1.0
Express (2)	−1.0	−1.0	+1.0

**Table 3:** Network parameters and default values.

Symbol	Description	Default
$k_{\mathrm{avg}}$	Average network degree	4
$a_{W}$	Interpersonal trust decay	0.90
$b_{W}$	Interpersonal trust bias	0.00
$c_{W}$	Synchrony effect strength	0.40
$\sigma_{W}$	Interpersonal trust noise	0.10

**Table 4:** Preference learning parameters.

Symbol	Description	Value
$\Pi_{\min}$	Minimum learning precision (low trust)	0.01
$\Pi_{\max}$	Maximum learning precision (high trust)	0.5
λ	Precision modulation sharpness	4.0
$\theta_{S}$	Trust threshold for precision center	0.5
$\sigma_{\mu }$	Hyperstate drift noise	0.005
$v_{\mu }$	Prior variance of preference	0.1
$\lambda_{z}$	Mastery detection sharpness	5.0
$\theta_{\mathrm{gap}}$	Minimum gap for mastery detection	0.3

**Table 5:** Observability and EFE contributions of state variables.

Variable	Observability	Contribution to Risk	Information Gain
*S* (Collective Trust)	Partial	Belief vs. preference	Yes (Express)
$W_{ij}$ (Interpersonal Trust)	Partial	Aggregated belief vs. preference	Yes (Chat and Ex. only)
*u* (Empowerment)	Full	Prediction vs. preference	No
*H* (Stamina)	Full	Prediction vs. preference	No

**Table 6:** Chaotic itinerancy (CI) marginal results in the parameter scan (80 configurations; three trials each). “CI fraction” is the fraction of configurations classified as CI within each marginal slice; “mean indicators” is the average of the five-indicator score.

Parameter	Value	CI Fraction	Mean Indicators
$\beta_{\mathrm{action}}$	1.0	81.2%	2.69
$\beta_{\mathrm{action}}$	2.0	68.8%	2.62
$\beta_{\mathrm{action}}$	3.0	81.2%	2.77
$\beta_{\mathrm{action}}$	5.0	75.0%	2.77
$\beta_{\mathrm{action}}$	8.0	87.5%	2.79
*n*	4	85.0%	2.68
*n*	6	70.0%	2.65
*n*	8	75.0%	2.75
*n*	10	85.0%	2.83
$\sigma_{S}$	0.02	70.0%	2.63
$\sigma_{S}$	0.05	95.0%	2.93
$\sigma_{S}$	0.08	80.0%	2.72
$\sigma_{S}$	0.12	70.0%	2.63

**Table 7:** Chaotic itinerancy detection across model conditions (10 seeds). CI Pass Rate indicates the fraction of runs exhibiting chaotic itinerancy (all three primary indicators positive).

Model Condition	CI Pass Rate	Mean Indicators	Seed = 1 Result
Full Model (Nonlinear)	8/10 (80%)	2.40/3	PASS (3/3)
Linear ($c_{S,\mathrm{hill}}=0$)	2/10 (20%)	1.20/3	FAIL (2/3)
No Coupling (K=0)	0/10 (0%)	0.60/3	FAIL (1/3)

**Table 8:** Summary of parameter sensitivity analysis.

Parameter	Range	Bistable	CI Characteristics
Hill coeff. *n*	2–10	≥4	70–85% (n≥4); default 4 (85%)
Half-sat. *K*	0.2–0.8	0.27–0.70	70–90% in bistable; default 0.40 (80%)
Precision λ	2–16	All	Robust 80–90%; default 4.0
Coupling $\kappa_{S\to u}$	0.2–1.0	≥0.4	70% → 100%; default 0.6 (80%)

**Table 9:** Comparison of Baseline vs. Complete Model agents (N=6, T=400, 5 runs).

Metric	Baseline (Incomplete)	Complete Model
Expression rate $\bar{e}$	0.364±0.186	0.000±0.000
State transitions	55.2±27.9	0.0±0.0
CI Diagnosis (Primary 3 Indicators, Section 5.1)		
1. Attractor ruins	8 clusters (PASS)	3 clusters (PASS)
2. Residence time skewness	14.04 (PASS)	1.61 (PASS)
3. FTLE signature	σ=0.128 (PASS)	σ=0.027 (FAIL)
CI Classification	3/3 → CI = YES	2/3 → CI = NO

**Table 10:** Comparison of preference update mechanisms across conditions (mean ± std, n=5 runs).

Metric	Double-Gate	Single-Gate	No-Gate
Ratchet ratio	4.45±1.26	3.82±1.15	3.30±0.77
Net change ($\Delta \mu_{U}$)	0.68±0.54	0.75±0.60	0.72±0.57
Max retracement	0.15±0.12	0.15±0.12	0.14±0.12
$\mu_{U}$ variance	0.08±0.04	0.09±0.05	0.08±0.04

**Table 11:** Structural comparison of modelling approaches for collective social dynamics.

Feature	Ising-Type	Small-Group	AI Social	This Work
Action selection basis	Energy function	ODE/SDE	Social priors	EFE minimisation
Latent state inference	No	No/exogenous	Limited	EKF + VMP
Partial observability	No	No	Yes	Yes (action-dep.)
Resource constraints	No	Some	No	Stamina (endogenous)
Preference learning	No	No	No	Precision-gated
Network structure	Lattice/MF	Some	No	ER + dynamic $W_{ij}$
Chaotic itinerancy	No	Not tested	Not tested	Yes (80%)

**Table 12:** Chaotic itinerancy across network topologies (N=6, T=400, $k_{\mathrm{avg}}=4$, 10 seeds each).

Topology	CI Pass Rate	CI Indicators	Mean $\bar{e}$	Transitions	Action Entropy
Erdős–Rényi	70%	2.6	0.28±0.21	90.4	0.28
Watts–Strogatz	50%	2.5	0.36±0.17	145.1	0.37
Barabási–Albert	40%	2.3	0.25±0.23	86.1	0.23

**Table 13:** Chaotic itinerancy under agent heterogeneity at CI-maintenance thresholds (N=6, T=400, ER network, 10 seeds each).

Condition	Parameter	CV	CI %	Mean $\bar{e}$
Baseline (homogeneous)	—	—	70%	0.28±0.21
A: Preference	$\mu_{U,\mathrm{init}}$	0.08	80%	0.32±0.19
B: Decision style	$\beta_{\mathrm{action}}$	0.20	70%	0.28±0.21
C: Empowerment gain	$\eta_{\mathrm{self}}$	0.02	80%	0.36±0.17

**Table 14:** Maximum coefficient of variation (CV) that maintains chaotic itinerancy for each heterogeneity parameter.

Parameter	Max CV for CI	Sensitivity
$\beta_{\mathrm{action}}$ (decision style)	∼0.20	Robust
$\mu_{U,\mathrm{init}}$ (preference)	∼0.08	Moderate
$\eta_{\mathrm{self}}$ (empowerment gain)	∼0.02	Highly sensitive

**Table 15:** Quantitative comparison of the Active Inference model with a Brock–Durlauf Ising baseline [50] (N=6, T=400, 10 seeds).

Model	CI Pass Rate	Mean $\bar{e}$	Transitions	Action Entropy
Active Inference (this work)	70%	0.28±0.21	90.4	0.28
Brock–Durlauf Ising baseline	0%	0.44±0.02	72.7	0.82

## Data Availability

The original contributions presented in this study are included in the article/supplementary material. Further inquiries can be directed to the corresponding author.

## Supplementary Materials

The following supporting information can be downloaded at: https://www.mdpi.com/article/10.3390/e28050491/s1. Refs. [65,66,67,68] are cited in Supplementary Materials file.
