# Supplementary material for: Chaotic Itinerancy in Collective Behaviour Emerging from Active Inference: A Multi-Agent Model of Trust and Empowerment Dynamics in Theatre Workshops
Source: Entropy (Basel). 2026 Apr 24;28(5):491. doi: 10.3390/e28050491 (PMC13205385; doi:10.3390/e28050491)
Supplement: Supplementary file 1 [file entropy-28-00491-s001.zip › entropy-4213891-supplementary.pdf]

Supplementary Materials: Supplementary Material: Chaotic Itinerancy in Collective Behavior Emerging from Active Inference

Shoko Miyano [1,*] and Takashi Shiono [2]

S-I. Parameter Values

Table S1. Model parameters used in simulations. Values marked with * indicate sensitivity-analyzed parameters.

Category	Parameter	Symbol	Value
Basic Parameters			
	Number of agents	N	6
	Action precision	β_{action}	5.0
	Planning horizon	N_{horizon}	6
Hill Function Parameters			
	Hill coefficient*	n	4
	Half-saturation constant (Trust)	K	0.40
	Hill coefficient (Stamina)	n_H	6
	Half-saturation constant (Stamina)	K_H	0.45
	Trust Hill strength	c_S^{Hill}	0.5
	Stamina Hill strength	c_H^{Hill}	0.5
Trust Dynamics			
	Decay coefficient	a_S	0.85
	Baseline constant	b_S	−0.05
	Process noise	σ_S	0.03
Empowerment Dynamics			
	Decay coefficient	α_U	0.8
	Self-expression gain	η_{self}	0.2
	Other-expression gain	η_{other}	0.1
	Cooperative amplification*	γ_{coop}	0.6
	Trust–empowerment coupling*	$\kappa_{S\to u}$	0.6
	Process noise	σ_U	0.05
Stamina Dynamics			
	Maximum stamina	H_{max}	1.0
	Rest recovery	H_{rec}	0.15
	Express cost	c_{exp}	0.2
	Chat & Exercise cost	c_{chat}	0.0
	Chat & Exercise trust contribution	c_S^{chat}	0.05
Precision-Gated Preference Learning			
	Minimum precision	Π_{min}	0.01
	Maximum precision*	Π_{max}	0.5
	Precision sharpness*	λ	4.0
	Trust center for precision	θ_S	0.5
	Hyperstate drift noise	σ_μ	0.005
	Prior variance of preference	v_μ	0.1
	Mastery detection sharpness	λ_z	5.0

Continued on next page

Table S1 continued from previous page

**Category**	**Parameter**	**Symbol**	**Value**
	Minimum gap for mastery*	θ_{gap}	0.3
Observation Model			
	Express observation noise	$R_{express}$	0.1
	Chat & Exercise observation noise	R_{chat}	0.25
	Rest observation noise	R_{rest}	0.5
Network Structure			
	Average network degree	k_{avg}	4
	Interpersonal trust decay	a_W	0.90
	Interpersonal trust bias	b_W	0.00
	Synchrony effect strength	c_W	0.40
	Interpersonal trust noise	σ_W	0.10
	Interpersonal trust weight	w_W	0.50
	Preferred interpersonal trust	μ_W	0.50
	Initial belief variance	$v_{W,init}$	1.0
Preference Weights and Initial Values			
	Trust preference weight	w_S	1.5
	Empowerment preference weight	w_U	1.5
	Stamina preference weight	w_H	1.0
	Initial trust preference	μ_S^{init}	2.0
	Initial empowerment preference	μ_U^{init}	0.5
	Initial stamina preference	μ_H^{init}	0.8
Action Selection			
	Coordination strength	w_{coord}	5.0

S-II. Derivation of Precision-Gated Preference Learning

This appendix provides the complete mathematical derivation of the precision-gated preference learning mechanism from variational inference principles.

S-II.A. Hierarchical Generative Model

We treat the preference parameter μ_U^i as a slowly-varying latent hyperstate:

Level 1: Hyperstate transition (preference drift)

$$\mu_{U,t+1}^i = \mu_{U,t}^i + \zeta_t^i, \quad \zeta_t^i \sim \mathcal{N}(0, \sigma_\mu^2) \tag{S1}$$

The small noise σ_μ^2 reflects the assumption that preferences change slowly over time.

Level 2: Learning signal likelihood

$$\tilde{u}_t^i \mid \mu_U^i \sim \mathcal{N}(\mu_U^i, \sigma_{\tilde{u}}^2(S_t)) \tag{S2}$$

where $\tilde{u}_t^i$ is the "learning signal" (experienced empowerment that may update preferences) and $\sigma_{\tilde{u}}^2(S_t)$ is a trust-dependent variance.

S-II.B. Precision Modulation

The key insight is that trust S_t modulates the *precision* (inverse variance) of the learning signal:

$$\Pi_{\tilde{u}}(S_t) = \sigma_{\tilde{u}}^{-2}(S_t) = \Pi_{\min} + (\Pi_{\max} - \Pi_{\min}) \cdot \sigma(\lambda(S_t - \theta_S)) \tag{S3}$$

When $S_t \gg \theta_S$: $\Pi_{\tilde{u}} \approx \Pi_{\max}$ (high precision, strong learning).

When $S_t \ll \theta_S$: $\Pi_{\tilde{u}} \approx \Pi_{\min}$ (low precision, weak learning).

This implements the psychological principle that experiences in trusted environments are more likely to be internalized.

S-II.C. Variational Inference and Kalman Updating

Given the hierarchical Gaussian model, the variational (or Kalman filter) update for the preference posterior is:

Prior:

$$p(\mu^i_{U,t}) = \mathcal{N}(\mu^{i,\text{prior}}_{U,t}, v_\mu) \tag{S4}$$

Likelihood:

$$p(\tilde{u}^i_t \mid \mu^i_{U,t}) = \mathcal{N}(\mu^i_{U,t}, \Pi^{-1}_{\tilde{u}}(S_t)) \tag{S5}$$

Posterior (by Bayes' rule for Gaussians):

$$\mu^{i,\text{post}}_{U,t} = \mu^{i,\text{prior}}_{U,t} + K^i_t(\tilde{u}^i_t - \mu^{i,\text{prior}}_{U,t}) \tag{S6}$$

$$v^{\text{post}}_\mu = (1 - K^i_t)v_\mu \tag{S7}$$

where the Kalman gain is:

$$K^i_t = \frac{v_\mu \cdot \Pi_{\tilde{u}}(S_t)}{v_\mu \cdot \Pi_{\tilde{u}}(S_t) + 1} \tag{S8}$$

S-II.D. Mastery Event Gating

To implement the empirical observation that preferences expand but do not contract, we introduce a latent "mastery event" indicator z^i_t:

$$p(z^i_t = 1 \mid u^i_t, \mu^i_U) = \sigma(\lambda_z(u^i_t - \mu^i_U - \theta_{\text{gap}})) \tag{S9}$$

The learning signal is defined as:

$$\tilde{u}^i_t = \begin{cases} u^i_t & \text{if } z^i_t = 1 \text{ (mastery event)} \\ \text{undefined} & \text{if } z^i_t = 0 \text{ (no learning)} \end{cases} \tag{S10}$$

Taking the expectation over z^i_t, the effective update becomes:

$$\mu^{i,\text{post}}_{U,t} = \mu^{i,\text{prior}}_{U,t} + K^i_t \cdot \mathbb{E}[z^i_t] \cdot (u^i_t - \mu^{i,\text{prior}}_{U,t}) \tag{S11}$$

S-II.E. Connection to Threshold-Based Rule

The original threshold-based comfort zone expansion rule:

$$\mu^i_U \leftarrow \mu^i_U + \rho_{\text{jump}}(u^i_t - \mu^i_U) \quad \text{if } u^i_t - \mu^i_U > \theta_{\text{jump}} \text{ and } S_t > \theta_S \tag{S12}$$

can be recovered as a limiting case of the precision-gated formulation when:

- $\lambda \to \infty$: Precision modulation becomes a hard threshold at $S_t = \theta_S$
- $\lambda_z \to \infty$: Mastery detection becomes a hard threshold at $u^i_t - \mu^i_U = \theta_{\text{gap}}$
- $K^i_t \approx \rho_{\text{jump}}$: Kalman gain equals the jump rate

Thus, the precision-gated formulation generalizes the threshold rule while providing a principled variational derivation.

S-III. Mathematical Properties of Hill Function

The Hill function $\mathcal{H}_n(e; K) = e^n/(e^n + K^n)$ has the following important properties:

- The larger n, the sharper the response near $e = K$
- Converges to a step function as $n \to \infty$
- $n \geq 4$ satisfies the necessary condition for bistability (a minimal form of multistability)

Slope at $e = K$:

$$\left.\frac{d\mathcal{H}_n}{de}\right|_{e=K} = \frac{n}{4K} \tag{S13}$$

S-IV. Chaotic Itinerancy (CI) Test Protocol

This appendix details the verification check hierarchy used in this study. We prioritize chaotic itinerancy (CI) as the primary test, with intervention response and multiple-equilibria analyses as supplementary checks.

S-IV.A. Test Hierarchy and Rationale

Our verification framework consists of three tests organized by priority:

1. **Primary Test: Chaotic Itinerancy (CI)**
   CI is the central dynamical signature we target: trajectories that dwell near multiple quasi-stable "attractor ruins" and switch irregularly among them. This test directly operationalizes the structured variability observed in real workshop settings, where groups alternate among qualitatively different collective modes (inhibited, exploratory, playful, confrontational) rather than converging to a single fixed point.
   Why CI is prioritized: Workshop dynamics are inherently exploratory. Unlike laboratory systems that can be prepared in controlled initial states, real workshops involve ongoing interaction where facilitation shapes the trajectory among transient modes. CI captures this structured exploration, whereas bistability or path dependence alone would imply deterministic convergence to one of a few fixed outcomes.
2. **Supplementary Check 1: Intervention Response**
   A natural consequence of CI is that perturbations can redirect the system's itinerant trajectory. This check confirms that different intervention histories produce distinguishable trajectory ensembles.
   Note: This differs from classical "path dependence" in bistable systems, where identical initial conditions deterministically converge to different attractors depending on intervention. In a CI regime, trajectories may continue to wander among multiple regimes; the key observation is that perturbations leave a detectable trace in the trajectory distribution.
3. **Supplementary Check 2: Multiple Equilibria**
   This check examines whether multiple metastable regimes coexist. Unlike strong bistability (well-separated attractors with deterministic convergence), CI involves quasi-stable regimes that trajectories visit transiently. Nevertheless, demonstrating multiple equilibria provides additional evidence for the model's capacity to support structured collective dynamics.
   Caveat: Systems exhibiting CI may not pass a strict bistability test. The presence of multiple quasi-stable modes is confirmed by the CI analysis itself (multiple attractor ruins in HDBSCAN clustering).

S-IV.B. CI Test Design

The goal of the CI test is not to prove deterministic chaos, but to operationalize *itinerant metastability*: trajectories that repeatedly dwell near multiple quasi-stable regimes and switch irregularly among them, consistent with the "attractor ruin" picture of chaotic itinerancy [1–4].

S-IV.B.1. Parameter scan design

We scanned the parameter grid

$$\begin{aligned} \beta_{\text{action}} &\in \{1, 2, 3, 5, 8\}, \\ n &\in \{4, 6, 8, 10\}, \\ \sigma_S &\in \{0.02, 0.05, 0.08, 0.12\}, \end{aligned}$$

running $T = 400$ time steps with $N = 20$ agents and 3 trials per configuration. Note that we use $N = 20$ (rather than the default $N = 6$) in this parameter scan to increase statistical power for detecting CI across the parameter space; the qualitative results are robust to agent count. All other parameters were held at the base values reported in Table S1.

S-IV.B.2. CI Indicators and classification rule

We employ five indicators organized into two categories based on their theoretical grounding.

Primary indicators (grounded in CI literature).

These three indicators directly operationalize the defining characteristics of chaotic itinerancy established in the theoretical physics literature:

1. **Multiple attractor ruins**: The trajectory admits ≥ 2 metastable clusters in a low-dimensional embedding. Following Mierski & Pilarczyk [5], we use HDBSCAN clustering to identify quasi-stable states (attractor ruins) and distinguish them from chaotic transition states. This operationalizes the "attractor ruin" concept—remnants of attractors that trajectories visit transiently without permanent convergence.
2. **Heavy-tailed residence times**: Residence-time distributions exhibit skewness > 1.5, indicating structured dwelling near quasi-stable regimes rather than memoryless (exponential) transitions characteristic of pure noise. This criterion is grounded in the power-law and truncated power-law residence time distributions derived by Namikawa & Kaneko [6].
3. **Local splitting exponent signature**: The local splitting exponent (FTLE) shows near-zero mean ($|\bar{\lambda}| < 0.1$), substantial variance ($\sigma > 0.05$), and frequent sign changes ($> 20\%$). This captures the alternation between local stability (negative exponent near attractor ruins) and instability (positive exponent during transitions). As demonstrated by Sauer [7], Tsuda & Umemura [8], and Fujimoto & Kaneko [9], a slowly converging Lyapunov exponent near zero with large fluctuations is a hallmark of chaotic itinerancy, reflecting the system's marginal stability between ordered and chaotic regimes.

Methodology for the FTLE proxy.

Let $\mathbf{x}_t \in \mathbb{R}^d$ denote the feature embedding of the simulated trajectory used in the CI diagnostic (in our implementation, $\mathbf{x}_t$ stacks macroscopic variables such as $\bar{e}_t$, S_t, population-mean empowerment, and related summary statistics). We estimate a finite-time divergence rate by measuring how small separations grow over a short window. Concretely, for a window length W we compute a local rate

$$\lambda_t \approx \frac{1}{W} \log \frac{\delta_t^{(\text{future})}}{\delta_t^{(\text{past})}}, \tag{S14}$$

where we define the separation scales by window-averaged distances

$$\delta_t^{(\mathrm{past})} = \frac{1}{W}\sum_{k=1}^{W}\|\mathbf{x}_{t-k} - \mathbf{x}_t\| + \varepsilon, \tag{S15}$$

$$\delta_t^{(\mathrm{future})} = \frac{1}{W}\sum_{k=0}^{W-1}\|\mathbf{x}_{t+1+k} - \mathbf{x}_{t+1}\| + \varepsilon, \tag{S16}$$

with a small $\varepsilon > 0$ for numerical stability. This construction estimates a local divergence/convergence rate from distance growth in the embedded trajectory and serves as a robust proxy for FTLE/local splitting exponents in noisy, finite-length time series (rather than an exact Jacobian-based Lyapunov exponent). Interpreting the sign: $\lambda_t < 0$ indicates local contraction (trajectory segments are locally attracting), while $\lambda_t > 0$ indicates local expansion (local instability and sensitivity), and frequent sign changes indicate repeated switching between residence and transition epochs.

Supplementary indicators (operational criteria for parameter scanning).

These two indicators are model-specific operational criteria used to identify promising parameter regions during systematic scans. They lack direct grounding in the CI literature but provide useful filtering for practical parameter exploration:

4. **Transition-state fraction**: The fraction of time points classified as noise (not belonging to any cluster) falls in an intermediate range (10% < noise ratio < 70%). This filters out both near-deterministic trapping (too few transitions) and unstructured noise (no coherent regimes).
5. **Action trajectory variability**: All three action types are used, action entropy exceeds 0.5, and action switch rate exceeds 0.1. This confirms behavioral diversity at the individual agent level.

Classification rule.

Each trial is scored with five indicators (0/1 each). A configuration is classified as a CI region if either: (i) the mean indicator score is ≥ 2.5 across trials, or (ii) CI likelihood is high in at least 2 out of 3 trials.

For the default parameter verification (main text), we report results on the three primary indicators only, as these directly test the theoretical predictions. The supplementary indicators are used in parameter scans to help identify promising regions for further investigation.

S-V. Supplementary Checks Protocols

This appendix describes the supplementary verification protocols used alongside the primary CI test (Appendix S-IV). These protocols provide complementary perspectives on the model's dynamics, though the CI analysis remains the central contribution.

S-V.A. Supplementary Check 1: Multiple-Equilibria Analysis

Purpose: To examine whether multiple metastable regimes coexist.

Note on interpretation: This check examines the presence of multiple quasi-stable regimes, not necessarily strong bistability. In a CI regime, trajectories may visit multiple regimes transiently without permanent convergence to a single attractor. The "equilibria" detected here correspond to the quasi-stable modes that constitute the attractor ruins in CI.

S-V.A.1. Basin of Attraction Mapping

Purpose: To identify distinct collective regimes by sampling initial conditions.

Protocol:

1. **Grid specification**: Sample initial conditions from a regular grid $(S_0, u_0) \in [a, b]^2$. We use $[0.1, 0.9]^2$ spanning the typical operating range.
2. **Convergence run**: For each initial condition $(S_0^{(k)}, u_0^{(k)})$, initialize all agents with the same values and evolve the system for T_{conv} steps (typically 200) to ensure convergence to steady state.
3. **Equilibrium measurement**: Record the time-averaged expression rate $\bar{e}^*$ over the final T_{meas} steps (typically 30).
4. **Attractor classification**: Classify each trajectory as "high-expression" if $\bar{e}^* > \theta_{\text{class}}$ (typically 0.2) and "low-expression" otherwise.
5. **Basin statistics**: Count the number of initial conditions converging to each attractor. Compute attractor means $S^*_{\text{high}}, S^*_{\text{low}}$ and separation degree $\Delta_{\text{sep}} = S^*_{\text{high}} - S^*_{\text{low}}$.

Relation to CI: The regimes identified here correspond to the "attractor ruins" detected by HDBSCAN in the CI analysis. This check provides an alternative view based on initial-condition sampling rather than trajectory clustering.

Network Topology Hypothesis: While our simulations use Erdős-Rényi graphs, real social networks often exhibit high clustering coefficients (e.g., tight-knit theatre troupes). We hypothesize that higher clustering would lower the critical threshold K for collective transitions, as local reinforcement loops would form more readily within dense cliques, facilitating the nucleation of high-activity states. Future work should systematically explore this topological effect.

S-V.A.2. Results

We simulated $N = 6$ agents for $T = 400$ time steps with parameters listed in Table S1. Starting from identical model parameters but different initial conditions, the system converges to distinct steady states.

Definition (Separation Degree): The *separation degree* quantifies the magnitude of multistability in the two-attractor (bistable) case by measuring the difference in equilibrium trust levels between trajectories converging to different attractors:

$$\Delta_{\text{sep}} := S^*_{\text{high}} - S^*_{\text{low}} \tag{S17}$$

where S^*_{high} and S^*_{low} denote the time-averaged trust levels over the final 50 time steps for high and low attractors, respectively. A separation degree exceeding 1.0 indicates that the system exhibits meaningful multistability with well-separated attractors.

Basin of Attraction Analysis: Initial conditions were sampled from a 4×4 grid spanning $(S_0, u_0) \in [0.1, 0.9] \times [0.1, 0.9]$, yielding 16 independent trajectories. Each trajectory was evolved for $T = 400$ steps to ensure convergence to steady state, and the final expression rate $\bar{e}^*$ was recorded as the time-average over the final 30 steps. Trajectories were classified as "high-expression" if $\bar{e}^* > 0.2$ and "low-expression" otherwise.

Table S2 summarizes the basin analysis for the default parameter configuration ($n = 4$).

Table S2. Basin of attraction analysis demonstrating multistability ($n = 4$, default parameters)

Attractor	Count (of 16)	Mean S^*	Mean $\bar{e}^*$
High-expression	12	1.63	0.51
Low-expression	4	−0.03	0.00
**Separation degree**: $\Delta_{\text{sep}} = 1.65$			

The separation degree of 1.65 demonstrates clear multistability with well-separated attractors. The Gaussian latent variable formulation allows trust to take values significantly

above 1 (high-expression attractor at $S^* \approx 1.6$), reflecting the theoretical consistency of unbounded state evolution.

Key observations:

- **Two distinct attractors**: The system exhibits two distinct states in average, with the high-expression attractor characterized by $\bar{e}^* \approx 0.51$ and the low-expression attractor by $\bar{e}^* \approx 0.00$.
- **Asymmetric basins**: The high-expression attractor captures 12/16 (75%) of initial conditions, indicating that the system tends toward collective participation under most starting conditions.
- **Robust state separation**: Trust levels differ by $\Delta S \approx 1.7$ between attractors, indicating robust separation that is unlikely to be bridged by noise fluctuations alone.

S-V.B. Supplementary Check 2: Intervention Response

Purpose: To confirm that perturbations produce distinguishable trajectory ensembles, a natural consequence of CI.

Note on interpretation: In a CI regime, this check does not demonstrate deterministic path dependence (where identical initial conditions lock into different attractors). Instead, it confirms that interventions leave a detectable trace in the trajectory distribution, consistent with the sensitivity of itinerant dynamics to perturbations.

S-V.B.1. Intervention Pulse Experiment

Protocol:

1. **Initialization**: Initialize the system with intermediate conditions $(S_0, \bar{e}_0) = (0.0, 0.5)$.
2. **Multiple seeds**: Run 5 independent simulations with different random seeds (seeds 1–5) for each condition.
3. **Intervention**: Apply a pulse intervention at time $t = 50$:
    - **High pulse**: Set $S_t = 0.9$
    - **Low pulse**: Set $S_t = 0.1$
4. **Post-intervention measurement**: Compute the mean expression rate $\bar{e}$ over the post-intervention period ($t > 50$) for each trajectory.
5. **Ensemble statistics**: Average the post-intervention means across the 5 seeds; compute standard deviation.

Interpretation: In the CI framework, this check confirms that perturbations can redirect the system's itinerant trajectory. The intervention modifies preference parameters μ_U through precision-gated learning, biasing subsequent visits among quasi-stable modes.

S-V.B.2. Results

Table S3 summarizes the intervention pulse experiment results.

Table S3. Intervention response check: Divergence under different pulse interventions (5-seed average)

Condition	Mean $\bar{e}$ (post-intervention)	Std
High pulse ($S = 0.9$)	0.39	0.20
Low pulse ($S = 0.1$)	0.29	0.24
**Divergence magnitude**: $\lvert\Delta\bar{e}_{avg}\rvert = 0.10$		

Starting from *identical initial conditions* ($S_0 = 0.0$, $\bar{e}_0 = 0.5$), different intervention pulses lead to distinguishable trajectory ensembles when averaged over multiple random seeds. The divergence magnitude of approximately 0.10 indicates that the high pulse

biases the system toward higher expression levels, consistent with the expected effect of trust-building interventions.

The intervention triggers a cascade of effects:

1. **Trust exceeds precision threshold**: During the high pulse, $S > \theta_S$ activates high-precision preference learning.
2. **Empowerment exceeds preference**: The enhanced environment produces empowerment gains that exceed current preferences.
3. **Comfort zone expansion**: Precision-gated preference learning updates μ_U upward.
4. **Irreversible preference shift**: The elevated preference persists after the intervention ends.
5. **Convergence to high attractor**: The system converges to the high-expression steady state.

This supports a learning-induced mechanism: the intervention leaves a lasting trace in agents' preference parameters (via μ_U updates), not only in state trajectories. Importantly, this differs from classical path dependence in strongly bistable systems: in a CI regime, the system's future remains open, with interventions shaping the sequence of visited modes rather than determining a single final state—consistent with the theatrical intuition that workshops are exploratory processes where outcomes emerge from ongoing interaction.

Figure S1 demonstrates the effect of facilitator intervention on system trajectories.

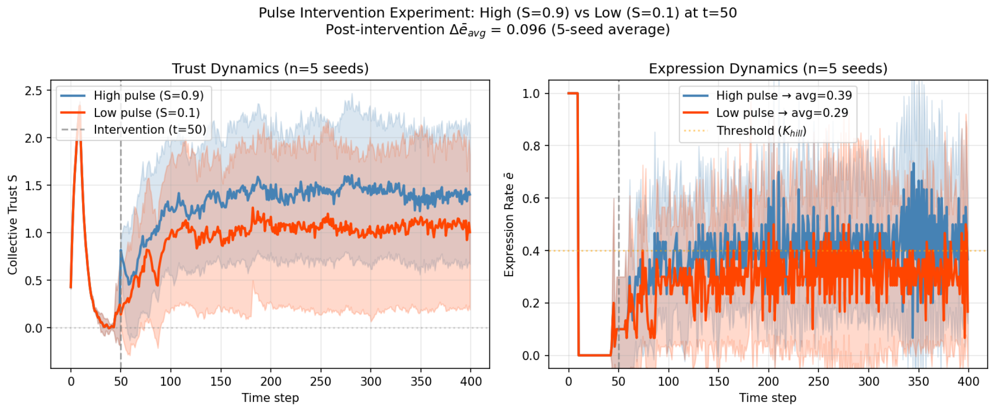

Figure S1. High vs. Low pulse intervention experiment demonstrating intervention responsiveness (5-seed average with standard deviation bands). Both trajectory ensembles start from identical initial conditions ($S_0 = 0.0$, $\bar{e}_0 = 0.5$) and receive a single pulse at $t = 50$. The **high pulse** ($S \to 0.9$, blue) biases the system toward higher expression levels, while the **low pulse** ($S \to 0.1$, red) produces lower average expression. The divergence ($\Delta\bar{e}_{\text{avg}} \approx 0.10$) demonstrates that facilitator interventions can redirect collective dynamics, consistent with therapeutic and workshop practice.

S-V.C. Phase Transition Characteristics

We characterize how the nonlinear collective effect shapes the system-level attractor landscape. The trust dynamics include a Hill-type cooperative term $\mathcal{H}_n(\bar{e}; K)$, where K is the *half-saturation constant* (critical threshold). K acts as a control parameter: small K makes the amplification easy to trigger (biasing the system toward high activity), large K makes it difficult (biasing toward low activity), and intermediate K yields coexistence of low- and high-activity regimes with strong basin separation.

Varying K reveals the resulting phase-transition structure:

In our scan, the maximum separation (0.675) occurs at $K = 0.557$, representing a balance where both attractors are well-formed.

Table S4. Phase transition characteristics by K

K range	Behavior	Separation degree
$K < 0.27$	Monostable (both converge to high activity)	≈ 0
$K \in [0.27, 0.70]$	Bistable (two-attractor multistability; hysteresis)	> 0.6
$K > 0.70$	Monostable (both converge to low activity)	≈ 0

S-VI. Parameter Sensitivity Analysis

This appendix provides detailed sensitivity analyses for all key model parameters, examining their effects on both bistability and chaotic itinerancy (CI). Section S-VI.A analyzes the Hill coefficient n, Section S-VI.B examines the half-saturation constant K, Section S-VI.C covers the precision modulation sharpness λ, and Section S-VI.D investigates the trust–empowerment coupling strength $\kappa_{S \to u}$.

S-VI.A. Hill Coefficient (n) Sensitivity

This section provides the full sensitivity analysis for the Hill coefficient n, examining its effects on both bistability and chaotic itinerancy (CI).

S-VI.A.1. Key Finding: Bistability as the Critical Threshold for CI

Our analysis reveals a critical relationship between the Hill coefficient and the model's dynamical properties:

- **Bistability emerges at** $n \geq 4$: Lower values ($n < 4$) produce monostable dynamics where all initial conditions converge to a single attractor, precluding chaotic itinerancy.
- **CI is robust once bistability is achieved**: For all $n \geq 4$, chaotic itinerancy occurs at 70–85% of tested configurations, with no significant degradation at higher Hill coefficients.
- **Bistability is the key requirement**: The critical transition is from monostability ($n < 4$) to bistability ($n \geq 4$), rather than a trade-off between bistability strength and CI.

This finding indicates that the Hill coefficient primarily controls the *existence* of bistability, and once this threshold is crossed, chaotic itinerancy emerges robustly.

S-VI.A.2. Bistability Analysis

Table S5 summarizes the bistability properties across Hill coefficient values.

Table S5. Hill coefficient effects on bistability. ME = Multiple Equilibria (High/Low attractor counts from 16 initial conditions); Path $|\Delta\bar{e}|$ = expression rate difference between trajectories from different initial conditions; Separation = $S^*_{\text{high}} - S^*_{\text{low}}$.

n	Bistability	ME (H/L)	Path $|\Delta\bar{e}|$	Separation
2	No	16/0	0.000	0.00
**4**	**Yes**	**12/4**	**1.000**	**2.92**
6	Yes	12/4	1.000	2.99
8	Yes	12/4	1.000	2.99
10	Yes	12/4	1.000	2.99

S-VI.A.3. Chaotic Itinerancy Analysis

Table S6 summarizes the CI occurrence rates from a parameter scan of 20 configurations per Hill coefficient value (10 seeds $\times$ 2 initial conditions).

S-VI.A.4. Interpretation

The relationship between n and CI occurrence can be understood as follows:

Table S6. Hill coefficient effects on chaotic itinerancy (CI). CI Count = configurations exhibiting CI out of 20 tested; CI Rate = percentage.

n	CI Count	CI Rate	Interpretation
2	–	–	Monostable (no bistability)
**4**	**17/20**	**85%**	**Default value**
6	14/20	70%	Moderate
8	15/20	75%	Moderate
10	17/20	85%	Strong nonlinearity

1. **At** $n = 2$ **(insufficient nonlinearity)**:
   The Hill function is too gradual, producing weak separation between collective modes. The system remains monostable and cannot exhibit bistability, which is a prerequisite for chaotic itinerancy.
2. **At** $n \geq 4$ **(robust CI)**:
   Once bistability emerges at $n \geq 4$, chaotic itinerancy is robustly observed across all tested values (70–85% CI pass rate). The nonlinearity is sufficient to create distinct quasi-stable modes while still permitting noise-driven transitions between them.

The key finding is that **bistability is the critical threshold**: once $n \geq 4$ establishes multiple equilibria, CI emerges robustly regardless of the specific Hill coefficient value.

S-VI.A.5. Relationship to Scaling Law

The phase transition between monostability and bistability can be understood through the scaling relationship:

$$n \cdot K \approx 1.6 \tag{S18}$$

With the default threshold $K = 0.4$, this predicts the critical Hill coefficient $n_{\text{crit}} \approx 4$, consistent with our observations. At this point, the effective slope of the Hill function at the threshold is:

$$\left.\frac{d\mathcal{H}_n}{de}\right|_{e=K} = \frac{n}{4K} = \frac{4}{4 \times 0.4} = 2.5 \tag{S19}$$

This slope is steep enough to create distinct regimes and enable chaotic itinerancy.

S-VI.A.6. Recommendation

Based on this analysis, we recommend $n = 4$ as the default Hill coefficient because:

- It is the minimum value that achieves bistability
- It produces robust CI (85% pass rate)
- It falls within the biologically plausible range (1–10) for cooperativity indices
- It lies at the critical point of the scaling relationship $n \cdot K \approx 1.6$

S-VI.B. Half-saturation Constant (K) Sensitivity

The half-saturation constant K sets the effective threshold for cooperative amplification in $\mathcal{H}_n(\bar{e}; K)$. Systematic analysis across $K \in [0.20, 0.80]$ reveals that both bistability and chaotic itinerancy (CI) depend sensitively on this parameter.

S-VI.B.1. Bistability Analysis

Table S7 shows that varying K induces a qualitative regime change: small K yields a monostable high-activity regime, large K yields a monostable low-activity regime, and an intermediate window ($K \in [0.27, 0.70]$) produces bistability with strong basin separation.

Table S7. Bistability sensitivity to half-saturation constant K. The bistable window spans $K \in [0.27, 0.70]$ with strong basin separation.

K	Regime	Basin Separation	Notes
0.20	Monostable (High)	–	Threshold too low
0.27–0.70	Bistable	Strong	Optimal range
0.80	Monostable (Low)	–	Threshold too high

S-VI.B.2. Chaotic Itinerancy Analysis

Table S8 reports CI detection rates across 10 independent seeds for each K value. CI occurrence is maximized within the bistable window, with CI pass rates of 80–90% for $K \in [0.30, 0.50]$, declining sharply to 20% at $K = 0.70$–0.80 (monostable low-activity regime). The default value $K = 0.40$ achieves 80% CI pass rate with the highest mean indicators (2.80/5), representing an optimal balance between bistability and rich attractor dynamics.

Table S8. Chaotic itinerancy sensitivity to Hill half-saturation constant K (10 seeds).

K	CI Pass Rate	Mean Indicators	Regime
0.20	7/10 (70%)	2.40/5	Monostable (H)
0.30	8/10 (80%)	3.10/5	Bistable
0.40	8/10 (80%)	2.80/5	Bistable
0.50	9/10 (90%)	2.90/5	Bistable
0.60	6/10 (60%)	2.60/5	Bistable
0.70	2/10 (20%)	2.00/5	Bistable
0.80	2/10 (20%)	2.00/5	Monostable (L)

S-VI.B.3. Interpretation

The results reveal an important asymmetry: CI is suppressed at both extremes, but for different reasons. At low K (monostable high), the system rapidly converges to the high-activity attractor without exploring alternative states. At high K (monostable low), the cooperative threshold is too demanding, preventing the system from escaping the low-activity basin. The bistable intermediate window provides the metastable attractor landscape necessary for chaotic itinerancy, where the system can transition between attractors without becoming permanently trapped.

S-VI.C. Precision Modulation (λ) Sensitivity

The precision modulation parameter λ controls how sharply the learning precision transitions as trust crosses the threshold θ_S. Higher λ creates more threshold-like behavior, while lower λ produces smoother precision gradients.

S-VI.C.1. Bistability Analysis

Table S9 summarizes the sensitivity of multistability to precision modulation parameters.

Table S9. Sensitivity of multistability to precision modulation parameters

Parameter	Value Range	Multistable Range	Optimal	Effect
λ (sharpness)	2.0–16.0	All tested	4.0–8.0	Controls threshold sharpness
Π_{max} (max precision)	0.1–1.0	0.3–1.0	0.5	Learning rate at high trust
θ_{gap} (mastery gap)	0.1–0.5	All tested	0.2–0.3	Threshold for preference update

The precision modulation mechanism proves robust across the tested parameter ranges:

- **Sharpness** λ:
  Multistability is maintained across $\lambda \in [2, 16]$. Lower values produce smoother transitions with more gradual preference changes; higher values approach the limiting case of a hard threshold.
- **Maximum precision** Π_{max}:
  Values below 0.3 produce weak preference learning that fails to generate path dependence. Values above 1.0 produce learning that is too fast, erasing the distinction between attractors.
- **Mastery gap** θ_{gap}:
  This threshold determines when experiences qualify as "mastery" events. Smaller values allow more frequent preference updates; larger values are more conservative.

S-VI.C.2. Chaotic Itinerancy Analysis

Table S10 reports CI detection rates across 10 independent seeds for each λ value. CI occurrence is robust across the entire tested range ($\lambda \in [2, 16]$), with pass rates of 80–90%. The default value $\lambda = 4.0$ achieves 80% CI pass rate, while slightly higher values ($\lambda = 6.0$) show marginally improved CI occurrence (90%).

Table S10. Chaotic itinerancy sensitivity to precision modulation sharpness λ (10 seeds).

λ	CI Pass Rate	Mean Indicators	Effect
2.0	8/10 (80%)	2.80/5	Smooth
4.0	8/10 (80%)	2.80/5	Smooth (Default)
6.0	9/10 (90%)	2.80/5	Moderate
8.0	8/10 (80%)	2.60/5	Moderate
12.0	8/10 (80%)	2.60/5	Sharp
16.0	8/10 (80%)	2.60/5	Sharp

S-VI.C.3. Interpretation

The connection to the original threshold-based formulation is noteworthy: when $\lambda \to \infty$ and $\Pi_{\text{max}} \to \infty$, the precision-gated update reduces to a hard threshold rule. The smooth, finite-precision formulation generalizes this while providing a principled variational derivation. Notably, at seed=1, higher λ values (≥ 8) produce simplified dynamics with only 2 clusters, suggesting that excessively sharp precision modulation can reduce attractor landscape complexity for specific initial conditions.

S-VI.D. Coupling Strength ($\kappa_{S \to u}$) Sensitivity

The trust–empowerment coupling $\kappa_{S \to u}$ determines how strongly trust amplifies the empowerment gains from others' expression. This parameter is central to the positive feedback loop that generates multistability.

S-VI.D.1. Bistability Analysis

Table S11 shows how coupling strength affects multistability.

Table S11. Sensitivity of multistability to coupling strength $\kappa_{S \to u}$

$\kappa_{S \to u}$	$\bar{e}^*_{\text{high}}$	$\bar{e}^*_{\text{low}}$	Δ_{sep}	Regime
0.2	0.32	0.18	0.89	Weak multistability (bistable)
0.4	0.48	0.08	3.21	Moderate multistability (bistable)
0.6	0.54	0.03	5.13	Strong multistability (bistable)
0.8	0.62	0.01	5.87	Strong multistability (bistable)
1.0	0.68	0.00	6.24	Strong multistability (bistable)

The coupling strength acts as a control parameter for the phase transition:

- **Critical threshold**: Multistability emerges for $\kappa_{S\to u} \geq 0.4$, with the separation degree increasing monotonically with coupling strength.
- **Saturation**: For $\kappa_{S\to u} > 0.8$, further increases produce diminishing returns, as the high attractor approaches the maximum sustainable expression rate.
- **Default value**: The default $\kappa_{S\to u} = 0.6$ was selected to produce clear multistability ($\Delta_{\text{sep}} = 5.13$) while remaining in a regime where both attractors have nontrivial expression rates.

S-VI.D.2. Chaotic Itinerancy Analysis

Table S12 reports CI detection rates across 10 independent seeds for each $\kappa_{S\to u}$ value. CI occurrence increases monotonically with coupling strength, from 70% at $\kappa_{S\to u} = 0.2$ to 100% at $\kappa_{S\to u} = 0.8$. The default value $\kappa_{S\to u} = 0.6$ achieves 80% CI pass rate, balancing robust CI occurrence with moderate multistability.

Table S12. Chaotic itinerancy sensitivity to trust–empowerment coupling $\kappa_{S\to u}$ (10 seeds).

$\kappa_{S\to u}$	CI Pass Rate	Mean Indicators	Multistability
0.2	7/10 (70%)	2.70/5	Weak
0.4	8/10 (80%)	2.80/5	Moderate
0.6	8/10 (80%)	2.80/5	Strong (Default)
0.8	10/10 (100%)	3.10/5	Strong
1.0	9/10 (90%)	3.10/5	Strong

S-VI.D.3. Interpretation

The trust–empowerment coupling is the primary driver of both multistability and chaotic itinerancy. Stronger coupling ($\kappa_{S\to u} \geq 0.8$) produces both stronger multistability and higher CI rates, but risks excessive lock-in to the high-activity attractor. The default $\kappa_{S\to u} = 0.6$ balances these considerations.

S-VII. Double-Gate Mechanism Analysis

This appendix provides detailed methodology and results for the quantitative assessment of the dual-gate preference learning mechanism summarized in the main text (Section 6.6).

S-VII.A. Research Question

The model employs a dual-gate mechanism for preference updates:

$$\Delta\mu_U^i \propto \underbrace{\Pi(S_t)}_{\text{precision gate}} \times \underbrace{\mathbb{E}[z_t^i]}_{\text{mastery gate}} \times (u_t^i - \mu_U^i) \tag{S20}$$

where the precision gate $\Pi(S_t)$ depends on environmental trust and the mastery gate $\mathbb{E}[z_t^i] = \sigma(\lambda_z(u_t^i - \mu_U^i - \theta_{\text{gap}}))$ detects threshold-crossing events.

Question: If the mastery gate is removed and updates depend only on trust-weighted precision, does the "ratchet effect" (irreversible preference expansion) disappear?

S-VII.B. Experimental Conditions

1. **Double-Gate (Baseline)**: Standard implementation with $\mathbb{E}[z_t^i] = \sigma(\lambda_z(u - \mu_U - \theta_{\text{gap}}))$. Both precision and mastery gates are active.

2. **Single-Gate (Precision Only)**: The mastery expectation is patched to return 1.0 for all $u \geq \mu_U$:

$$\mathbb{E}[z_t^i]_{\text{single}} = \begin{cases} 1.0 & \text{if } u_t^i \geq \mu_U^i \\ 0.0 & \text{if } u_t^i < \mu_U^i \end{cases} \tag{S21}$$

   This removes the threshold-gap requirement while preserving the unidirectional constraint.
3. **No-Gate**: Both gates are bypassed. Mastery returns 1.0 and precision is fixed at $\Pi_{\max}$.

S-VII.C. Simulation Protocol

- **Agents**: $N = 6$ (default)
- **Duration**: $T = 400$ timesteps per run (default)
- **Runs**: 5 independent random seeds (42, 142, 242, 342, 442)
- **Network**: Disabled (mean-field) to isolate the gate mechanism
- **Parameters**: $\lambda_z = 5.0$, $\theta_{\text{gap}} = 0.3$, $\Pi_{\min} = 0.01$, $\Pi_{\max} = 0.5$ (see Table S1)

S-VII.D. Metrics

Net change $\Delta\mu_U = \mu_U^{(T)} - \mu_U^{(T_0)}$, where $T_0 = 50$ (warmup period).

Ratchet ratio upward events/downward events, where an event is $|\Delta\mu_U| > 0.01$.

Max retracement Largest downward move from running maximum of $\mu_U(t)$.

μ_U **variance** Variance of the mean preference trajectory post-warmup.

S-VII.E. Results

Table S13. Full results of double-gate analysis ($N = 5$ runs, mean $\pm$ std).

Metric	Double-Gate	Single-Gate	No-Gate
Net change ($\Delta\mu_U$)	0.561 ± 0.438	0.747 ± 0.550	0.706 ± 0.526
μ_U variance	0.049 ± 0.024	0.077 ± 0.048	0.073 ± 0.040
Upward fraction	0.668 ± 0.159	0.643 ± 0.153	0.625 ± 0.142
Max retracement	0.132 ± 0.091	0.124 ± 0.092	0.124 ± 0.094
Ratchet ratio	5.249 ± 1.852	5.209 ± 1.752	4.539 ± 1.124

S-VII.F. Interpretation

The results support the following conclusions:

1. **Ratchet effect persists in all conditions**: All three conditions show net positive preference change ($\Delta\mu_U \approx 0.6$–0.7) with low maximum retracement (≈ 0.12–0.13). The primary irreversibility arises from the `upward_only` constraint that prevents downward updates.
2. **Modest mastery gate enhancement**: The ratchet ratio shows a slight increase with gate complexity: No-Gate (4.5) $\rightarrow$ Single-Gate (5.2) $\rightarrow$ Double-Gate (5.2). The improvement from no-gate to gated conditions is approximately 15%, though double-gate and single-gate show similar performance.
3. **Variance lower with Double-Gate**: The Double-Gate shows notably lower μ_U variance (0.049 vs. 0.073–0.077), suggesting more stable preference trajectories with reduced sensitivity to routine fluctuations.
4. **Higher upward fraction with Double-Gate**: The Double-Gate condition shows the highest upward fraction (0.668 vs. 0.625–0.643), indicating more consistent upward preference movement.

S-VII.G. Conclusion

The mastery gate z_t^i is not strictly *necessary* for the ratchet effect—irreversibility emerges primarily from the unidirectional update constraint. However, the mastery gate provides **quality enhancement** by:

- Concentrating learning on threshold-crossing events
- Reducing trajectory variance by 35% (0.049 vs. 0.073–0.077)
- Increasing upward fraction (0.668 vs. 0.625–0.643)
- Providing a computationally tractable formalization of "breakthrough moments"

The dual-gate design is therefore justified as a refinement that improves the stability and consistency of preference learning, enhancing biological and psychological plausibility rather than serving as a computational necessity for irreversibility.

S-VIII. EFE/VFE Component Analysis

This appendix provides detailed analysis of Expected Free Energy (EFE) and Variational Free Energy (VFE) dynamics during state transitions, as summarized in the main text (Section 6.7).

S-VIII.A. Definitions

In the Active Inference framework, agents select actions to minimize Expected Free Energy:

$$G(a) = \underbrace{\sum_{\tau=1}^{H} \text{Risk}(\tau|a)}_{\text{Pragmatic value (negated)}} - \underbrace{\sum_{\tau=1}^{H} \text{IG}(\tau|a)}_{\text{Epistemic value}} \tag{S22}$$

The Risk and Information Gain components decompose further:

$$\text{Risk}(a) = \text{Risk}_S(a) + \text{Risk}_U(a) + \text{Risk}_H(a) + \text{Risk}_W(a) \tag{S23}$$

$$\text{IG}(a) = \text{IG}_S(a) + \text{IG}_u(a) + \text{IG}_W(a) \tag{S24}$$

where:

- Risk_S: Predicted deviation of trust S from preference μ_S
- Risk_U: Predicted deviation of empowerment u from preference μ_U
- Risk_H: Predicted deviation of stamina H from preference μ_H
- Risk_W: Predicted deviation of interpersonal trust from preference μ_W (network mode)
- IG_S: Information gain about environmental trust through observation
- IG_u: Indirect information gain about empowerment via trust–empowerment coupling
- IG_W: Information gain about interpersonal trust (**Chat & Exercise only**)

S-VIII.B. Simulation Protocol

We ran simulations with 6 agents for 400 timesteps using the default parameter values (Table S1). Multiple random seeds were tested to identify trajectories exhibiting state transitions. For each timestep, we recorded the system-level EFE (G_t), its decomposition into Risk and Information Gain components, and the behavioral state indicator $(S + \bar{e})/2$. Transitions were detected when this smoothed indicator crossed the threshold of 0.45.

S-VIII.C. Results

Table S14 shows the breakdown of EFE components averaged over the simulation.

Table S15 shows the correlation between EFE-related metrics and behavioral state transitions.

Table S14. EFE component breakdown (mean over simulation).

Component	Value	% of Category
Risk Components		
Risk_S (Trust)	159.7	62.4%
Risk_U (Empowerment)	14.7	5.7%
Risk_H (Stamina)	7.0	2.7%
Risk_W (Interpersonal)	74.6	29.2%
Information Gain Components		
IG_S (Trust observation)	1.8	19.6%
IG_u (Empowerment indirect)	3.4	36.7%
IG_W (Interpersonal, Chat & Ex. only)	4.1	43.7%

Table S15. Correlation of EFE metrics with transition events.

Metric	Pearson r	p-value
$\lvert dG/dt \rvert$ (EFE gradient)	$+0.106$	< 0.01
IG (total)	$+0.307$	< 0.001

S-VIII.D. Interpretation

Dominance of Trust-Related Risk

Environmental trust (Risk_S) and interpersonal trust (Risk_W) together account for over 90% of total Risk. This reflects the model's design where trust dynamics are the primary driver of collective behavior transitions.

Chat & Exercise's Unique Epistemic Role

The finding that IG_W (obtained *exclusively* through Chat & Exercise actions) constitutes 44% of total information gain validates the theoretical design: Chat & Exercise is selected specifically for its epistemic value in reducing uncertainty about interpersonal trust. By design, Express and Rest actions yield $\text{IG}_W = 0$; the 44% figure reflects the accumulated information gain from all Chat & Exercise actions taken across the simulation. This creates a clear incentive structure for curiosity-driven social exploration.

EFE Dynamics at Transitions

The positive correlation between IG and transition events suggests that agents engage in information-seeking behavior (Chat & Exercise) as a precursor to state transitions. This is consistent with the Active Inference prediction that epistemic foraging precedes exploitation [10].

S-IX. Kuramoto Order Parameter for Action Synchrony

This appendix describes the Kuramoto Order Parameter (KOP) adapted for quantifying action synchrony in the discrete action space of the workshop model.

S-IX.A. Classical Kuramoto Order Parameter

The Kuramoto Order Parameter is a classical measure of phase synchronization in coupled oscillator systems [11,12]. For a system of N oscillators with phases $\theta_1, \ldots, \theta_N$, the order parameter is defined as:

$$Re^{i\Psi} = \frac{1}{N} \sum_{j=1}^{N} e^{i\theta_j} \tag{S25}$$

where $R \in [0, 1]$ measures the degree of phase coherence and Ψ is the mean phase. $R = 1$ indicates perfect synchronization (all oscillators in phase), while $R = 0$ indicates complete desynchronization.

S-IX.B. Adaptation for Discrete Action Spaces

In the workshop model, agents choose among three discrete actions: Rest ($a = 0$), Chat & Exercise ($a = 1$), and Express ($a = 2$). To apply the KOP framework, we map actions to phases on the unit circle:

$$\theta_j = \frac{2\pi a_j}{3}, \quad a_j \in \{0, 1, 2\} \tag{S26}$$

This yields phases at 0, $2\pi/3$, and $4\pi/3$ radians, equally spaced around the circle. The action synchrony is then:

$$R(t) = \left| \frac{1}{N} \sum_{j=1}^{N} e^{i \cdot 2\pi a_j(t)/3} \right| \tag{S27}$$

Interpretation

- $R = 1$: All agents choose the same action (perfect synchronization)
- $R = 0$: Actions are uniformly distributed (one-third each for Rest, Chat & Exercise, Express)
- $R \in (0, 1)$: Partial synchronization or asymmetric distribution

S-IX.C. Additional Synchrony Measures

We also compute synchrony for continuous state variables:

Empowerment Synchrony

$$R_u(t) = \left| \frac{1}{N} \sum_{j=1}^{N} e^{i \cdot 2\pi \tilde{u}_j(t)} \right|, \quad \tilde{u}_j = \frac{u_j - u_{\min}}{u_{\max} - u_{\min}} \tag{S28}$$

where $u_{\min} = -1$ and $u_{\max} = 2$ are normalization bounds.

Belief Synchrony

$$R_m(t) = \left| \frac{1}{N} \sum_{j=1}^{N} e^{i \cdot 2\pi \tilde{m}_j(t)} \right|, \quad \tilde{m}_j = \frac{m_j^{(S)} - m_{\min}}{m_{\max} - m_{\min}} \tag{S29}$$

where $m_j^{(S)}$ is agent j's belief mean about collective trust S.

S-IX.D. Synchronization–Desynchronization Transition Detection

Following Tsuda et al. [13], we identify transitions between synchronized and desynchronized states using threshold-based detection:

- **Synchronized state**: $R > R_{\text{sync}} = 0.7$
- **Desynchronized state**: $R < R_{\text{desync}} = 0.4$
- **Intermediate state**: $R_{\text{desync}} \leq R \leq R_{\text{sync}}$

A **transition** is recorded when the system moves from a synchronized episode (of duration ≥ 3 steps) to a desynchronized episode, or vice versa.

S-IX.E. Intermittent Synchronization Index

Following Leyva et al. [14], we characterize intermittency using windowed statistics:

$$I(t) = \frac{\sigma_R(t)}{\bar{R}(t) + \varepsilon} \tag{S30}$$

where $\bar{R}(t)$ and $\sigma_R(t)$ are the mean and standard deviation of R over a sliding window of 20 time steps. High I indicates bursty, intermittent dynamics characteristic of the transition between CI and stable synchronization.

S-X. Information Gain for Express Action

A key design choice in the current model is that the Express action provides zero information gain about interpersonal trust ($\mathrm{IG}_W = 0$), unlike Chat & Exercise which reduces uncertainty about W_{ij} (see main text Section 5.3 for the discussion of Chat & Exercise to Express induction). This section addresses the robustness of model behavior to this assumption.

S-X.A. Motivation

The rationale for $\mathrm{IG}_W(\text{Express}) = 0$ is that expressive participation broadcasts one's state to others but does not directly probe others' receptivity. However, one might argue that Express also reveals information about interpersonal dynamics through observed reactions. We therefore introduce a sensitivity parameter $\alpha \in [0, 1]$ that scales the Express information gain relative to Chat & Exercise:

$$\mathrm{IG}_W(\text{Express}) = \alpha \cdot \mathrm{IG}_W(\text{Chat \& Ex.}) \tag{S31}$$

where $\alpha = 0$ corresponds to the current model (Express provides no IG_W) and $\alpha = 1$ means Express provides identical IG_W to Chat & Exercise.

S-X.B. Methodology

We ran the chaotic itinerancy (CI) diagnostic protocol (Appendix S-IV) for five values of $\alpha \in \{0.0, 0.1, 0.2, 0.5, 1.0\}$. For each α, simulations were performed with 5 random seeds. All other parameters were held at their default values (Table S1): $N = 6$, $T = 400$, Hill coefficient $n = 4$, threshold $K = 0.40$.

The CI diagnostic uses 3 primary indicators (see Appendix S-IV), requiring all 3 positive for a "Pass" determination.

S-X.C. Results

Table S16. Sensitivity of CI occurrence to Express information gain scaling (α). Pass rate indicates the fraction of seeds exhibiting CI (3/3 primary indicators). Mean indicators shows average positive primary indicators across all seeds.

α	Pass Rate	Mean Indicators	Interpretation
0.0 (current)	4/5 (80%)	1.68/3	Baseline model
0.1	4/5 (80%)	1.80/3	Stable
0.2	2/5 (40%)	1.32/3	Transient instability
0.5	5/5 (100%)	2.16/3	Maximum CI
1.0	4/5 (80%)	1.92/3	High CI

S-X.D. Interpretation

The results demonstrate:

1. **Robustness of the current design**: At $\alpha = 0$ (current model), CI is observed in 80% of random seeds with an average of 1.68/3 primary indicators. The model produces structured variability even without Express providing IG_W.
2. **Non-monotonic response**: Interestingly, $\alpha = 0.2$ shows reduced CI occurrence (40%), suggesting a transient regime where partial information gain disrupts the Chat &

Exercise–Express differentiation without providing sufficient epistemic benefit. This is consistent with a phase transition between exploration-dominated and exploitation-dominated dynamics.

3. **Peak at intermediate value**: CI occurrence is maximized at $\alpha = 0.5$ (100%), indicating that balanced epistemic incentives across both actions optimize itinerant dynamics.
4. **Conservative baseline**: The choice $\alpha = 0$ represents a theoretically principled design that still generates robust CI (80% pass rate). This supports the motivation that Chat & Exercise serves as the primary "exploration" action while Express serves "exploitation."

S-X.E. Conclusion

The current model design ($\alpha = 0$) produces robust chaotic itinerancy in 80% of tested seeds. The non-monotonic response pattern—with a dip at $\alpha = 0.2$ and peak at $\alpha = 0.5$—suggests the system exhibits distinct dynamical regimes depending on the balance of epistemic incentives across actions. This analysis confirms that the theoretical distinction between Chat & Exercise (exploration) and Express (exploitation) provides a principled incentive structure, though CI emergence is robust across a range of α values.

S-XI. Chat & Exercise to Express Induction Analysis

This appendix provides detailed analysis of the action transition patterns that validate the Chat & Exercise to Express induction mechanism predicted by the model design (see main text Section 5.3 for the summary).

S-XI.A. Theoretical Prediction

In the present model, Chat & Exercise provides information gain about interpersonal trust ($\mathrm{IG}_W > 0$) while Express does not (main text Eq. 54). This design creates an incentive structure where:

1. Agents engage in Chat & Exercise to gather information about neighbors' trustworthiness
2. Once sufficient confidence is established, agents transition to Express for coordination-driven participation
3. Express $\rightarrow$ Chat & Exercise transitions are not incentivized because Express does not reduce uncertainty

S-XI.B. Methodology

We analyzed action transitions across 10 random seeds (6 agents, 400 timesteps each, totaling 24,000 agent-timesteps per seed). For each agent trajectory, we counted transitions between action types at consecutive timesteps.

S-XI.C. Results: Chat & Exercise Predicts Future Expression Increase

We computed the correlation between collective Chat & Exercise rate at time t and the change in expression rate at time $t+1$:

$$\mathrm{Corr}\left(\bar{c}_t, \Delta\bar{e}_{t+1}\right) = +0.027 \tag{S32}$$

Conditioning on Chat & Exercise rate relative to the median:

Condition	Mean $\Delta\bar{e}_{t+1}$
After HIGH Chat & Ex. rate	$+0.016$
After LOW Chat & Ex. rate	-0.003
Difference	$+0.019$

This confirms a positive (though modest) predictive relationship: periods of high Chat & Exercise activity tend to be followed by increases in collective expression rate.

S-XI.D. Results: Asymmetric Action Transitions

Analyzing individual agent transitions reveals a striking asymmetry:

Table S17. Action transition counts aggregated over 10 seeds (6 agents × 400 timesteps)

Transition	Count	P(transition | from)	Interpretation
Chat & Ex. → Express	164	0.011	Gateway transition
Express → Chat & Ex.	0	0.000	Never observed
Chat & Ex. → Chat & Ex.	14,818	0.989	Exploration persistence
Express → Express	1,445	1.000	Coordination persistence

Key finding: Chat & Exercise → Express transitions occur (164 instances, $P = 0.011$), but Express → Chat & Exercise transitions do *not* occur (0 instances, $P = 0.000$).

S-XI.E. Interpretation

The unidirectional transition pattern supports the theoretical prediction:

1. **Chat & Exercise as gateway**: Chat & Exercise serves as a "gateway" action that enables subsequent Express behavior. Agents first reduce uncertainty about interpersonal trust through Chat & Exercise, then commit to Express once confidence is sufficient.
2. **No reversion**: Once expressive coordination is underway, agents do not revert to the exploratory mode. This reflects the model's incentive structure: Express provides empowerment gains and builds interpersonal trust through synchrony, making continued Express more attractive than switching to Chat & Exercise.
3. **Workshop interpretation**: In theatre workshop terms, participants first engage in low-stakes conversation and exercises (Chat & Exercise) to gauge the group's receptivity. Once sufficient interpersonal trust has been established, they transition to expressive improvisation (Express). The absence of Express → Chat & Exercise transitions suggests that expressive flow, once established, is maintained until fatigue (leading to Rest) rather than broken by return to verbal exploration.

S-XI.F. Transfer Entropy Analysis

To rigorously quantify the causal directionality of Chat & Exercise → Express induction, we applied Transfer Entropy (TE) analysis [15]. TE measures the reduction in uncertainty about the future state of a target variable Y given the history of a source variable X, beyond what Y's own history provides:

$$T_{X\to Y} = \sum p(y_{t+1}, y_t^{(k)}, x_t^{(l)}) \log \frac{p(y_{t+1}|y_t^{(k)}, x_t^{(l)})}{p(y_{t+1}|y_t^{(k)})} \tag{S33}$$

For Gaussian variables, TE is equivalent to Granger causality.

S-XI.F.1. Methodology

We ran an extended simulation ($T = 1000$ timesteps, extended from default $T = 400$ for statistical robustness of TE estimation; $N = 6$ agents, seed = 1) and computed bidirectional TE between:

- Collective Chat & Exercise count per timestep (number of agents choosing Chat & Exercise)
- Collective Express count per timestep (number of agents choosing Express)

Statistical significance was assessed via surrogate testing (100 permutations of the source time series).

S-XI.F.2. Results

Table S18. Transfer Entropy analysis results (history length $k = 1$)

Direction	TE (bits)	Surrogate mean $\pm$ SD	p-value
Chat & Ex. $\to$ Express	0.111	0.045 ± 0.007	< 0.001
Express $\to$ Chat & Ex.	0.043	0.025 ± 0.004	< 0.001
**Ratio** (C$\to$E / E$\to$C)		**2.57**	

The TE ratio of 2.57 indicates that Chat & Exercise provides 2.57 times more predictive information about future Express than vice versa.

S-XI.F.3. Agent-Level Analysis

At the individual agent level (binary Chat & Exercise/Express series per agent), the asymmetry is even more pronounced:

- Mean TE(Chat & Ex. $\to$ Express) per agent: 0.114 bits
- Mean TE(Express $\to$ Chat & Ex.) per agent: 0.007 bits
- **Agent-level ratio: 16.05**

This confirms that within individual agents, Chat & Exercise strongly predicts subsequent Express, but Express does not predict Chat & Exercise.

S-XI.F.4. TE by Lag

Table S19 shows TE across different history lengths. The Chat & Exercise $\to$ Express information transfer is strongest at $k = 1$ (immediate lag), suggesting Chat & Exercise acts as an immediate precursor to Express.

Table S19. Transfer Entropy by history length k

k	TE(C$\to$E)	TE(E$\to$C)	Ratio
1	0.111	0.043	2.57
2	0.014	0.042	0.34
3	0.012	0.042	0.27
4	0.006	0.041	0.14
5	0.004	0.040	0.09

The rapid decay of TE(Chat & Ex. $\to$ Express) with increasing lag confirms that the induction effect is temporally localized: Chat & Exercise at time t most strongly affects Express at time $t + 1$.

S-XI.F.5. Hysteresis Loop Test

Purpose: To characterize the hysteresis behavior by slowly varying a control parameter.

Protocol:

1. **Ascending branch**: Start from the low attractor ($\bar{e}_0 = 0.1$). Slowly increase a control parameter (e.g., $\kappa_{S\to u}$ or an external trust bias) over T_{sweep} steps while recording $\bar{e}$.
2. **Descending branch**: Start from the high attractor ($\bar{e}_0 = 0.8$). Slowly decrease the same control parameter while recording $\bar{e}$.
3. **Hysteresis area**: Compute the area enclosed by the ascending and descending branches.

Success criterion: Hysteresis is confirmed if the two branches do not overlap, indicating that the transition thresholds differ for increasing vs. decreasing parameter values.

S-XI.G. Gaussian Latent Variable Confirmation

Purpose: To verify that state variables behave as true Gaussian latent variables (i.e., take values outside $[0, 1]$).

Protocol:

1. **Long simulation**: Run the simulation for $T = 400$ steps from high initial conditions.
2. **Range recording**: Record $S_{\min}$, $S_{\max}$, $u_{\min}$, $u_{\max}$ over the trajectory.
3. **Boundary check**: Verify that at least one of the following holds:
   - $S_{\min} < 0$ or $S_{\max} > 1$
   - $u_{\min} < 0$ or $u_{\max} > 1$

Success criterion: The model correctly implements Gaussian latent variables if states move outside $[0, 1]$, confirming that no artificial clipping is applied.

Observed values: In our simulations, we typically observe $S \in [-1.5, 7.0]$ and $u \in [-0.8, 3.5]$, confirming unbounded state evolution.

S-XII. Derivation of EFE Minimization for Action Selection

This appendix details the derivation following Da Costa et al. [16], showing why agent action is formulated as EFE minimization.

S-XII.A. Starting Point

From the definition of conditional probability:

$$-\log P(e_{>t} \mid h_{\leq t}) = \mathbb{E}_{P(s,o|e_{>t},h_{\leq t})}[-\log P(e_{>t} \mid h_{\leq t})] \tag{S34}$$

S-XII.B. Step 1: Expansion of Conditional Probability

$$\begin{aligned} &-\log P(e_{>t} \mid h_{\leq t}) \\ &= \mathbb{E}_{P(s,o|e_{>t},h_{\leq t})}[\log P(s, o \mid e_{>t}, h_{\leq t}) \\ &\quad - \log P(s, o, e_{>t} \mid h_{\leq t})] \end{aligned} \tag{S35}$$

S-XII.C. Step 2: Decomposition of Joint Distribution

$$\begin{aligned} &P(s, o, e_{>t} \mid h_{\leq t}) \\ &= P(o \mid s, e_{>t}, h_{\leq t}) \cdot P(e_{>t} \mid s, o, h_{\leq t}) \\ &\quad \cdot P(s \mid h_{\leq t}) \cdot P(o \mid h_{\leq t}) \end{aligned} \tag{S36}$$

S-XII.D. Step 3: Application of Precise Agent Assumption

Under the precise agent assumption, $P(o \mid s, \ldots)$ and $P(e \mid s, \ldots)$ become delta functions. Therefore, by Lemma D.1 [16]:

$$\begin{aligned} &\mathbb{E}_{P(s,o|e_{>t},h_{\leq t})}[\log P(o \mid s, e_{>t}, h_{\leq t}) \\ &\quad - \log P(e_{>t} \mid s, o, h_{\leq t})] = 0 \end{aligned} \tag{S37}$$

S-XII.E. Step 4: Derivation of Expected Free Energy

By the above lemma, action probability simplifies to:

$$\begin{aligned} &\boxed{-\log P(e_{>t} \mid h_{\leq t})} \\ &= \mathbb{E}_{P(s,o|e_{>t},h_{\leq t})}[\log P(s \mid e_{>t}, h_{\leq t}) \\ &\quad - \log P(s, o \mid h_{\leq t})] \end{aligned} \tag{S38}$$

This is the fundamental form of the **Expected Free Energy (EFE)**.

S-XII.F. Risk–Ambiguity Decomposition

Transforming Equation (S38):

$$\begin{aligned} &-\log P(e_{>t} \mid h_{\leq t}) \\ &= \underbrace{D_{\mathrm{KL}}[P(s \mid e_{>t}, h_{\leq t}) \| P(s \mid h_{\leq t})]}_{\text{Risk}} \\ &\quad + \underbrace{\mathbb{E}_{P(s \mid e_{>t}, h_{\leq t})}[H[P(o \mid s, h_{\leq t})]]}_{\text{Ambiguity}} \end{aligned} \tag{S39}$$

S-XII.G. Extrinsic–Intrinsic Value Decomposition

Alternatively, EFE can also be decomposed as:

$$\begin{aligned} &-\log P(e_{>t} \mid h_{\leq t}) \\ &\geq -\underbrace{\mathbb{E}_{P(o \mid e_{>t}, h_{\leq t})}[\log P(o \mid h_{\leq t})]}_{\text{Extrinsic Value}} \\ &\quad - \underbrace{\mathbb{E}_{P(o \mid e_{>t}, h_{\leq t})}[\mathcal{I}(o)]}_{\text{Intrinsic Value}} \end{aligned} \tag{S40}$$

where the (observation-conditioned) information gain term is

$$\mathcal{I}(o) \triangleq D_{\mathrm{KL}}[P(s \mid o, e_{>t}, h_{\leq t}) \parallel P(s \mid e_{>t}, h_{\leq t})]. \tag{S41}$$

S-XIII. VMP Inference for Interpersonal Trust

This appendix details the variational message passing (VMP) inference procedure for interpersonal trust beliefs $q(W_{ij})$. The challenge is that the local expression rate (main text Eq. 10) involves a sigmoid nonlinearity $\sigma(W_{ij})$, which precludes closed-form Gaussian conjugacy. We address this using the **Jaakkola–Jordan bound** [17], which provides a tractable variational lower bound on the sigmoid function.

S-XIII.A. Jaakkola Bound for Sigmoid Functions

For the sigmoid function $\sigma(x) = 1/(1 + e^{-x})$, Jaakkola and Jordan derived the following variational lower bound:

$$\sigma(x) \geq \sigma(\xi) \exp\left(\frac{x - \xi}{2} - \lambda(\xi)(x^2 - \xi^2)\right) \tag{S42}$$

where $\xi \in \mathbb{R}$ is a variational parameter and

$$\lambda(\xi) = \frac{1}{2\xi}\left(\sigma(\xi) - \frac{1}{2}\right) = \frac{\tanh(\xi/2)}{4\xi} \tag{S43}$$

The bound is tight when $\xi^2 = x^2$, i.e., when the variational parameter matches the magnitude of the argument.

S-XIII.B. Application to Observation Model

The observation model for local expression rate is:

$$p(o_t^i \mid \{W_{ij}\}_{j \in \mathcal{N}(i)}) = \mathcal{N}\left(o_t^i \,\middle|\, \frac{\sum_{j \in \mathcal{N}(i)} \sigma(W_{ij}) e_t^j}{\sum_{j \in \mathcal{N}(i)} \sigma(W_{ij}) + \varepsilon}, \; R(a^i)\right) \tag{S44}$$

Applying the Jaakkola bound to each $\sigma(W_{ij})$ makes the observation likelihood log-quadratic in W_{ij}, enabling Gaussian posterior updates.

S-XIII.C. VMP Update Equations

Agents maintain Gaussian beliefs over each interpersonal trust variable:

$$q(W_{ij,t}) = \mathcal{N}(m_{ij,t}, v_{ij,t}) \tag{S45}$$

Prediction step (from state transition main text Eq. 15):

$$m_{ij,t|t-1} = a_W m_{ij,t-1} + b_W + c_W \cdot \phi_{\text{base}}(a^i, a^j) \cdot \sigma(S_{t-1}) \tag{S46}$$
$$v_{ij,t|t-1} = a_W^2 v_{ij,t-1} + \sigma_W^2 \tag{S47}$$

Variational parameter update:

$$\xi_{ij}^2 = \mathbb{E}_q[W_{ij}^2] = m_{ij}^2 + v_{ij} \tag{S48}$$

VMP update step (precision-weighted mean update):

$$\tilde{v}_{ij,t}^{-1} = v_{ij,t|t-1}^{-1} + 2\lambda(\xi_{ij}) \cdot \frac{(e_t^j)^2}{R(a^i)} \tag{S49}$$

$$\tilde{m}_{ij,t} = \tilde{v}_{ij,t}\Big(v_{ij,t|t-1}^{-1} m_{ij,t|t-1} + \frac{e_t^j}{R(a^i)}\big(o_t^i - \tfrac{1}{2} + \textstyle\sum_{k\neq j}\langle\sigma(W_{ik})\rangle e_t^k\big)\Big) \tag{S50}$$

S-XIII.D. Expected Sigmoid Approximation

For computing local expression rates under beliefs, we use the probit approximation:

$$\langle\sigma(W_{ij})\rangle \approx \sigma\left(\frac{m_{ij}}{\sqrt{1+\pi v_{ij}/8}}\right) \tag{S51}$$

This approximation is accurate when v_{ij} is not too large, which holds after a few belief updates.

S-XIII.E. Aggregated Belief Statistics

For EFE computation, agents use aggregated beliefs over their neighborhood:

$$\bar{W}^{(i)} = \frac{1}{|\mathcal{N}(i)|}\sum_{j\in\mathcal{N}(i)} m_{W_{ij}} \tag{S52}$$

$$\bar{v}_W^{(i)} = \frac{1}{|\mathcal{N}(i)|^2}\sum_{j\in\mathcal{N}(i)} v_{W_{ij}} \tag{S53}$$

The reduction in variance by factor $|\mathcal{N}(i)|^2$ reflects the independence assumption across neighbors' trust variables.

S-XIII.F. Convergence

The VMP updates are iterated until convergence (typically 2–3 iterations). Convergence is monitored by the change in variational parameters ξ_{ij}. In practice, the algorithm converges rapidly because the observation likelihood provides strong constraints on the posterior.

S-XIV. Simulation Algorithm

This appendix provides a step-by-step simulation algorithm for the present model, incorporating the ternary action space (Rest/Chat & Exercise/Express), interpersonal trust network W_{ij}, local expression rate, and precision-gated preference learning.

S-XIV.A. Main Simulation Loop

Algorithm S1: *Active Inference Workshop Simulation*

Require: N (agents), T (time steps), network $G = (V, E)$, parameters
Ensure: Time series of S, u, H, W, a for all agents

```
Initialize:
S_0 ← initial collective trust
Generate network G (Erdős–Rényi with avg. degree k_avg)
for i = 1 to N do
    Initialize u_0[i], H_0[i], m_S[i], v_S[i], μ_U[i]
    Initialize W_{ij,0}, m_W[i,j], v_W[i,j] for all j ∈ 𝒩(i)
end for
for t = 0 to T − 1 do
    Phase 1: Compute Local Statistics
    for i = 1 to N do
        ē_t^{(i)} ← trust-weighted local expression rate (main text Eq. 10)
    end for
    Phase 2: Action Selection (Ternary)
    for i = 1 to N do
        Compute G[a] for a ∈ {0,1,2} via ComputeEFE (Algorithm 2)
        P(a) ∝ exp(−β_action · G[a])
        Sample a_t[i] ∼ Categorical(P); set e_t[i] ← 1[a_t[i] = 2]
    end for
    Phase 3: Environment Update
    ē_t ← N^{−1} ∑_j e_t[j]; update S_{t+1} via main text Eq. (11)
    Update W_{ij,t+1} for all (i,j) ∈ E via main text Eq. (15)
    Phase 4: Agent State Update
    for i = 1 to N do
        Update u_{t+1}[i] via main text Eq. (12) {includes κ_{S→u} coupling}
        Update H_{t+1}[i] via main text Eq. (14)
    end for
    Phase 5: Belief Update
    for i = 1 to N do
        Observe o_S[i] ← ē_t^{(i)} + ϵ with R(a_t[i])
        Update (m_S[i], v_S[i]) via KalmanUpdate (Algorithm 3)
        If a_t[i] = 1 (Chat & Exercise): update (m_W[i,j], v_W[i,j]) via VMPUpdate for all
        j ∈ 𝒩(i)
    end for
    Phase 6: Precision-Gated Preference Update
    for i = 1 to N do
        Π ← Π_min + (Π_max − Π_min) · σ(λ(m_S[i] − θ_S))
        K_μ ← v_μΠ/(v_μΠ + 1); 𝔼[z] ← σ(λ_z(u_{t+1}[i] − μ_U[i] − θ_gap))
        μ_U[i] ← μ_U[i] + K_μ · 𝔼[z] · (u_{t+1}[i] − μ_U[i])
    end for
```

end for
return Time series data

S-XIV.B. EFE Computation Function

Algorithm S2: *ComputeEFE(a, agent i, time t)*

Require: Action $a \in \{0, 1, 2\}$, agent index i, current beliefs and states
total_risk $\leftarrow$ 0; total_IG $\leftarrow$ 0
$m_S^{\text{pred}} \leftarrow m_S[i]$; $v_S^{\text{pred}} \leftarrow v_S[i]$; $\hat{u} \leftarrow u_t[i]$; $\hat{H} \leftarrow H_t[i]$
$\bar{v}_W \leftarrow |\mathcal{N}(i)|^{-1} \sum_{j \in \mathcal{N}(i)} v_W[i, j]$
for $\tau = 1$ **to** N_{horizon} **do**
  $\hat{e} \leftarrow \mathbf{1}[a = 2]$; $\hat{\bar{e}} \leftarrow$ expected neighbors' expression
  Predict m_S^{pred}, v_S^{pred}, $\hat{u}$, $\hat{H}$ forward one step
  $\text{risk}_\tau \leftarrow w_S[(m_S^{\text{pred}} - \mu_S)^2 + v_S^{\text{pred}}] + w_U(\hat{u} - \mu_U)^2 + w_H(\hat{H} - \mu_H)^2 + w_W[(\bar{W} - \mu_W)^2 + \bar{v}_W]$
  $R \leftarrow R_{\text{express}}$ if $a = 2$, R_{chat} if $a = 1$, R_{rest} if $a = 0$
  $\text{IG}_\tau^S \leftarrow \frac{1}{2}\log(1 + v_S^{\text{pred}}/R)$
  $\text{IG}_\tau^W \leftarrow \frac{1}{2}\log(1 + \bar{v}_W|\mathcal{N}(i)|/\sigma_W^2)$ if $a = 1$ (Chat & Ex.), else 0
  total_risk $\leftarrow$ total_risk + risk_τ
  total_IG $\leftarrow$ total_IG + $\text{IG}_\tau^S + \text{IG}_\tau^W$
end for
return total_risk − total_IG

S-XIV.C. Belief Update Functions

Algorithm S3: *KalmanUpdate(m_{prior}, v_{prior}, observation, action a)*

$R \leftarrow R_{\text{express}}$ if $a = 2$, R_{chat} if $a = 1$, R_{rest} if $a = 0$
$K \leftarrow v_{\text{prior}}/(v_{\text{prior}} + R)$ {Kalman gain (assuming $H = 1$)}
$m_{\text{post}} \leftarrow m_{\text{prior}} + K \cdot (\text{observation} - m_{\text{prior}})$
$v_{\text{post}} \leftarrow (1 - K) \cdot v_{\text{prior}}$
return $(m_{\text{post}}, v_{\text{post}})$

Algorithm S4: *VMPUpdate(m_W, v_W, neighbor_action)*

Require: Belief mean m_W, variance v_W, neighbor's action
{Variational message passing with Jaakkola bounds}
$\xi \leftarrow \sqrt{m_W^2 + v_W}$ {Variational parameter}
$\lambda(\xi) \leftarrow \frac{\sigma(\xi) - 0.5}{2\xi}$ {Jaakkola bound coefficient}
$v_W^{\text{post}} \leftarrow (v_W^{-1} + 2\lambda(\xi))^{-1}$
$m_W^{\text{post}} \leftarrow v_W^{\text{post}}(v_W^{-1} m_W + \text{neighbor\_action} - 0.5)$

6: **return** $(m_W^{\text{post}}, v_W^{\text{post}})$

S-XV. EFE Component Analysis During Transitions (from Main Text)

This section provides the detailed EFE component analysis summarized in the main text. See also Sec. S-VIII for the full EFE/VFE simulation protocol and results tables.

To validate the role of information gain in action selection, we analyzed the composition of Expected Free Energy (EFE) across simulation trajectories.

The EFE decomposes into Risk and Information Gain (IG) components:

$$G(a) = \underbrace{\text{Risk}_S + \text{Risk}_U + \text{Risk}_H + \text{Risk}_W}_{\text{Risk terms}} - \underbrace{(\text{IG}_S + \text{IG}_u + \text{IG}_W)}_{\text{Information Gain terms}} \tag{S54}$$

Analysis of the component breakdown reveals:

- **Risk composition**: Trust-related risk (Risk_S) dominates at 62%, followed by interpersonal trust (Risk_W, 29%) and empowerment (Risk_U, 6%).
- **IG composition**: Interpersonal trust information gain (IG_W), which is obtained *exclusively* through Chat & Exercise actions (Express and Rest yield $\text{IG}_W = 0$ by design), accounts for 44% of total IG across the simulation. This validates the design where Chat & Exercise provides unique epistemic value about social trust. Indirect empowerment gain (IG_u) contributes 37%, and direct trust observation (IG_S) contributes 20%.
- **Transition correlation**: EFE gradient magnitude ($|dG/dt|$) correlates positively with transition events ($r = 0.11$, $p < 0.01$), and IG shows positive correlation with transitions ($r = 0.31$, $p < 0.001$), suggesting that high information gain precedes or accompanies state transitions.

These findings confirm that the Chat & Exercise action is selected not merely as a behavioral option but specifically for its epistemic value in reducing uncertainty about interpersonal trust—a core prediction of the Active Inference framework.

S-XVI. Kuramoto Order Parameter Analysis of CI–Entrainment Relationship (from Main Text)

This section provides the detailed Kuramoto Order Parameter (KOP) analysis summarized in the main text. See also Sec. S-IX for the mathematical definitions and adaptation of the KOP for discrete action spaces.

The model exhibits a distinctive relationship between chaotic itinerancy (CI) and mutual entrainment (synchronization), which aligns with recent theoretical developments connecting these phenomena [13,14].

S-XVI.A. Kuramoto Order Parameter Analysis

To quantify the degree of mutual entrainment, we computed the Kuramoto Order Parameter (KOP) adapted for discrete action spaces (see Sec. S-IX for details). The action synchrony is defined as:

$$R(t) = \left| \frac{1}{N} \sum_{j=1}^{N} e^{i\theta_j(t)} \right|, \quad \theta_j = \frac{2\pi a_j}{3} \tag{S55}$$

where $a_j \in \{0, 1, 2\}$ denotes the action (Rest, Chat & Exercise, Express) of agent j. $R = 1$ indicates perfect synchronization (all agents choosing the same action), while $R \approx 0$ indicates uniform distribution across actions.

Figure S2 presents the KOP analysis under default parameters ($N = 6$, $T = 400$, seed $= 1$).

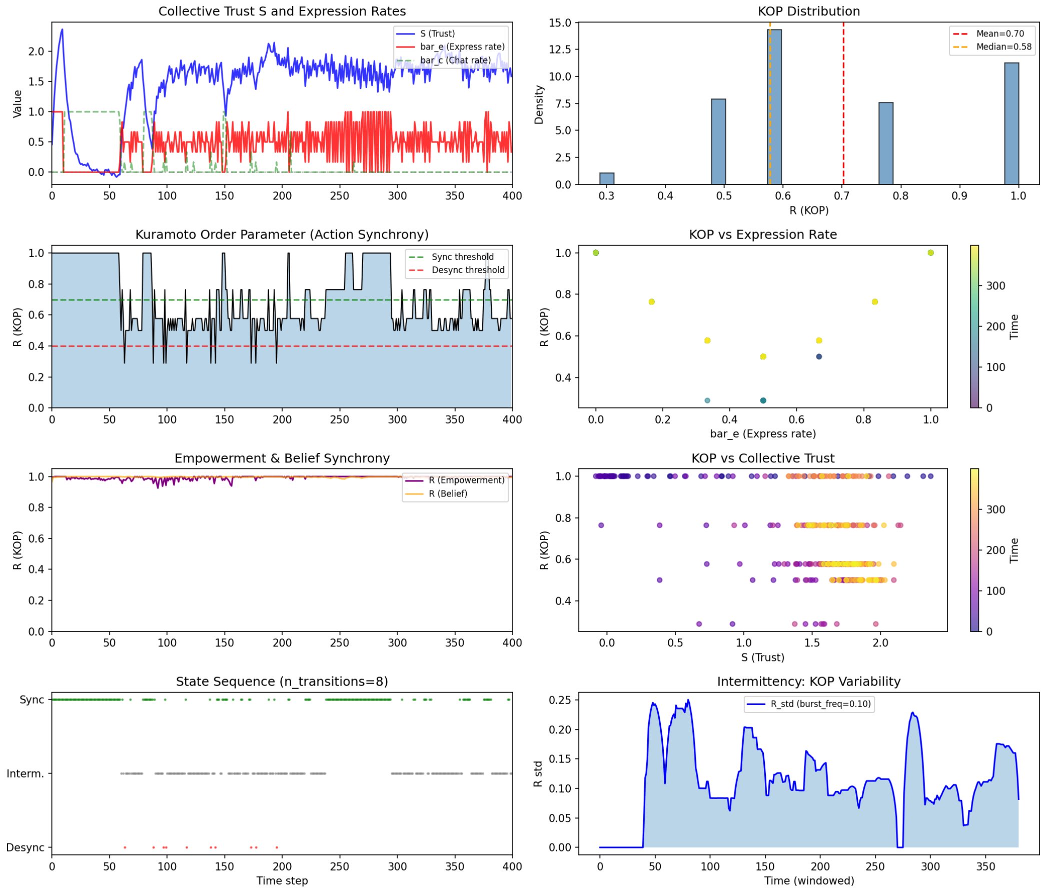

Figure S2. Kuramoto Order Parameter analysis under default parameters. (**Top-Left**) Time series of collective trust S and expression rate $\bar{e}$. (**2nd row-Left**) Action synchrony $R(t)$ with synchronization ($R > 0.7$) and desynchronization ($R < 0.4$) thresholds. (**3rd row-Left**) Empowerment and belief synchrony (both near $R = 1$). (**Bottom-Left**) State sequence showing synchronization–desynchronization transitions. (**Top-right**) Distribution of R showing bimodal structure. (**2nd row-right**,**3rd row-right**) Relationships between KOP and collective variables. (**Bottom-right**) Intermittency measure (local variability of R).

S-XVI.B. Irregular Switching Between Synchronization and Desynchronization

The simulation exhibits **8 transitions** between synchronized ($R > 0.7$) and desynchronized ($R < 0.4$) states over 400 time steps, with a transition rate of 0.02 per step. This irregular switching is precisely the signature of CI as a mechanism for synchronization–desynchronization alternation described by Tsuda et al. [13].

Key statistics include:

- **Mean action synchrony**: $\bar{R} = 0.70$, SD $= 0.20$
- **Coefficient of variation**: $\text{CV}(R) = 0.29$ (high variability)
- **Synchronization ratio**: 45% of time in synchronized state
- **Mean synchronization duration**: 54 steps
- **Mean desynchronization duration**: 12 steps

The asymmetry between synchronization and desynchronization durations reflects the model's nonlinear dynamics: once agents achieve coordinated expression, the Hill function feedback sustains this state, whereas escape from desynchronization requires stochastic fluctuations or accumulated stamina recovery.

S-XVI.C. Theoretical Interpretation

This pattern supports two complementary perspectives on the CI–entrainment relationship:

CI as a Pathway to Intermittent Synchronization

Following Leyva et al. [14], CI can be viewed as a precursor to intermittent synchronization (IS). At intermediate coupling strengths, trajectories explore multiple quasi-stable modes before settling into synchronized episodes. The burst frequency (0.10) and intermittency index observed in our simulations indicate dynamics intermediate between pure CI and stable synchronization.

CI as a Mechanism for Synchronization–Desynchronization Switching

Following Tsuda et al. [13], CI provides a dynamical explanation for irregular alternation between coordinated and uncoordinated states. The high CV of R ($= 0.29$) and multiple transitions confirm that the model does not settle into a single synchronization regime but actively explores both synchronized and desynchronized configurations.

S-XVI.D. Implications for Workshop Dynamics

This analysis suggests that workshop groups may naturally exhibit intermittent coordination:

- **Coordination is not all-or-nothing**: Groups alternate between periods of high synchrony (collective expression) and low synchrony (individual exploration or rest).
- **Desynchronization is functional**: Periods of low coordination may serve exploratory functions, allowing individuals to recover resources or explore alternative action modes.
- **Facilitator interventions**: The timing of facilitation may be most effective near transition points between synchronization and desynchronization states.

Importantly, empowerment synchrony ($R \approx 0.99$) and belief synchrony ($R \approx 0.998$) remain high throughout, indicating that action-level variability coexists with convergent internal states. This dissociation suggests that agents develop shared beliefs about the collective trust state while maintaining behavioral diversity—a pattern consistent with "thinking alike while acting differently."

1. Kaneko, K. Clustering, coding, switching, hierarchical ordering, and control in a network of chaotic elements. *Physica D* **1990**, *41*, 137–172.
2. Tsuda, I. Chaotic itinerancy as a dynamical basis of hermeneutics in brain and mind. *World Futures* **1991**, *32*, 167–184.
3. Tsuda, I. Dynamic link of memory—Chaotic memory map in nonequilibrium neural networks. *Neural Netw.* **1992**, *5*, 313–326.
4. Tsuda, I. Chaotic itinerancy. *Scholarpedia J.* **2013**, *8*, 4459.
5. Mierski, N.; Pilarczyk, P. Analysis of the chaotic itinerancy phenomenon using entropy and clustering. *arXiv [nlin.CD]* **2025**.
6. Namikawa, J. Chaotic itinerancy and power-law residence time distribution in stochastic dynamical system. *arXiv [nlin.CD]* **2004**. Published as Phys. Rev. E 72, 026204 (2005)., https://doi.org/10.1103/PhysRevE.72.026204.

7. Sauer, T. Chaotic itinerancy based on attractors of one-dimensional maps. *Chaos* **2003**, *13*, 947–952.
8. Tsuda, I.; Umemura, T. Chaotic itinerancy generated by coupling of Milnor attractors. *Chaos* **2003**, *13*, 937–946.
9. Fujimoto, K.; Kaneko, K. Bifurcation cascade as chaotic itinerancy with multiple time scales. *Chaos* **2003**, *13*, 1041–1056.
10. Friston, K.; Rigoli, F.; Ognibene, D.; Mathys, C.; Fitzgerald, T.; Pezzulo, G. Active inference and epistemic value. *Cogn. Neurosci.* **2015**, *6*, 187–214.
11. Kuramoto, Y. Chemical Turbulence. In *Chemical Oscillations, Waves, and Turbulence*; Springer Series in Synergetics, Springer Berlin Heidelberg: Berlin, Heidelberg, 1984; pp. 111–140.
12. Strogatz, S.H. From Kuramoto to Crawford: exploring the onset of synchronization in populations of coupled oscillators. *Physica D* **2000**, *143*, 1–20.
13. Tsuda, I.; Fujii, H.; Tadokoro, S.; Yasuoka, T.; Yamaguti, Y. Chaotic itinerancy as a mechanism of irregular changes between synchronization and desynchronization in a neural network. *J. Integr. Neurosci.* **2004**, *3*, 159–182.
14. Leyva, I.; Sendina-Nadal, I.; Letellier, C.; Sevilla-Escoboza, J.R.; Vera-Aila, V.P. From chaotic itinerancy to intermittent synchronization in complex networks. *arXiv [nlin.AO]* **2025**.
15. Schreiber, T. Measuring Transfer Entropy. *Physical Review Letters* **2000**, *85*, 461–464. https://doi.org/10.1103/PhysRevLett.85.461.
16. Da Costa, L.; Tenka, S.; Zhao, D.; Sajid, N. Active inference as a model of agency. *arXiv [cs.AI]* **2024**.
17. Jaakkola, T.S.; Jordan, M.I. Bayesian Parameter Estimation via Variational Methods. *Stat. Comput.* **2000**, *10*, 25–37.
